# A multifunctional dihydromyricetin-loaded hydrogel for the sequential modulation of diabetic wound healing and glycemic control

**DOI:** 10.1093/burnst/tkaf024

**Published:** 2025-03-19

**Authors:** Hongyi Li, Huiyun Wen, He Zhang, Xiang Cao, Li Li, Xiaowen Hu, Yanmei Zhang, Xinkun Shen, Quazi T H Shubhra, Hong Yang, Xiaojun Cai

**Affiliations:** Department of Chemical Engineering, Northwest University, No. 229, Taibai North Road, Beilin District, Xi’an City, Xi’an 710069, China; School and Hospital of Stomatology, Wenzhou Medical University, No. 268, Xueyuan West Road, Lucheng District, Wenzhou 325027, China; Department of Chemical Engineering, Northwest University, No. 229, Taibai North Road, Beilin District, Xi’an City, Xi’an 710069, China; Department of Chemical Engineering, Northwest University, No. 229, Taibai North Road, Beilin District, Xi’an City, Xi’an 710069, China; Department of Chemical Engineering, Northwest University, No. 229, Taibai North Road, Beilin District, Xi’an City, Xi’an 710069, China; State Key Laboratory of Oncology in South China, Collaborative Innovation Center for Cancer Medicine, Sun Yat-sen University Cancer Center, Sun Yat-sen University, No. 651, Dongfeng East Road, Yuexiu District, Guangzhou 510060, China; School and Hospital of Stomatology, Wenzhou Medical University, No. 268, Xueyuan West Road, Lucheng District, Wenzhou 325027, China; School and Hospital of Stomatology, Wenzhou Medical University, No. 268, Xueyuan West Road, Lucheng District, Wenzhou 325027, China; Department of Endocrine, Ruian People’s Hospital, The Third Affiliated Hospital of Wenzhou Medical University, No. 108, Wansong Road, Ruian City, Wenzhou 325200, China; Institute of Chemistry, University of Silesia in Katowice, Szkolna 9, Katowice 40-006, Poland; Department of Endocrine, Ruian People’s Hospital, The Third Affiliated Hospital of Wenzhou Medical University, No. 108, Wansong Road, Ruian City, Wenzhou 325200, China; School and Hospital of Stomatology, Wenzhou Medical University, No. 268, Xueyuan West Road, Lucheng District, Wenzhou 325027, China

**Keywords:** Chronic diabetic wound, Multifunctional hydrogel, Dihydromyricetin (DMY), Sequential therapeutic modulation, Glycemic control, Antioxidant and anti-inflammatory effects

## Abstract

**Background:**

The management of chronic diabetic wounds remains a formidable challenge in clinical practice. Persistent hyperglycemia triggers vasculopathy, neuropathy, and immune dysfunction, critically impeding wound repair. We developed a multifunctional hydrogel (DPFI) engineered for sequential therapeutic actions, including antibacterial, anti-inflammatory, antioxidant, pro-vascularization/epithelialization, and glycemic-regulating properties, to address these complications.

**Methods:**

DPFI hydrogels were prepared by encapsulating dihydromyricetin (DMY) into aldehyde-functionalized Pluronic F127 micelles (DMY@PF127-CHO), followed by a Schiff base reaction with amine-rich polyethyleneimine (PEI), resulting in the formation of a hydrogel for controlled drug release. The antimicrobial, antioxidant, anti-inflammatory, pro-cellular proliferative, and angiogenic properties of the hydrogels were evaluated using various techniques, including structural characterization, bacterial live/dead staining, reactive oxygen species (ROS) assays, antioxidant enzyme assays, reverse transcription–polymerase chain reaction (RT–PCR), cellular immunofluorescence staining, scratch wound healing assays, and angiogenesis assays. *In vivo*, the effects of the hydrogel on wound healing and glycemic control were assessed in methicillin-resistant *Staphylococcus aureus*-infected mice with streptozotocin-induced diabetes.

**Results:**

The hydrogel exhibits exceptional injectability, bioadhesion, and self-healing properties, facilitating the controlled, sustained release of DMY, which synergistically enhances antimicrobial effects in combination with PEI. The antioxidant activity of DMY is remarkable; it effectively scavenges ROS and induces the expression of antioxidant enzymes while promoting the phenotypic switch of M1 macrophages to M2 macrophages to mitigate inflammation. Critically, DPFI also contributes to glycemic regulation, reducing hyperglycemia-associated complications and creating a microenvironment conducive to wound repair. Comprehensive *in vitro* and *in vivo* analyses corroborate the multifaceted therapeutic capabilities of DPFI, including its antibacterial activity and abilities to clear ROS, reduce inflammation, promote angiogenesis, promote epithelialization, and modulate blood glucose levels.

**Conclusions:**

DPFI represents a promising, integrative strategy for enhanced diabetic wound management, meriting further exploration for clinical application.

Highlights

- Multifunctional DPFI hydrogel promotes antimicrobial, anti-inflammatory, and antioxidant effects for diabetic wound healing.
- The controlled release of dihydromyricetin facilitates sustained delivery, thereby optimizing bioavailability and enhancing therapeutic efficacy.
- The scavenging of reactive oxygen species and immunomodulation mitigate oxidative stress and inflammation, thereby accelerating tissue regeneration.
- The inherent glycemic control mechanisms serve to attenuate the incidence of wound complications arising from hyperglycemic conditions.
- Pro-angiogenic and epithelialization effects improve vascularization and wound closure in diabetic mice.

## Background

Diabetes mellitus (DM) is a chronic metabolic disorder characterized by disruptions in the metabolism of carbohydrates, lipids, and proteins, primarily due to inadequate insulin secretion or insulin resistance in target cells [1]. By 2021, >536.6 million people globally suffered from diabetes, a figure projected to rise to a staggering 783.2 million by 2045 [2]. One of the most severe complications of diabetes is the development of chronic, recalcitrant wounds, particularly diabetic foot ulcers, which significantly contribute to increased morbidity and mortality [3, 4]. These wounds affect approximately one-third of diabetic patients, severely impairing their quality of life and imposing a substantial economic burden on global healthcare systems [5]. Consequently, accelerating the healing process of chronic diabetic wounds has emerged as a pivotal focus in biomedical research, with increasing efforts to develop innovative biomaterials and therapeutic strategies that can efficiently facilitate wound healing.

Wound healing typically proceeds through four overlapping phases: hemostasis, inflammation, proliferation, and remodeling [6, 7]. In chronic diabetic wounds, this process is critically impaired by a dysregulated microenvironment characterized by polymicrobial infection, excess ROS levels, persistent inflammation, angiogenic failure, defective tissue regeneration, and hyperglycemia, disrupting all healing phases [8–11]. Targeted therapeutic strategies must address this multifactorial dysfunction. Foremost, preventing bacterial infections is paramount to maintaining a sterile environment conducive to healing. Second, dysfunctional immune cells, particularly M1 macrophages and neutrophils, secrete an array of proinflammatory cytokines, driving unrestrained inflammation and oxidative stress, exacerbating ROS accumulation and thereby impeding wound repair [12, 13]. Antioxidant and anti-inflammatory interventions can effectively neutralize ROS and proinflammatory mediators, induce the expression of cytoprotective factors, and facilitate the M1-to-M2 macrophage phenotype switch, fostering a favorable milieu for tissue regeneration and neovascularization [14–16]. Angiogenesis is particularly vital for delivering nutrients and oxygen to the wound bed and supporting the formation of new granulation tissue [17]. Finally, tightly regulated blood glucose levels throughout the healing process are essential, as hyperglycemia further disrupts normal tissue repair mechanisms [18, 19]. Therefore, developing a comprehensive therapeutic strategy that integrates antimicrobial, antioxidant, anti-inflammatory, pro-regenerative, and glycemic control interventions is imperative for promoting the rapid healing of chronic diabetic wounds.

Dihydromyricetin (DMY), a bioactive flavonoid derived from *Ampelopsis grossedentata* (snake grape), exhibits diverse pharmacological activities, such as antimicrobial, anti-inflammatory, antioxidant, and pro-regenerative properties, which make this drug particularly promising for managing diabetic wounds [20–22]. DMY effectively targets pathogens such as *Staphylococcus aureus* (*S. aureus*) and *Escherichia coli* (*E. coli*), scavenges free radicals, mitigates oxidative stress, and modulates inflammatory pathways, facilitating wound healing through increased cell proliferation, migration, and angiogenesis. Furthermore, its glucose-lowering and insulin-sensitizing properties position DMY as a dual-action agent in both glycemic control and tissue repair [23–26]. However, the clinical application of DMY is limited by its poor water solubility, suboptimal stability (low temperatures, pH 6.0), and limited bioavailability, hindering its therapeutic efficacy at wound sites [27, 28]. Thus, advanced delivery systems enabling the controlled and sustained release of DMY are crucial for increasing its stability, permeability, and localized bioactivity, potentially broadening its utility in chronic diabetic wound care.

Due to their 3D cross-linked water–polymer networks, hydrogels emulate the extracellular matrix and have garnered considerable attention in biomedical applications [29, 30]. The high-water content, strong adhesion, flexibility, and mechanical strength of these materials, along with excellent breathability, moisture retention, and biocompatibility, make them exceptional candidates for wound dressings [31, 32]. A remarkable feature of hydrogels is their potential as drug delivery systems. They can encapsulate hydrophobic drugs and provide sustained, localized release through slow-release mechanisms, improving drug efficacy, reducing the need for frequent reapplication, and enhancing wound healing outcomes for diabetic patients [33, 34]. Despite their potential, current hydrogel dressings are limited in providing sustained wound protection and dynamically adapting to the changing wound microenvironment, often resulting in secondary infections, inflammation, and delayed regeneration [35, 36]. As methods to address these challenges, researchers have integrated functional nanoparticles, monomers, or bioactive agents into hydrogels to introduce multifunctional therapeutic effects, including antimicrobial, antioxidant, anti-inflammatory, and angiogenic effects [37–39]. Nevertheless, a fundamental limitation persists: most engineered hydrogels exhibit only one or two therapeutic benefits and cannot comprehensively regulate the complex microenvironment of diabetic wounds. This finding highlights the primary unmet need in current research: developing advanced hydrogels with integrated multiple therapeutic functions—such as antimicrobial activity, antioxidant activity, and the abilities to modulate inflammation, cell proliferation, angiogenesis, and glycemia—within programmable treatment platforms for effective chronic wound management.

**Figure 1:**
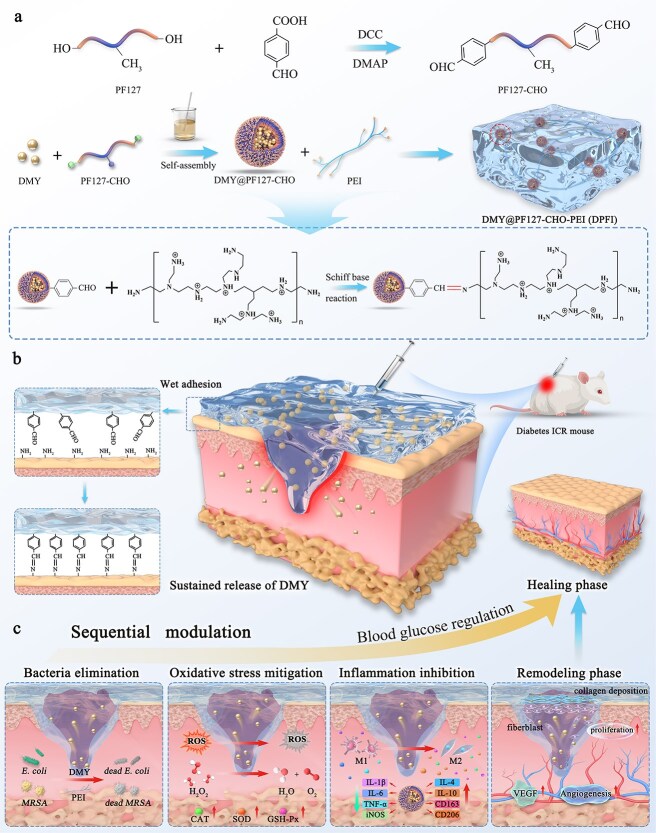
Overview of DPFI hydrogel programmed for diabetic chronic wound treatment. (**a**) Synthesis route of PF127-CHO and the subsequent formation of the DPFI hydrogel. (**b**) Illustration showing the key features of the DPFI hydrogel, including its adhesion ability, injectability, and sustained drug release properties. (**c**) Programmed therapeutic strategy of the DPFI hydrogel, targeting antimicrobial activity, antioxidant and anti-inflammatory effects, and promoting cell proliferation, migration, and vascularization to facilitate accelerated wound healing

Here, we report the development of a highly innovative and versatile hydrogel dressing, DMY@PF127-CHO-PEI (DPFI), which was engineered for the programmed treatment of chronic diabetic wounds (Figure 1). DPFI is synthesized through a Schiff base reaction between the aldehyde groups of PF127-CHO and the amino groups of PEI, a cationic polymer known for its abundant amine groups and natural antimicrobial properties [40]. In this construct, PF127-CHO, a biocompatible and thermosensitive block copolymer, was selected as the hydrogel matrix because of its ability to form micelles and increase the solubility of DMY [41]. Dynamic Schiff base bonding endows DPFI with excellent injectability, self-healing capabilities, and the sustained, controlled release of DMY. Upon contact with infected wounds, PEI rapidly exerts antimicrobial effects. As the hydrogel degrades, DMY is released, effectively scavenging ROS, activating antioxidant enzymes, and promoting the polarization of M1 macrophages to the M2 phenotype. This process reduces cytokine expression and inflammation, further promoting angiogenesis, fibroblast proliferation, and wound healing. Moreover, sustained DMY release aids in glycemic regulation, optimizing wound recovery.

To the best of our knowledge, DPFI is the first hydrogel system uniquely designed to integrate DMY, PEI, and PF127-CHO into a multifunctional scaffold. This hydrogel seamlessly executes sequential therapeutic effects, targeting key aspects of diabetic wound healing—including antimicrobial action, oxidative stress mitigation, inflammation resolution, tissue regeneration, angiogenesis, and glycemic modulation. A series of comprehensive *in vitro* and *in vivo* experiments revealed the multifaceted capabilities of DPFI, including its antibacterial, antioxidant, and anti-inflammatory properties, as well as its ability to promote cell proliferation, vascularization, and blood glucose regulation. These findings collectively position DPFI as a highly promising therapeutic platform with the potential to redefine the treatment paradigm for chronic diabetic wounds, offering a clinically relevant and translational solution to a complex and persistent medical challenge.

## Methods

### Reagents

Pluronic F-127 (Mw, 12 600 Da), tetrahydrofuran (99%), 4-formylbenzoic acid (98%), N, N′-dicyclohexylcarbodiimide (DCC, 99%), and 4-dimethylaminopyridine (DMAP 99%) were purchased from Aladdin (Shanghai, China). PEI (Mw, 1800 Da) and DMY (99%) were sourced from Yuanye Bio (Shanghai, China). Calcein-AM, Hoechst 33342, and Cell Counting Kit-8 (CCK-8) were obtained from Keygen Biotech Co. Ltd (Nanjing, China). 2′,7′-Dichlorodihydrofluorescein diacetate (DCFH-DA) was obtained from Beyotime. ROSGreen H_2_O_2_ Probe was obtained from Maokang Bio (China). SYTO-9/PI double staining kits was sourced from Invitrogen (USA). Polyphenol oxidase (PPO) assay kit, superoxide dismutase (SOD) assay kit, catalase (CAT) assay kits and glutathione peroxidase (GSH-Px) assay kit were purchased from Grace Biotechnology (Suzhou, China). Mouse IL-6, Mouse IL-1β, and Mouse TNF-α ELISA Kits were purchased from Shanghai Jianglai Biotech. Co., Ltd Antibodies against iNOS and CD206 were obtained from Proteintech (USA). The primers for Mouse HO-1, Mouse IL-6, Mouse IL-1β, Mouse TNF-α, Mouse IL-4, Mouse IL-10, and Mouse CD163 were purchased from Tsingke Biotech. Co., Ltd RAW264.7 murine macrophages were obtained from the American Type Culture Collection (ATCC). NCTC clone 929 cells (L929) and human umbilical vein endothelial cells (HUVECs) were obtained from Otwo Biotech. Methicillin-resistant *Staphylococcus aureus* (MRSA) and *E. coli* were obtained from the ATCC.

### Synthesis and characterization of benzaldehyde-terminated PF127

A mixture of 12.6 g of Pluronic F-127 (1 mmol), 0.9 g of 4-formylbenzoic acid (6 mmol), 1.24 g of DCC (6 mmol), and 0.074 g of DMAP (0.6 mmol) was dissolved in 150 ml of tetrahydrofuran. The reaction was allowed to proceed at room temperature for 24 hours to ensure complete conjugation. Post-reaction, the solvent was removed via rotary evaporation at 60°C. To the residue, 200 ml of water was added, and the resulting precipitate was filtered out. The filtrate was then dialyzed against distilled water for 4 days using a dialysis bag (MWCO = 3500 Da) to remove impurities. Finally, the product, PF127-CHO, was obtained by freeze-drying. The structures of F127 and F127-CHO were characterized using a Nicolet 6700 FT-IR spectrometer (Thermo Scientific) and a Bruker Avance III 400 MHz ^1^H NMR spectrometer (Bruker).

DMY@PF127-CHO micelles were synthesized by dissolving 10 mg of DMY and 500 mg of PF127-CHO in N, N-Dimethylformamide (DMF), followed by stirring until complete dissolution. The solution was subsequently transferred into a dialysis bag (MWCO = 3500 Da) and dialyzed for 2 Days to obtain micelles loaded with DMY (DMY@F127-CHO). The zeta potential and particle size of DMY@F127-CHO were measured using dynamic light scattering (DLS, Nano ZS90). The morphology of DMY@F127-CHO was analyzed by transmission electron microscopy (TEM, JEM-2100Plus). DMY loading content and efficiency were quantified using a UV–Vis spectrophotometer (Multiskan Go, Thermo Scientific) based on a standard curve of DMY.

### Fabrication and characterization of drug free hydrogels (PFI) and dihydromyricetin-loaded DPFI hydrogels

To prepare PFI hydrogels, 3% PEI and 22% PF127-CHO solutions were mixed in volume ratios of 2:1, 4:1, 6:1, and 8:1, forming hydrogels via Schiff base reactions. These were designated as PFI 2.0, PFI 4.0, PFI 6.0, and PFI 8.0, respectively. Gelation time was assessed using the inverted bottle method, where the time for the liquid to stop flowing and solidify into a gel was recorded.

For the preparation of DMY-loaded DPFI hydrogels, 5 mg, 10 mg, and 15 mg of DMY were dissolved with 500 mg of PF127-CHO in DMF, stirred until completely dissolved, and then dialyzed for 2 days in a dialysis bag (MWCO = 3500 Da) to form DMY-loaded micelles. These micelles were then reacted with 3% PEI to produce DPFI-1, DPFI-2, and DPFI-3 hydrogels, following the same gel formation procedure. The hydrogels were freeze-dried, and their morphology was analyzed using scanning electron microscopy (SEM, Hitachi SU8010). Porosity was calculated using ImageJ software.

Rheological properties of hydrogels, including injectability and self-healing ability, were evaluated using a HAAKE MARS rheometer (Thermo Mars40.USA). The loss (G″) and storage modulus (G′) of the PFI and DPFI-3 hydrogels were measured using strain-scanning measurements in the range of 1% to 1000%, with a constant frequency of 1 rad/s. The shear-thinning measurements were carried out at shear rates ranging from 0.1 s^−1^ to 1000 s^−1^. with strain and frequency fixed at 1% and 1 Hz, respectively. Macroscopic assessment of PFI injectability was accomplished by filling a 1 ml syringe with 1 ml of PFI hydrogel labeled with rhodamine B and then extruding it into a petri dish to form a font pattern.

The self-healing properties of PFI and DPFI-3 hydrogels were examined at 25°C. PFI hydrogel specimens with a diameter of 8 mm and a height of 1 mm were selected and placed on the rheometer test rig. The specimens were examined using the time-scan test mode of the instrument, with the stress amplitude set between 1% and 1000% and the frequency set at 1 Hz. The energy storage modulus (G′) and loss modulus (G″) of the hydrogels were recorded as a function of applied stress to determine their critical strain points. The hydrogels were then tested in an alternating time-scanning mode, where the angular frequency was set to 1 rad/s, and the strain levels were varied from 1% to 200%, 600%, and 800%, with a period of 60 seconds for each cycle. This rotational process was repeated three times, and the variations in G′ and G″ were continuously recorded. Macroscopic self-healing experiments were conducted using two circular hydrogels (10 × 1 mm^2^) labeled with rhodamine B and sodium fluorescein. Each hydrogel was cut into two halves, and the halves of different colors were then spliced together without any external stimuli. The hydrogels were left to stand for 4 hours to observe spontaneous self-repair, and photographs were taken to document the process.

The adhesive properties of the PFI and DPFI-3 hydrogels were evaluated using a lap-shear test. Hydrogel precursor solution was applied to the surface of pigskin (10 × 10 mm^2^) and allowed to solidify at room temperature for 2 hours. The tests were then performed on a universal material testing machine with a maximum force capacity of 50 N and an application speed of 20 mm/min. To further assess macroscopic bonding properties, a 100 g weight was suspended from the lower end of the pigskin to observe the hydrogel’s adhesion to various surfaces, including plastic, wood, glass, rubber, and metal.

Degradation studies were performed by immersing lyophilized hydrogels (*W_0_*) in 20 ml of PBS solution (pH 7.4) at 37°C, with continuous shaking. Periodically, the hydrogels were removed, subjected to lyophilization, and weighed (*W*_t_), and the degradation rate of the hydrogels was calculated using the following equation:


\begin{equation*}\text{Degradation}\ \text{rate}=\frac{W_t-{W}_0}{W_0}\times 100\% .\end{equation*}

At 37°C, 500 ml of DPFI-1, DPFI-2, and DPFI-3 hydrogels were each placed in a dialysis bag (MWCO = 3500 Da) and immersed in 20 ml of PBS (pH = 7.4). At various time points, 1 ml of dialysate was collected and replaced with an equal volume of fresh PBS. The absorbance of the dialysate was measured using a UV spectrophotometer to determine the drug release concentration.

### Cell viability

In this study, L929 cells were used for cytotoxicity assessment. Cells were seeded at a density of 8 × 10^3^ cells per well in a 96-well plate. After 24 hours of incubation at 37°C, 200 μl of hydrogel extract (prepared following previous protocol [42]) was added to each well, and incubation continued for an additional 24 hours. Following this, the culture medium was removed, and the cells were washed three times with PBS. using CCK-8 reagent was then added, and the cells were incubated for 30 minutes. The optical density at 450 nm was measured using a microplate reader. The optimal proportion of PFI 6.0 hydrogel obtained from screening was then used to load DMY with different drug concentrations. The same cytotoxicity assay was performed with these samples. To visually assess cell viability, L929 cells were double-stained with calcein-AM (for live cells) and propidium iodide (PI; for dead cells). The stained cells were observed and imaged using an inverted fluorescence microscope (AxioObserver3 ZEISS) to evaluate live/dead cell populations.

### *In vitro* antimicrobial activity of DPFI

The antimicrobial properties of the DPFI hydrogels were evaluated using *E. coli* and MRSA as model organisms. Initially, suspensions of *E. coli* and MRSA, each with a concentration of 10^9^ CFU/mL, were inoculated into 96-well plates and co-cultured with the hydrogel precursor solutions for 12 hours. Following incubation, the bacterial suspensions were collected, diluted to 10^5^–10^1^ CFU/mL, and 5 μl of each dilution was plated on TSB agar plates, and subjected to colony-forming unit (CFU) quantification counts. The plates were incubated at 37°C for 12 hours to allow for bacterial growth. Bacterial survival was assessed by calculating the survival rate. To visualize bacterial viability, hydrogel-treated bacterial samples were stained with PI (for dead cells) and SYTO-9 (for live cells) dyes and incubated in the dark for 30 minutes before observation under a fluorescence microscope (NI-B, Nikon). To investigate bacterial morphology, *E. coli* and MRSA were co-cultured with the hydrogels, fixed with 2.5% glutaraldehyde, and subjected to gradient ethanol dehydration. The bacterial surface structure and integrity were examined using SEM. PBS-treated bacteria served as the control group, and each experimental condition was replicated in triplicate.

### *In vitro* antioxidant activity of DPFI

In total, 200 μl of DPFI hydrogels were evenly spread in 24-well plates, and their antioxidant capacity was initially assessed using PPO, SOD, and CAT assay kits. To evaluate the antioxidant properties of DPFI hydrogels at the cellular level, RAW264.7 macrophages (2.5 × 10^7^ cells) were first seeded in a large Petri dish (10 × 10 cm), cultured in Dulbecco’s modified eagle medium (DMEM) medium containing 10% (v/v) FBS and 1% (v/v) penicillin–streptomycin, and incubated for 24 hours in a CO_2_ incubator. After medium removal, the cells were treated with extracts from PFI, DPFI-1, DPFI-2, and DPFI-3 hydrogels containing lipopolysaccharides (LPS, 3 μg/ml) and incubated for an additional 20 hours. Following treatment, the medium was discarded, and the cells were washed with PBS, centrifuged, and resuspended in 1 ml of PBS. The cells were lysed, and protein concentrations were determined. The activities of various intracellular antioxidant enzymes were measured according to the instructions provided with the SOD, CAT, and GSH-Px assay kits. DMEM-treated normal macrophages and activated macrophages without hydrogel treatment served as controls.

For assessing ROS levels in activated macrophages following DPFI treatment, a DCFH-DA probe was employed. Briefly, RAW 264.7 cells were seeded into glass-bottomed dishes (35 × 35 mm) at a density of 1 × 10^6^ cells per well and incubated for 24 hours in a CO₂ incubator. After incubation, the medium was replaced with extracts from PFI, DPFI-1, DPFI-2, and DPFI-3 hydrogels, each containing LPS (3 μg/ml), and the cells were incubated for an additional 20 hours. The medium was then discarded and replaced with fresh medium containing the DCFH-DA probe, followed by a 30-minute incubation in the dark. The cells were washed three times with PBS, and ROS generation was observed and recorded using inverted fluorescence microscopy to detect green fluorescence. A similar protocol was used to evaluate hydrogen peroxide (H₂O₂) release from macrophages after hydrogel treatment, utilizing an H₂O₂-specific probe. The expression levels of HO-1 were quantified through an RT-PCR assay, following the protocol previously reported in the literature [36]. Normal macrophages treated with DMEM and activated macrophages without hydrogel treatment served as controls.

### *In vitro* anti-inflammatory activity of DPFI

To assess the anti-inflammatory properties of DPFI, RAW 264.7 cells were seeded into glass-bottomed dishes (35 × 35 mm) at a density of 5 × 10^5^ cells per well and incubated for 24 hours. After incubation, the medium was replaced with extracts from PFI, DPFI-1, DPFI-2, and DPFI-3 hydrogels, each containing LPS (3 μg/ml), followed by an additional 20-hour incubation. Post-treatment, cell morphology was observed using an inverted fluorescence microscope. The elongation ratio and synaptic number of macrophages were calculated as described in previous studies [43]. Additionally, the cells were immunolabeled with antibodies against CD163 and iNOS and visualized under confocal laser scanning microscope. Expression levels of pro-inflammatory factors (IL-6, IL-1β, TNF-α) and anti-inflammatory factors (IL-4, IL-10, CD163) were measured using a real-time fluorescence quantitative PCR system. The primers used are listed in Table S3 of the Supporting Information.

### *In vitro* cell proliferation, migration, and vascularization activities of DPFI

For the evaluation of cellular proliferation and migration, L929 cells were utilized. In proliferation assays, L929 cells were seeded at a density of 8 × 10^3^ cells per well in 96-well plates and cultured for 24 hours. The medium was then replaced with extracts derived from PFI, DPFI-1, DPFI-2, and DPFI-3 hydrogels, and the cells were incubated for 1 or 3 days. After incubation, the cells were washed three times with PBS and then exposed to CCK-8 reagent. The plates were incubated in the dark for 30 minutes, after which absorbance was measured at 450 nm using a microplate reader. The cells were also stained with calcein-AM for 30 minutes and observed using an inverted fluorescence microscope.

For the cell scratch assay, L929 cells (1 × 10^5^ cells/well) were first seeded into a 24-well plate and cultured for 24 hours. After that, a scratch, ~2 mm wide, was created using a sterile pipette tip. The cells were then exposed to 1 ml of extracts from PFI, DPFI-1, DPFI-2, and DPFI-3 hydrogels for an additional 24 or 48 hours. After incubation, the cells were stained with crystal violet (CV) and observed using an inverted fluorescence microscope.

To assess the vasculogenic potential of DPFI hydrogels, HUVECs (1 × 10^5^ cells/well) were first cultured in a 6-well plate for 24 hours. The medium was then replaced with 1 ml of extracts derived from PFI, DPFI-1, DPFI-2, and DPFI-3 hydrogels, and the cells were incubated for an additional 24 hours. Following this, the cells were trypsinized and reseeded in 96-well plates (3 × 10^4^ cells/well) pre-spread with matrix gel for 4 hours. After incubation, the cells were observed using an inverted fluorescence microscope. The number of nodes and branches was computed by ImageJ.

### *In vivo* treatment of methicillin-resistant *Staphylococcus aureus*-infected streptozotocin-induced diabetic wounds

ICR mice (male, 8 weeks old) were purchased from Beijing SPF Biotechnology Co. All procedures involving animals followed the guidelines set by the Chinese National Institutes of Health and were approved by the Animal Care and Use Committee of Wenzhou Medical University (protocol number: wydw2024–0509). To establish a chronic wound model in MRSA-infected diabetic mice, the mice were initially administered streptozotocin (STZ, 80 mg/kg) for 4 days. When blood glucose levels reached or exceeded 16.7 mM, the mice were anesthetized with 3% sodium pentobarbital (70 mg/kg). Their dorsal fur was then shaved, and an 8 mm × 8 mm full-thickness cutaneous wound was created on each mouse’s back. Subsequently, 10 μl of MRSA solution (10^9^ CFU/mL) was inoculated into the wound. The mice were divided into five groups (n = 6/group): (1) PBS (control); (2) PFI 6.0 hydrogel; (3) DPFI-1 hydrogel; (4) DPFI-2 hydrogel; and (5) DPFI-3 hydrogel. PBS (50 μl) and hydrogel samples (50 μl) were either directly applied to or extruded into the wound area.

Blood glucose levels, wound area contraction, and body weight of the mice were measured at predetermined time points to monitor the healing progression. The wound healing rate was calculated using the formula:


$$ \mathrm{Wound}\ \mathrm{healing}\ \mathrm{rate}\%=\left({\mathrm{S}}_0-{\mathrm{S}}_{\mathrm{t}}\right)/{\mathrm{S}}_0\times 100\% $$

where S_0_ is the wound area on day 0 and S_t_ is the wound area at a given day.

To evaluate the *in vivo* antimicrobial activity of DPFI, the wound tissues were collected on day 2, homogenized, and subjected to CFU quantification on TSB agar plates. For assessing *in vivo* anti-inflammatory and antioxidant activities, blood was collected on day 8, centrifuged to obtain the supernatant, and the levels of IL-1β, TNF-α, and IL-6 were measured using respective ELISA kits. In addition, peri-wound tissues were also collected on day 8 for immunofluorescence staining (with iNOS, CD163, and HO-1 antibodies) to examine inflammatory and angiogenic markers.

On day 15, the mice were euthanized, and wound tissues along with vital organs were collected for histological analysis, including Masson’s trichrome (MT) staining, hematoxylin and eosin (H&E) staining and immunohistochemistry (with CD31 antibody). Routine blood analyses were performed using standard techniques provided by Wuhan Servicebio Biotech. Co. [44].

### Statistical analysis

The results are presented as mean ± SD, with all data derived from at least three independent experiments. One-way analysis of variance (ANOVA) and two-way ANOVA were used to analyze the data. Post hoc pairwise comparisons were performed using Tukey’s test. Data analysis was carried out using Prism software (version 9.0; GraphPad, La Jolla, CA, USA). Statistical significance was defined as *P* <.05 (^*^  *P* <.05, ^**^  *P* ≤.01, ^***^  *P* ≤.001). A notation of ‘ns’ represents a lack of statistical significance.

## Results

### Synthesis and characterization of PFI and DPFI hydrogels

PF127-CHO was initially synthesized through an esterification reaction between 4-formylbenzoic acid and Pluronic F-127 to prepare DMY@PF127-CHO micelles. As depicted in Figure S1, successful synthesis was confirmed by the appearance of a new carbonyl stretching peak at 1720 cm^−1^ in the FT-IR spectrum, indicating the formation of aldehyde groups. ^1^H NMR spectroscopy provided further validation, with the emergence of a distinct peak at 10.09 ppm, corresponding to the protons on the aldehyde group (-CHO) (Figure S2). By comparing the relative integration of the hydrogen atoms on the aldehyde groups with those on F127, we estimated that ~67.46% of the hydroxyl groups on F127 were replaced with aldehyde groups.

**Figure 2:**
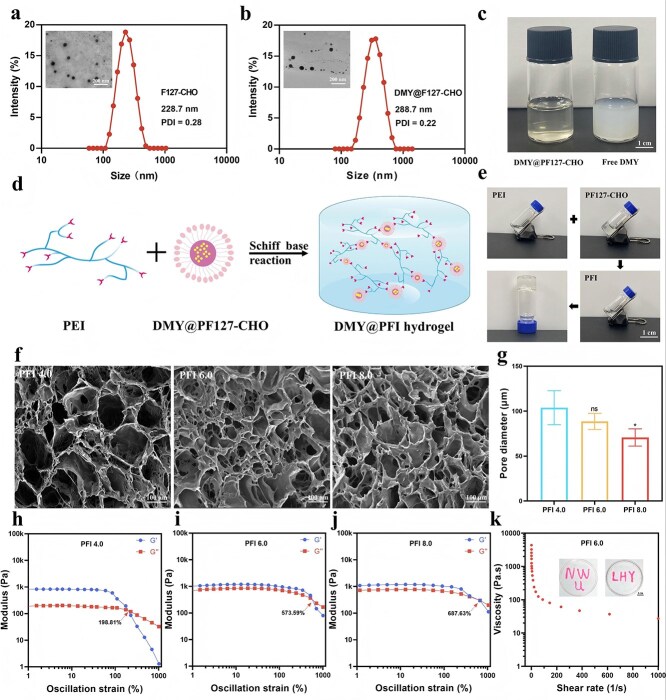
Physicochemical characterization of PFI hydrogels. (**a** and **b**) Particle size distribution and TEM images of PF127-CHO and DMY@PF127-CHO nano-micelles. (**c**) Comparison of solubility of DMY@F127-CHO (left) and free DMY (right) in water (scale bar: 1 cm). (**d**) Schematic representation illustrating the formation of the hydrogel via the Schiff base reaction between PF127-CHO and PEI. (**e**) Visualization of hydrogel gelation using the tubule inversion method (scale bar: 1 cm). (**f** and **g**) SEM images and pore size distributions of PFI-n hydrogels (scale bar: 100 μm). (**h**-**j**) Rheological analysis showing the storage modulus (G′) and loss modulus (G″) trends of PFI-n hydrogels as a function of strain. (**k**) Shear viscosity measurements alongside a photograph demonstrating the injectability of the PFI 6.0 hydrogel. ^*^P <0.05, ns: no significance. In (g), comparisons are between the PFI 4.0 group and other groups. *PDI* polymer dispersity index

After the successful preparation of PF127-CHO, we investigated the encapsulation efficiency of DMY within the micelles. Dynamic light scattering (DLS) measurements revealed that the hydrated particle size of the DMY@PF127-CHO micelles was 288.7 nm, with a polydispersity index (PDI) of 0.22 (Figure 2a and b), indicating a uniform size distribution compared with that of the blank PF127-CHO micelles (228.7 nm, PDI = 0.28). The TEM images in Figure 2a and b (insert) show that DMY@PF127-CHO had a well-defined 3D spherical nanostructure, with a mean diameter of ~200 nm.

We qualitatively assessed the increase in solubility, and hydrophobic DMY was found to be poorly soluble in water, forming a white, turbid suspension (Figure 2c, right panel). However, when DMY and PF127-CHO were dissolved in water simultaneously, a yellow transparent liquid formed (Figure 2c, left panel). A quantitative analysis through UV–vis absorption spectroscopy at 292 nm indicated a high drug-loading efficiency of 80.4% for DMY when the DMY/F127-CHO feeding ratio was 3:100 (Table S1).

After successfully preparing DMY/F127-CHO micelles, we next explored the formation of hydrogels through a Schiff base reaction between the amino groups on PEI and the aldehyde groups on F127-CHO micelles (Figure 2d). Using the tubular tilting method (Figure 2e), we observed a rapid sol–gel transition upon mixing equimolar volumes of PF127-CHO micelles and PEI, resulting in the formation of a hydrogel designated PFI. Hydrogel formation was confirmed by FT-IR analysis, which revealed a marked decrease in the aldehyde peak at 1720 cm^−1^ in PF127-CHO after reacting with PEI. Simultaneously, a new peak corresponding to the imine bond appeared at 1645 cm^−1^, indicating that the Schiff base reaction between PEI and PF127-CHO resulted in hydrogel formation (Figure S3).

The kinetics of gelation revealed a decreasing gelation time with increasing PF127-CHO content, as summarized in Table S2. For example, while PFI 2.0 remained in a sol state after 9 minutes, PFI 8.0 formed a gel within only 22 seconds. We investigated the effect of the PF127-CHO content on the crosslinked network of the hydrogel by employing SEM to examine the microstructure of the hydrogels and ImageJ to calculate the pore size (Figure 2f). An increase in the F127-CHO proportion resulted in a denser hydrogel structure and smaller pore sizes, with average pore sizes of 103.89 ± 18.93 μm, 88.58 ± 8.94 μm, and 70.74 ± 9.61 μm for the three different compositions (PFI 4.0, PFI 6.0, and PFI 8.0, Figure 2g), respectively. Additionally, the pore size of the DPFI-3 hydrogel was similar to that of PFI 6.0, with a dense structure and an average pore size of 87.94 ± 6.26 μm (Figure S4a and b).

The mechanical robustness of the PFI hydrogels was evaluated through a rheological analysis. As depicted in Figure 2h–j, the energy storage modulus (G′) and loss modulus (G″) were evaluated across a strain range of 1% to 1000% for PFI 4.0, PFI 6.0, and PFI 8.0. The critical destructive strains were 198.81%, 573.59%, and 687.63%, respectively. Furthermore, the inclusion of the drug did not significantly alter the critical breaking strain of the hydrogel, with DPFI-3 showing a critical breaking strain of 587.55% (Figure S5). Additionally, we assessed the injectability of the PFI and DPFI-3 hydrogels through shear-thinning experiments. For PFI 6.0, the viscosity decreased from 4352.06 Pa.s to 27.24 Pa.s as the shear rate increased (Figure 2k), indicating excellent shear-thinning properties. Similar behavior was observed for PFI 4.0, PFI 8.0, and DPFI-3 (Figure S6), further confirming the favorable injectability characteristics of the hydrogels. Moreover, PFI 6.0 could be easily extruded from a syringe to form shapes such as ‘NEW’ and ‘LHY’ on Petri dishes (Figure 2k, insert).

**Figure 3:**
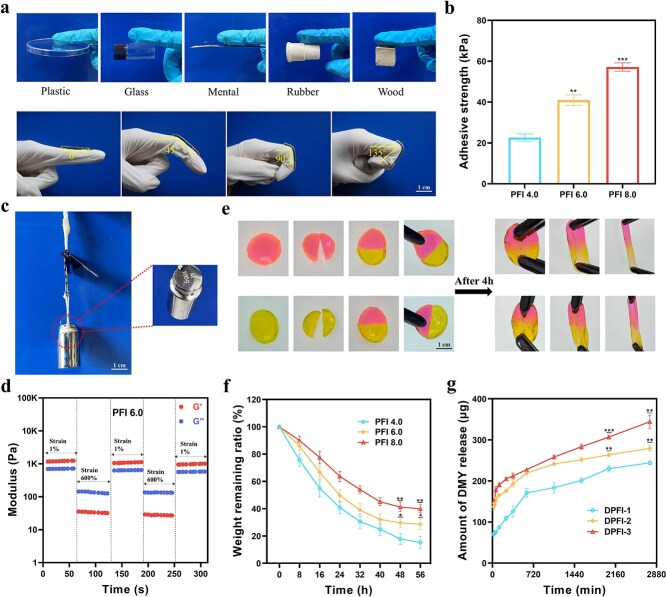
Characterization of PFI and DPFI hydrogels. (**a**) Photographs showing the adhesiveness of PFI hydrogel to various materials, illustrating its ability to adhere as the finger’s bending angle changes (scale bar: 1 cm). (**b**) Adhesion strength of PFI-n hydrogels on pig skin as a function of F127-CHO concentration. (**c**) Schematic representation of PFI adhesion to pigskin with 100 g weights attached (scale bar: 1 cm). (**d**) Rheological properties of PFI 6.0 as strains alternately change from 1% to 600%. (**e**) Macroscopic image demonstrating the self-healing capacity of PFI hydrogel (scale bar: 1 cm). (**f**) Degradation behavior of PFI-n hydrogels in PBS at pH 7.4. (**g**) Drug release behavior of DPFI-n hydrogel in PBS at pH 7.4. ^*^P <0.05, ^**^P <0.01, ^***^P <0.001. In figure b and figure f, comparisons are made between the PFI 4.0 group and other groups; in figure g, comparisons are made between the DPFI-1 group and other groups

The PFI hydrogels displayed strong adhesion to various materials, including plastic, glass, metal, rubber, and wood, as well as flexibility to conform to the surface of a finger during bending (Figure 3a). We further evaluated the adhesive performance by conducting lap shear experiments on fresh pig skin using different PFI and DPFI-3 hydrogel compositions. As shown in Figure 3b, the adhesion strength of the PFI hydrogels increased with increasing concentrations of F127-CHO, with values of 22.63 ± 1.77 kPa for PFI 4.0, 40.92 ± 2.60 kPa for PFI 6.0, and 57.15 ± 2.02 kPa for PFI 8.0. The drug-loaded hydrogel DPFI-3 exhibited an adhesion strength of 40.18 ± 1.69 kPa, which was comparable to that of PFI 6.0 (Figure S7). Additionally, these hydrogels could withstand a load of ~100 grams (Figure 3c), further confirming their robust adhesion properties.

In addition to their strong tissue adhesion, PFI hydrogels also exhibited impressive self-healing capabilities. Under large-amplitude oscillatory forces at 600% strain, the moduli G′ and G″ of PFI 6.0 decreased significantly, indicating a transition from a gel to a quasi-liquid state. However, when the strain amplitude was reduced to 1%, G′ rapidly returned to its original value without significant loss, highlighting the reversible nature of this behavior across multiple cycles (Figure 3d). This self-healing ability was also observed for the PFI 4.0, PFI 8.0, and DPFI-3 hydrogels, demonstrating the robust self-repair capacity of the PEI-based hydrogels (Figure S8). On a macroscopic level, the self-healing ability of PFI hydrogels was further validated by an experiment in which a hydrogel, cut into two parts, achieved complete self-repair after four hours of natural recombination without any external force applied (Figure 3e). After this self-healing process, the mechanical integrity of the hydrogel was maintained, as evidenced by its resistance to fracture during subsequent tensile testing. These results suggest that PFI hydrogels can self-repair while retaining their structural and mechanical properties, making them highly suitable for practical biomedical applications such as wound dressings.

Good biodegradability is a crucial property of drug-loaded hydrogel dressings, as it enables controlled drug release during hydrogel degradation and minimizes secondary damage to new skin tissues during dressing changes. We conducted detailed experiments on the degradation and drug release properties of the PFI and DPFI-3 hydrogels to assess these characteristics. As shown in Figure 3f, PFI 4.0 exhibited rapid degradation, with ~83.31% of the hydrogel breaking down within 56 hours of incubation in PBS. In contrast, PFI 6.0 and PFI 8.0 degraded more slowly, with only 66.87% and 57.10% degradation, respectively. The addition of the drug did not significantly affect the degradation rate of the hydrogel, with DPFI-3 exhibiting a degradation rate of 71.43% (Figure S9). Furthermore, the rapid degradation of the hydrogel facilitated the swift release of DMY from the drug-loaded DPFI hydrogels. The cumulative drug release increased with increasing DMY loading. As shown in Figure 3g, after 2760 minutes, DPFI-3 released up to 344.20 ± 16.20 μg of DMY, whereas the cumulative amounts of DPFI-1 and DPFI-2 released were 244.11 ± 4.89 and 279.40 ± 7.13 μg, respectively.

The cytocompatibility of both the blank (drug-free) and drug-loaded hydrogels with L929 cells was evaluated via CCK-8 assays. As shown in Figure S10a, all blank hydrogels (PFI 4.0, PFI 6.0, and PFI 8.0) exhibited minimal cytotoxicity, with cell survival rates exceeding 85%. However, as the concentration of PF127-CHO increased, the cell survival rates slightly decreased, with PFI 8.0 resulting in the lowest survival rate of 86.13%. For the drug-loaded hydrogels (DPFI-1, DPFI-2, and DPFI-3), the drug promoted cell growth at various concentrations. However, higher DMY concentrations gradually slowed the cell proliferation rate and caused some inhibition (Figure S10b). These results were further validated by live/dead cell assays. As shown in Figure S11, almost all the cells exhibited green fluorescence (live cells), with dead cells showing red fluorescence. These findings align with the results of the CCK-8 assay.

### *In vitro* antimicrobial activity of DPFI

Chronic diabetic wounds create an environment that promotes the proliferation of periwound flora, notably MRSA and *E. coli*, which complicates the wound healing process. We used the plate counting method to assess the antimicrobial activity of the hydrogels *in vitro*. As shown in Figure S12a–c, the antimicrobial effectiveness of the blank PFI hydrogels diminished as the PF127-CHO ratio increased, with PFI 8.0 exhibiting the weakest performance. Although PFI 4.0 displayed strong bactericidal activity, PFI 6.0 was selected for further experiments because of its superior mechanical properties. After 12 hours of treatment with PFI 6.0, the survival rates of MRSA and *E. coli* were 6.18 log10 CFUs/ml and 6.64 log10 CFUs/ml, respectively.

**Figure 4:**
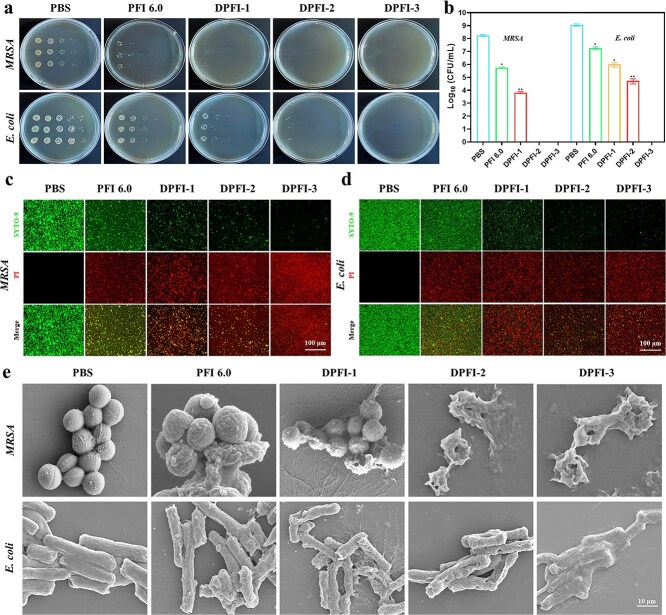
Antimicrobial properties of DPFI-n hydrogels. (**a** and **b**) Plate images and quantitative survival rates of MRSA and *E. coli* following treatment with DPFI-n hydrogels. (**c** and **d**) Live/dead staining results for MRSA and *E. coli*, with red fluorescence indicating cell death (scale bar: 100 μm). (**e**) SEM images showing the morphological changes in MRSA and *E. coli* after hydrogel treatment (scale bar: 10 μm). ^*^P <0.05, ^**^P <0.01. Comparisons are made between the PBS group and other groups

We then evaluated the antibacterial properties of the drug-loaded DPFI-n hydrogels *in vitro*. As shown in Figure 4a and b, the DPFI-n hydrogels exhibited significantly greater antimicrobial activity than did PFI 6.0, with the antimicrobial effect increasing in proportion to the DMY concentration. DPFI-3 exhibited the strongest antibacterial performance, effectively killing both MRSA and *E. coli*. Bacterial live/dead staining was performed to further validate these results. Figure 4c and d show that bacteria treated with blank PFI 6.0 exhibited golden yellow fluorescence, indicating partial bacterial death. In contrast, DPFI hydrogels—particularly DPFI-3—induced a significant increase in red fluorescence, indicating near-complete bacterial death, which corroborated the plate counting results. Additionally, SEM was used to observe morphological changes in MRSA and *E. coli* after hydrogel treatment. As shown in Figure 4e, untreated bacteria maintained smooth surfaces and intact structures. Following PFI 6.0 treatment, bacteria displayed minor wrinkling and shrinkage. DPFI-1 treatment caused more pronounced wrinkling and structural damage, whereas DPFI-2 and DPFI-3 treatments resulted in the complete destruction of MRSA and extensive crumpling and flattening of *E. coli*, with both bacterial strains being fully eradicated.

### *In vitro* antioxidant activity of DPFI

**Figure 5:**
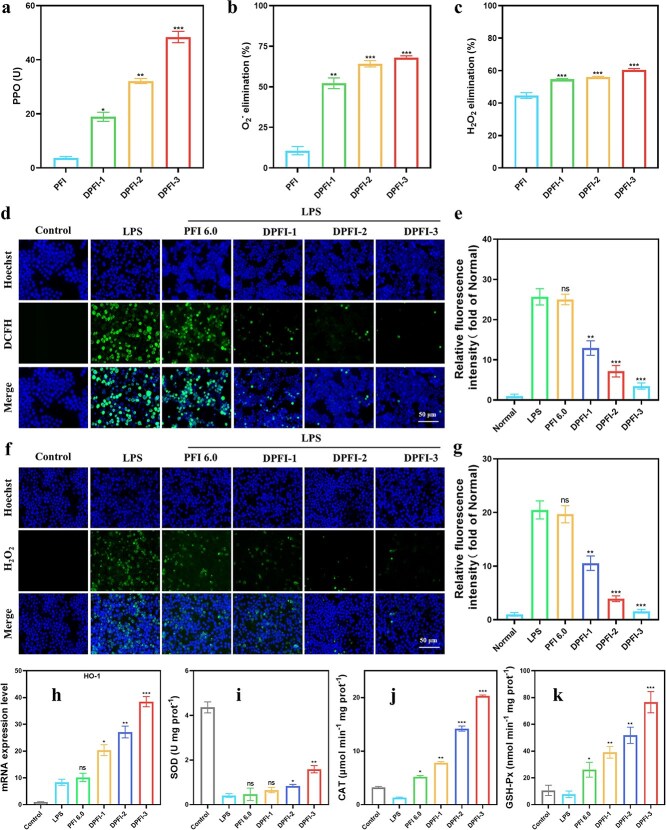
Antioxidant properties of DPFI-n hydrogels. (**a**–**c**) PPO value, superoxide anion radical (•O_2_^−^) and H_2_O_2_ scavenging efficiency. (**d** and **e**) Intracellular ROS scavenging capacity of DPFI (scale bar: 50 μm). (**f** and **g**) Intracellular H_2_O_2_ scavenging capacity of DPFI (scale bar: 50 μm). (**h**-**k**) Expression levels of HO-1 and SOD, CAT, and GSH-Px in DPFI treated activated macrophages. ^*^P <0.05, ^**^P <0.01, ^***^P <0.001, ns: no significance. In figures a–c, comparisons are made between the PFI group and other groups, while in figures e, g, h–k, comparisons are made between the LPS group and other groups. LPS: Lipopolysaccharide; DCFH: 2′,7′-dichlorodihydrofluorescein

The antioxidant properties of the DPFI hydrogels were initially evaluated using PPO, SOD, and CAT assays. The results indicated that higher drug loading resulted in increased antioxidant effects, suggesting efficient drug incorporation and functional activity (Figure 5a–c). Since ROS play a critical role in promoting wound inflammation, we induced oxidative stress in RAW 264.7 cells with 3 μg/ml LPS. These cells were subsequently treated with PFI and DPFI-n extracts. The ROS and H_2_O_2_ levels in the treated cells were measured using specific fluorescent indicators and an inverted fluorescence microscope. As shown in Figure 5d and e, LPS induction significantly increased ROS production in macrophages, with a fluorescence intensity ~24 times higher than that in the control group. Treatment with the therapeutic materials reduced ROS levels, with the DPFI-3 group showing the greatest reduction that was only ~0.13 times the fluorescence intensity of the LPS-treated group. Similarly, the highest H_2_O_2_ levels were observed in the LPS group and were ~20 times higher than those in the control group, while the DPFI-3 treatment reduced H_2_O_2_ levels to ~0.08 times those of the LPS group (Figure 5f and g). We further assessed the antioxidant activity of DPFI-n by measuring the expression of HO-1 and the activities of the enzymes SOD, CAT, and GSH-Px (Figure 5h–k). The DPFI-n hydrogels displayed a clear trend toward increased antioxidant activity with increasing DMY content, as evidenced by the significantly increased expression levels and enzymatic activities of these key antioxidant enzymes. Notably, although SOD activity in the DPFI-3 group did not significantly exceed that of the control group, SOD consumption was effectively reduced, maintaining a level of 1.56 (U mg protein^−1^).

### Immunomodulatory and anti-inflammatory efficacy of DPFI

**Figure 6:**
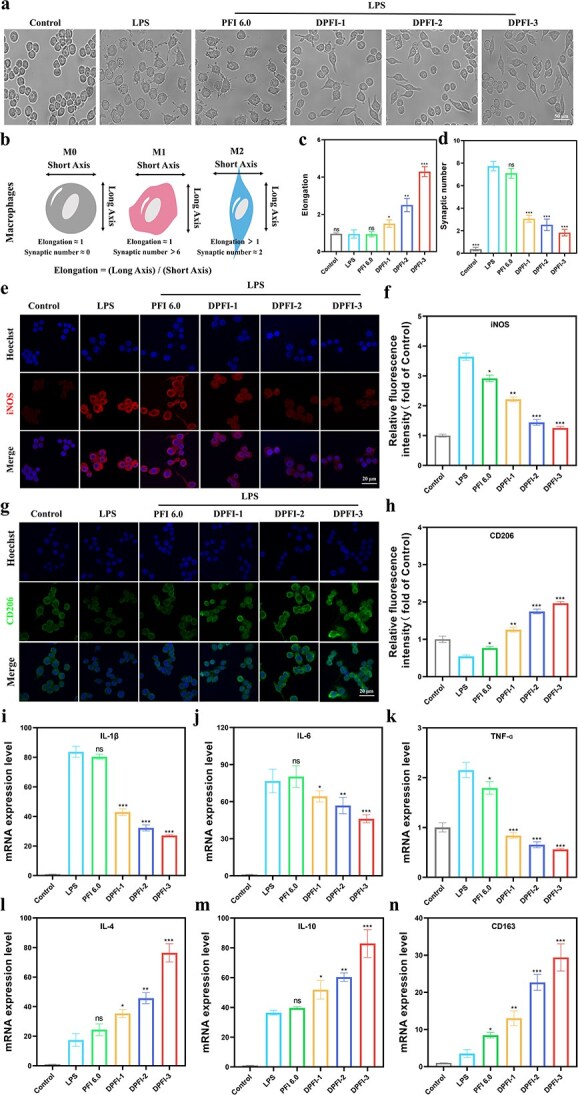
Immunomodulatory and anti-inflammatory properties of DPFI-n hydrogels. (**a**) Morphological changes in LPS-induced activated RAW 264.7 macrophages treated with DPFI-n hydrogel, demonstrating M1 to M2 polarization (scale bar: 50 μm). (**b**) Schematic representation of macrophages exhibiting different phenotypes. (**c** and **d**) Cell elongation and synaptic number changes of macrophages after different treatments. (**e** and **f**) Immunofluorescence imaging of iNOS-positive macrophages, with corresponding fluorescence intensity, highlighting reduced M1 markers following DPFI treatment (scale bar: 20 μm). (**g** and **h**) Immunofluorescence imaging of CD206-positive macrophages, along with corresponding fluorescence intensity, demonstrating increased M2 marker expression (scale bar: 20 μm). (**i**–**k**) PCR analysis of pro-inflammatory cytokine expression levels (IL-6, IL-1β, TNF-α) in macrophages after DPFI-n hydrogel treatment. (**l**–**n**) Expression levels of anti-inflammatory cytokines IL-4, IL-10, CD163 in macrophages after DPFI-n hydrogel treatment. ^*^P <0.05, ^**^P <0.01, ^***^P <0.001, ns: no significance. Comparisons are made between the LPS group and other groups

We investigated the immunomodulatory effects of DPFI on macrophage polarization by analyzing morphological changes in RAW 264.7 cells after treatment with the different materials. As shown in Figure 6a–d, control macrophages exhibited a rounded, clustered morphology, whereas LPS-stimulated cells displayed the typical dendritic features characteristic of M1 macrophages. After treatment with DPFI, the cells adopted elongated shapes with longer pseudopods, which are indicative of the M2 phenotype. These morphological changes became more pronounced with higher DMY concentrations. Further validation of macrophage polarization was performed using immunofluorescence staining for iNOS and CD206, which distinguish M1 and M2 macrophages, respectively. Figure 6e and f show that the iNOS fluorescence intensity was high in the LPS and PFI groups but decreased as the DMY concentration increased, with DPFI-3 showing only 0.35 times the intensity observed in the LPS group. Conversely, CD206 fluorescence, indicative of M2 macrophages, was weakest in the LPS and PFI 6.0 groups but significantly increased with increasing DMY concentration, with the DPFI-3 group showing 3.62 times greater fluorescence intensity than the LPS group (Figure 6g and h). The PCR analysis further confirmed the anti-inflammatory effects of DPFI. Figure 6i–k show that the expression levels of the proinflammatory cytokines IL-6, IL-1β, and TNF-α were significantly reduced in the DPFI group compared with the control and PFI 6.0 groups. In contrast, the levels of the anti-inflammatory markers IL-10, IL-4, and CD163 were significantly increased in the DPFI-3 group (Figure 6l–n).

### DPFI hydrogels facilitate cellular proliferation, directed migration, and angiogenesis *in vitro*

**Figure 7:**
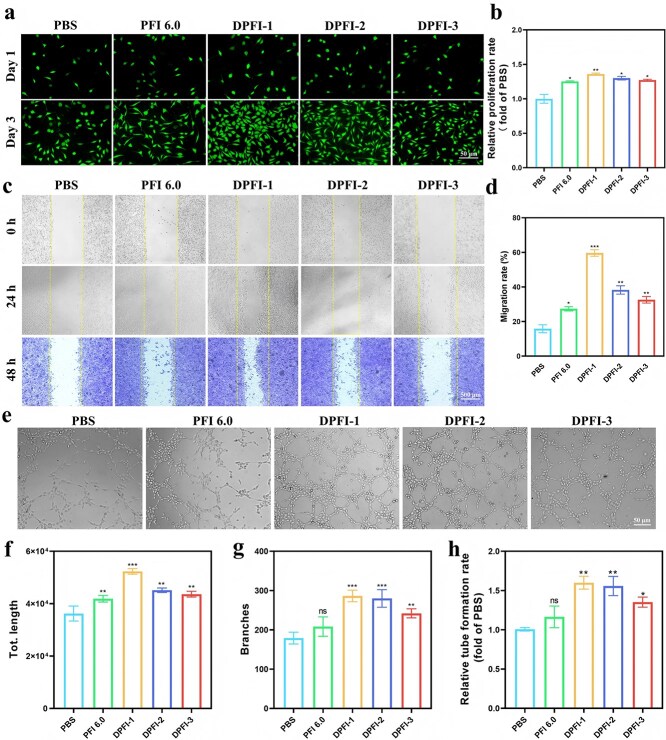
DPFI hydrogels promote cell proliferation, migration, and angiogenesis. (**a**) Cell proliferation assay assessing L929 cell growth following co-culture with DPFI hydrogels for 1 and 3 days (scale bar: 50 μm). (**b**) Quantification of cell proliferation rate after 3 days of co-incubation. (**c**) Scratch assay assessing the migration ability of L929 cells co-cultured with DPFI hydrogels (scale bar: 500 μm). (**d**) Quantification of scratch healing rate. (**e**) HUVEC tube formation assay after treatment with hydrogel extracts, illustrating effects on angiogenesis (scale bar: 50 μm). (**f**) Quantification of total tube length, (**g**) number of branches, and (h) relative tube formation rate. ^*^P <0.05, ^**^P <0.01, ^***^P <.001, ns: no significance. Comparisons are made between the PBS group and other groups

We conducted cell proliferation and scratch wound healing assays using L929 fibroblasts to assess the effects of the DPFI hydrogel on fibroblast proliferation and migration. As illustrated in Figure 7a and b, the proliferation of cells treated with DPFI-1 closely mirrored the cytocompatibility results observed previously for DPFI-n. On Day 1 of coincubation, the cell counts were similar across all the groups. However, by Day 3, the drug-loaded DPFI-treated groups presented significantly greater cell numbers than did the PBS and PFI 6.0 groups. Notably, the DPFI-1 group presented the highest proliferation rate, while the higher drug concentrations in DPFI-2 and DPFI-3 seemed to slow the proliferation rate.

The results of the scratch wound healing assay indicated that DPFI treatment accelerated fibroblast migration (Figure 7c and d). Notably, the healing area achieved by DPFI-3 was 59.6%, representing increases of 43.8% and 32.3% compared with those of the PBS group and PFI 6.0, respectively.

We conducted *in vitro* assays with HUVECs to investigate the proangiogenic potential of the DPFI hydrogels. As shown in Figure 7e–h, DPFI hydrogels markedly promoted capillary-like tube formation, with the DPFI-1 group showing the most pronounced effects in terms of the node count, branch length, and overall tube formation rate. However, a negative correlation was observed between the DMY concentration and angiogenic capacity, as DPFI-2 and DPFI-3 were associated with reductions in these angiogenic parameters. These findings suggest that optimal DMY loading is essential for maximizing vascularization. Specifically, HUVECs treated with DPFI-1 presented a 1.44-fold increase in vessel length and a 1.60-fold increase in branch point formation compared with those treated with the PBS control.

### Therapeutic efficacy of DPFI hydrogels in wound closure and tissue repair

**Figure 8:**
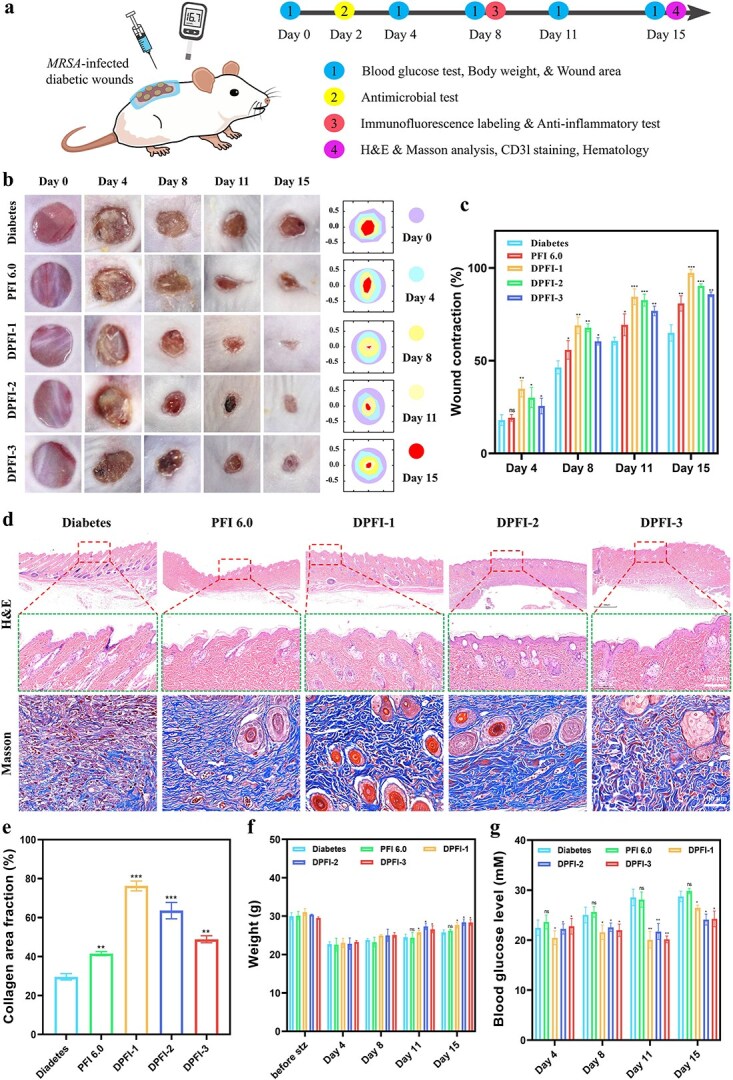
*In vivo* evaluation of the wound healing efficacy of DPFI hydrogels. (**a**) Schematic diagram illustrating the experimental protocol. (**b**) Representative images depicting the dynamic process of wound closure across different treatment groups >15 days of treatment. (**c**) Wound closure rates across different treatment groups on days 4, 8, 11, and 15. (**d**) H&E and Masson’s staining of tissue sections at 15 days post-treatment (scale bar: 100 μm). (**e**) Quantification of collagen content in the wound tissue. (**f** and **g**) Changes in body weight and blood glucose levels on days 4, 8, 11 and 15. ^*^P <0.05, ^**^P <0.01, ^***^P <0.001, ns: no significance. Comparisons are made between the diabetes group and other groups

An *in vivo* model was established using MRSA-infected diabetic wounds in streptozotocin-induced diabetic mice to evaluate the therapeutic potential of DPFI hydrogels for healing diabetic wounds (Figure 8a). Treatment with DPFI hydrogels significantly accelerated wound healing, outperforming both the control and blank PFI 6.0-treated groups. The macroscopic evaluation of wound closure revealed a substantial reduction in the wound area by Day 11 in the DPFI-treated groups (DPFI-1, DPFI-2, and DPFI-3), with DPFI-1 achieving the highest closure rate (97.3% by Day 15) (Figure 8b and c). Notably, DPFI-1 treatment resulted in a nearly completely healed wound bed, whereas the DPFI-2 and DPFI-3 groups presented 90.37% and 85.79% healing rates, respectively. These results were considerably better than those observed in the control (64.94%) and PFI 6.0 (80.89%) groups. These findings clearly indicate that drug-loaded DPFI hydrogels promote more effective healing of MRSA-infected diabetic wounds than nondrug-loaded controls.

Histological analyses further validated the tissue repair ability of the DPFI hydrogels. Figure 8d and e show that wounds treated with DPFI hydrogels presented minimal inflammatory cell infiltration and a marked presence of neoplastic granulation tissue, with well-formed follicular structures. Masson’s trichrome staining revealed significantly greater collagen deposition and mature fibrous tissue in the DPFI-treated groups than in the blank and PFI 6.0 groups. Among all the groups, DPFI-1 resulted in the most pronounced degree of collagen fiber formation, covering 76.25% of the wound area, highlighting its superior ability to promote extracellular matrix remodeling and facilitate wound regeneration. These histopathological findings further emphasize the efficacy of DPFI hydrogels in modulating both the tissue repair and remodeling phases of the wound healing process.

In addition to affecting local tissue repair, DPFI treatment had a notable effect on the overall physiological recovery of diabetic mice. As shown in Figure 8f, diabetic mice in the hydrogel-treated group began to gradually regain body weight starting on Day 4, with the DPFI-n groups displaying significantly better weight recovery than the diabetes and PFI 6.0 groups. This improvement in body weight suggests that DPFI hydrogels promote systemic recovery by alleviating the pathological conditions associated with diabetes and chronic wounds.

Given the complex relationship between hyperglycemia and impaired wound healing, the ability of DPFI hydrogels to modulate blood glucose levels was a key focus of this study. As illustrated in Figure 8g, mice that were not treated with DMY-containing hydrogels exhibited a gradual increase in blood glucose levels, whereas those treated with DMY-containing hydrogels experienced a significant reduction in blood glucose levels after 11 days, especially mice in the DPFI-3 group. Although drug effectiveness may decrease as wounds heal, by Day 15, blood glucose levels in the DPFI-treated groups remained well controlled and were significantly lower than those in the diabetes and PFI 6.0 groups.

### *In vivo* antimicrobial, anti-inflammatory, and angiogenic effects of DPFI hydrogels

**Figure 9:**
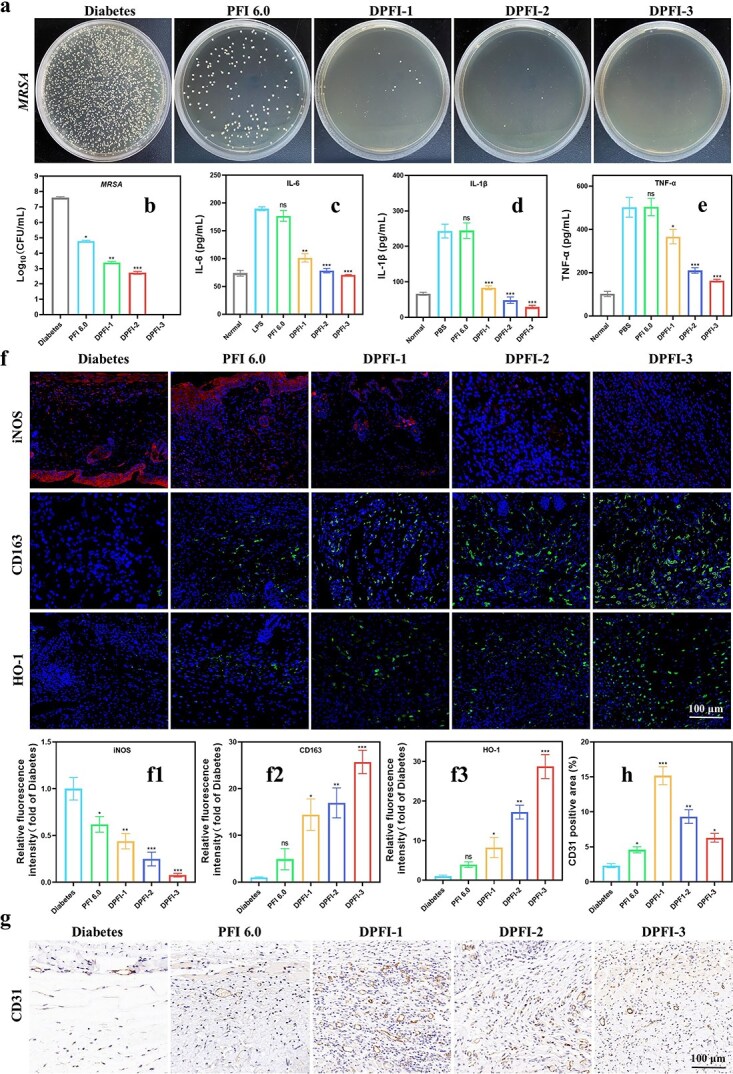
*In vivo* assessment of antimicrobial, anti-inflammatory, and angiogenic properties of DPFI-n hydrogels. (**a**) Representative images of residual bacterial colonies after 2 days of treatment. (**b**) Quantitative bacterial counts across different treatment groups. (**c**–**e**) Quantification of IL-6, IL-1β, and TNF-α expression levels in mice on day 8 across different groups. (**f**) Immunofluorescence staining of iNOS, CD163, and HO-1 with corresponding fluorescence intensities in wound tissues from different treatment groups on day 8 (scale bar: 100 μm). (**g** and **h**) Immunofluorescence staining of CD31-positive areas and corresponding fluorescence intensities in wound tissues from different treatment groups on day 15 (scale bar: 100 μm). ^*^P <0.05, ^**^P <0.01, ^***^P <0.001, ns: no significance. All comparisons are made between the diabetes group and other groups

We elucidated the mechanisms underlying the multifunctional efficacy of DPFI hydrogels in managing MRSA-infected diabetic wounds by evaluating their antimicrobial, anti-inflammatory, antioxidant, and biocompatibility properties *in vivo*. We first investigated the antibacterial properties of the hydrogels by collecting bacterial fluids from the wounds after 2 days of treatment for plate culture experiments. As shown in Figure 9a and b, the antimicrobial efficacy of the hydrogels improved significantly with increasing drug loading. Notably, the DPFI-n hydrogels nearly completely eliminated bacteria, with the DPFI-3 group demonstrating particularly excellent antimicrobial activity. These results strongly suggest that the combination of PEI, a cationic polymer with intrinsic antimicrobial properties, and DMY results in a synergistic effect.

We performed ELISAs and immunofluorescence staining to further investigate the impact of DPFI hydrogels on inflammatory modulation and angiogenesis. Blood samples were collected from the mice on Day 8, and a quantitative analysis of the levels of proinflammatory cytokines, including IL-6, IL-1β, and TNF-α, was conducted using ELISA kits. The results, shown in Figure 9c–e, revealed a decreasing trend in the levels of inflammatory factors as drug loading increased, confirming the significant anti-inflammatory effect of the DPFI hydrogels. Immunofluorescence staining was performed on wound tissues collected on Day 8 to assess the expression of iNOS, CD163, and HO-1. As shown in Figure 9f, tissue samples treated with DPFI exhibited prominent green fluorescence for both CD163 and HO-1 under the fluorescence microscope. The fluorescence intensity increased with increasing drug loading, with tissues samples from animals treated with DPFI-3 showing fluorescence intensities for CD163 and HO-1 that were 25.7 and 28.7 times higher than those observed in the diabetes group, respectively. Conversely, iNOS staining in the PBS and PFI groups revealed strong red fluorescence, indicative of severe inflammation. The fluorescence intensity of iNOS in the drug-loaded hydrogel groups decreased with increasing drug loading, with tissue samples from animals treated with DPFI-3 exhibiting an intensity that was only 0.076 times the intensity of the diabetes group. Additionally, CD31 staining was performed to label regenerating capillaries, as shown in Figure 9g and h. Consistent with the *in vitro* angiogenesis experiments, the DPFI-1-treated group presented a significantly greater number of microvessels than the other experimental groups, highlighting superior capillary regeneration. Our experimental results suggest that during the inflammatory phase, the 3% DMY-loaded hydrogel produces the most effective therapeutic outcomes. In contrast, during the proliferative phase, 1% DMY treatment has significant therapeutic effects.

We conducted a series of *in vivo* biocompatibility assays to evaluate the safety profile of DPFI hydrogels for potential clinical applications. The hemolytic properties of the hydrogels were first assessed. As shown in Figure S13a–c, erythrocytes exposed to ultrapure water (positive control) lysed, resulting in a red solution, whereas the solution of red blood cells exposed to the PBS solution (negative control) remained clear and transparent. Our analysis of various ratios of PFI hydrogels and DPFI hydrogels with different drug loadings revealed that their hemolysis rates were consistently <5%, in compliance with International Organization for Standardization (ISO) standards. This finding indicates that the materials are compatible with blood. Additionally, a whole-skin wound infection model was used to assess the biosafety of the hydrogels. After 15 days of treatment with the DPFI hydrogel, major organs and plasma were collected from the rats for histological and biochemical evaluation. Routine blood tests revealed that key hematological parameters, including the red blood cell (RBC) count, white blood cell (WBC) count, and hemoglobin (HGB) level, remained within normal ranges (Figure S14a–c). Furthermore, H&E staining of the major organs (e.g. heart, liver, spleen, lungs, and kidneys) collected after 15 days of treatment revealed no significant abnormalities (Figure S15).

## Discussion

This study presents the development of DPFI, a multifunctional hydrogel system engineered for efficient drug delivery and accelerated wound healing. The successful encapsulation of DMY within PF127-CHO micelles was confirmed by the increased micelle size and increased drug solubility, ensuring improved bioavailability. The Schiff base formation between the aldehyde groups in PF127-CHO and the amino groups in PEI enabled rapid gelation. Notably, the increase in the aldehyde content significantly strengthened the hydrogel network structure, thereby enhancing its mechanical properties. DPFI hydrogels exhibited pronounced shear-thinning behavior, enabling easy injection and conformal adaptation to irregular wound geometries. The strong adhesion to biological tissues is attributed to the inherent adhesive properties of PEI and the chemical bonds formed through the Schiff base reaction between the aldehyde groups in F127-CHO and the amino groups in the tissues. This dual mechanism ensures intimate contact between the hydrogel and the wound site while providing a protective barrier. Furthermore, the hydrogels exhibited self-healing capabilities, allowing structural recovery after damage and reducing the need for frequent reapplications.

The degradation rate of hydrogels is inversely correlated with their density, with higher-density hydrogel structures being more stable and able to maintain their integrity for a longer duration. Drug release profiles showed a controlled pattern governed by both the hydrogel composition and the DMY concentration, supporting sustained therapeutic action. While DPFI hydrogels displayed favorable cytocompatibility, elevated concentrations of PF127-CHO and DMY slightly attenuated cell proliferation, emphasizing the need for optimized formulation parameters. Overall, DPFI hydrogels present a promising combination of mechanical robustness, tissue adhesion, controlled drug release, and biocompatibility.

The hydrogels exhibited strong antimicrobial efficacy. The combined action of PEI and DMY resulted in superior antibacterial performance, with DPFI-3 exerting the strongest effect. The cationic nature of PEI disrupted bacterial cell membranes, whereas DMY further induced oxidative stress and inhibited bacterial nucleic acid synthesis, thereby increasing bactericidal activity. Live/dead staining and SEM imaging confirmed extensive bacterial damage, with higher DMY concentrations achieving near-complete eradication. The antioxidant activity of the DPFI hydrogels further enhanced their wound-healing potential by mitigating oxidative stress. Higher DMY loading improved the ROS-scavenging efficiency, with DPFI-3 resulting in the greatest reductions in the ROS and H_2_O_2_ levels. This effect was supported by the increased expression and activity of key antioxidant enzymes, including HO-1, SOD, CAT, and GSH-Px. Notably, although the SOD activity in the DPFI-3 group did not exceed that of the control group, SOD consumption was effectively reduced. These findings indicate the dual therapeutic role of DPFI hydrogels, effectively controlling inflammation while fostering a favorable wound microenvironment.

In addition to their antimicrobial and antioxidant properties, DPFI hydrogels exerted significant immunomodulatory and anti-inflammatory effects, which are essential for wound healing. M1 macrophages, which predominate in chronic wounds, contribute to persistent inflammation, whereas M2 macrophages facilitate tissue repair. Notably, DMY promotes the M1-to-M2 macrophage polarization, a pivotal process in resolving inflammation. Immunofluorescence staining and PCR analysis confirmed that DPFI treatment significantly downregulated the expression of proinflammatory cytokines (IL-6, IL-1β, and TNF-α) while increasing the expression of anti-inflammatory cytokines (IL-10, IL-4, and CD163), with DPFI-3 having the most pronounced regulatory effect. When applied *in vivo*, this immunomodulatory effect can reduce the chronic inflammatory state commonly observed in diabetic wounds, creating a more favorable environment for tissue repair and regeneration. The mechanism underlying this regulatory effect is closely linked to the powerful ROS scavenging ability of DMY. Inflammatory stimuli lead to the continuous secretion of elevated ROS levels by activated macrophages, which promotes polarization toward the M1 phenotype and drives the release of proinflammatory cytokines, exacerbating inflammation [45, 46]. By effectively scavenging excess ROS, DMY facilitates the shift to the M2 phenotype, thus reducing inflammation and supporting the wound healing process.

The hydrogels also increased fibroblast proliferation, migration, and angiogenesis—critical processes for effective wound healing. DPFI-1 treatment significantly promoted fibroblast proliferation; however, the higher drug concentrations in DPFI-2 and DPFI-3 led to a slight reduction in proliferation, potentially due to saturation effects or dose-dependent cytotoxicity. Similarly, scratch wound healing assays indicated accelerated fibroblast migration with DPFI treatment, although elevated DMY concentrations may have altered cellular adhesion, suggesting a concentration-dependent response. The proangiogenic potential of the DPFI hydrogels was also evident, with DPFI-1 exerting the most pronounced effect on capillary-like tube formation. Conversely, increasing DMY concentrations correlated with a decrease in the angiogenic capacity, underscoring the necessity for optimized drug loading to maximize vascularization. These findings highlight the importance of achieving an optimal DMY concentration to fully harness the therapeutic potential of DPFI hydrogels.

*In vivo* studies further validated the therapeutic efficacy of DPFI hydrogels in diabetic wound treatment. In a diabetic murine model, wounds treated with DPFI exhibited significantly accelerated healing compared with nondrug-loaded controls, with DPFI-1 resulting in the highest wound closure rate. This enhanced healing response is likely due to the combined effects of antimicrobial activity, inflammation modulation, and improved vascularization provided by the hydrogel system. In addition to their local effects, DPFI hydrogels also influenced systemic recovery, as indicated by the improved body weight restoration and glycemic control. The histological analysis confirmed enhanced tissue repair, as evidenced by minimal inflammatory cell infiltration, increased granulation tissue formation, and substantial collagen deposition, particularly in DPFI-1-treated wounds. These observations suggest that DPFI hydrogels actively support both the inflammatory resolution and tissue remodeling phases of wound healing. Moreover, the body weight of the treated animals improved, indicating a broader therapeutic effect on alleviating diabetes-associated pathological conditions. Importantly, DMY-containing hydrogels significantly reduced blood glucose levels, with DPFI-3 displaying the most pronounced hypoglycemic effect. This glucose-lowering activity is likely due to the ability of DMY to mitigate oxidative stress, protect pancreatic β-cells, and increase insulin sensitivity [47].

DPFI hydrogels exhibit combined antimicrobial, anti-inflammatory, and proangiogenic capabilities, effectively improving the wound microenvironment and promoting the healing process. The experimental data revealed that the levels of inflammatory factors (including IL-6, IL-1β, and TNF-α) in the drug-loaded groups were significantly decreased, the iNOS fluorescence intensity decreased and the CD163 and HO-1 fluorescence intensities were significantly increased, confirming its mechanism of action in reducing inflammatory responses and promoting the transition of wounds to the proliferative phase [48]. CD31 staining further demonstrated that microvascular formation was significantly increased in DPFI-1-treated wounds, reflecting an excellent vascular regeneration capacity. DPFI hydrogels exhibit excellent biocompatibility and have no significant adverse effects on major organs. The optimization of drug loading is a key factor in achieving therapeutic efficacy: higher concentrations of DMY are particularly effective at reducing inflammation, whereas lower concentrations are more favorable for angiogenesis. Additionally, future research could consider integrating fluorescent probe-functionalized materials to monitor ROS levels and inflammation resolution in real time, which holds significant value for *in vivo* studies of chronic diabetic wounds [49].

In summary, DPFI hydrogels effectively modulate key wound healing processes, including antimicrobial defense, inflammation resolution, and neovascularization. The observed acceleration of wound contraction, hyperglycemic modulation, and enhanced tissue remodeling suggest that DPFI is a bio-responsive therapeutic platform. These multifunctional properties position DPFI as a promising strategy for treating chronic diabetic wounds, warranting further clinical investigation.

## Conclusions

This study presents a multifunctional hydrogel dressing (DPFI) loaded with DMY designed to provide sequential therapeutic actions tailored to the complex pathophysiology of chronic diabetic wounds. DPFI combines antimicrobial, antioxidant, anti-inflammatory, pro-vascularization, and glycemic-regulating properties, making it an innovative solution for wound care. It exhibits exceptional bioadhesion, injectability, and self-repair capabilities. The experimental results confirm that the hydrogel effectively inhibits bacterial growth, creating a favorable environment for healing. It also possesses potent antioxidant activity by scavenging ROS and promoting antioxidant enzyme production, which aids in shifting macrophages from the M1 to M2 phenotype and reduces the inflammatory response. Additionally, the ability of the hydrogel to regulate glucose modulates the diabetic wound environment, improving fibroblast proliferation, endothelial cell migration, and neovascularization and ultimately accelerating tissue repair and wound closure. These multifunctional properties highlight the significant potential of DPFI as an advanced hydrogel-based platform for managing chronic diabetic wounds. With its comprehensive therapeutic approach, the DPFI hydrogel represents a promising candidate for future clinical applications and a versatile strategy in diabetic wound treatment.

## Supplementary Material

Figure_S1_tkaf024

Figure_S2_tkaf024

Figure_S3_tkaf024

Figure_S4_tkaf024

Figure_S5_tkaf024

Figure_S6_tkaf024

Figure_S7_tkaf024

Figure_S8_tkaf024

Figure_S9_tkaf024

Figure_S10_tkaf024

Figure_S11_tkaf024

Figure_S12_tkaf024

Figure_S13_tkaf024

Figure_S14_tkaf024

Figure_S15_tkaf024

Table_S1_tkaf024

Table_S2_tkaf024

Table_S3_tkaf024

## Data Availability

The data that support the findings of this study are available from the corresponding authors upon reasonable request.
