# Supplementary figures and images for: A multifunctional dihydromyricetin-loaded hydrogel for the sequential modulation of diabetic wound healing and glycemic control
Source: Burns Trauma. 2025 Mar 19;13:tkaf024. doi: 10.1093/burnst/tkaf024 (PMC12315528; doi:10.1093/burnst/tkaf024)

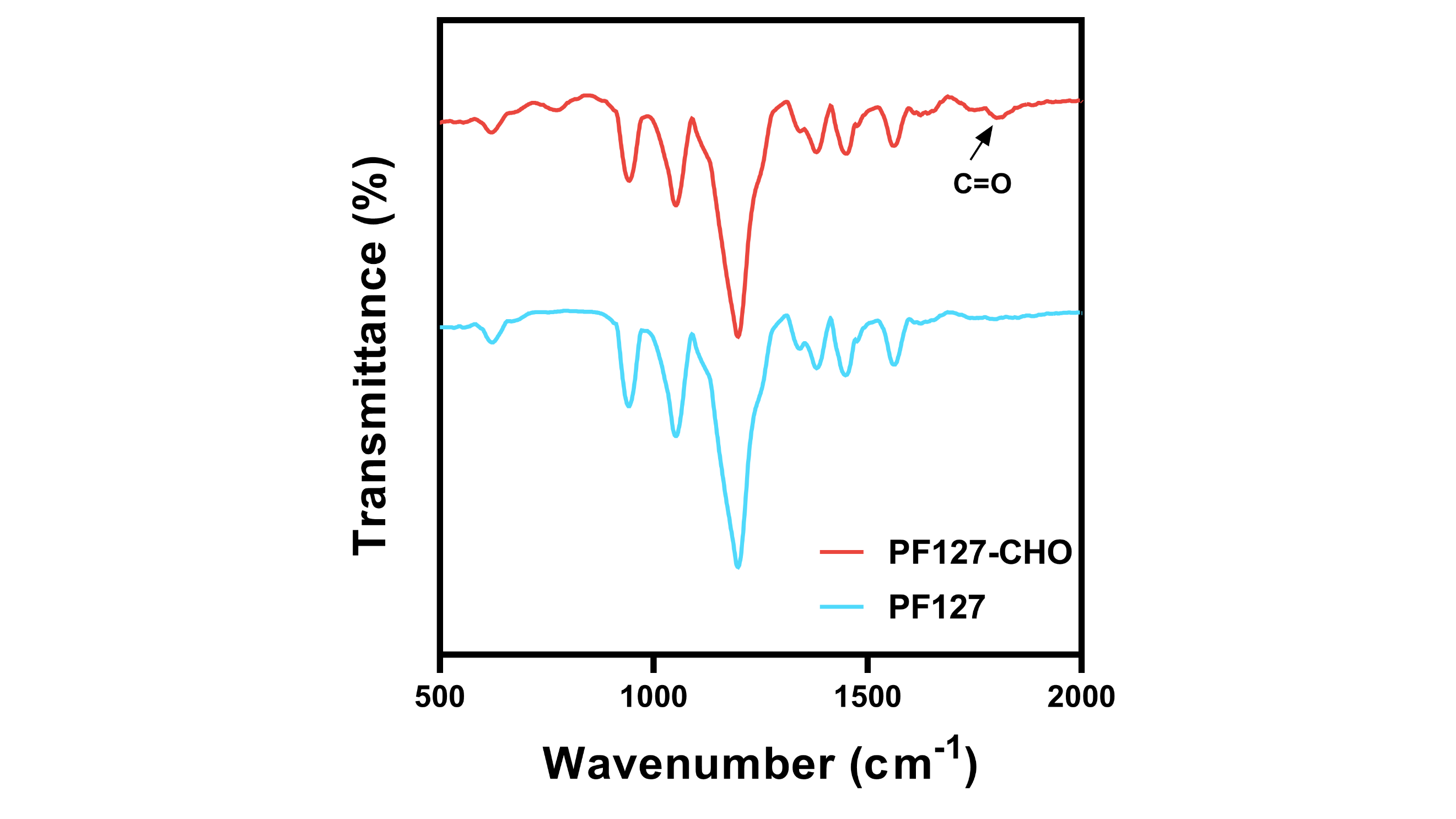


**Figure S1.** FT-IR spectra of PF127 and PF127-CHO.

Supplement: Figure_S1_tkaf024 [file figure_s1_tkaf024.docx]

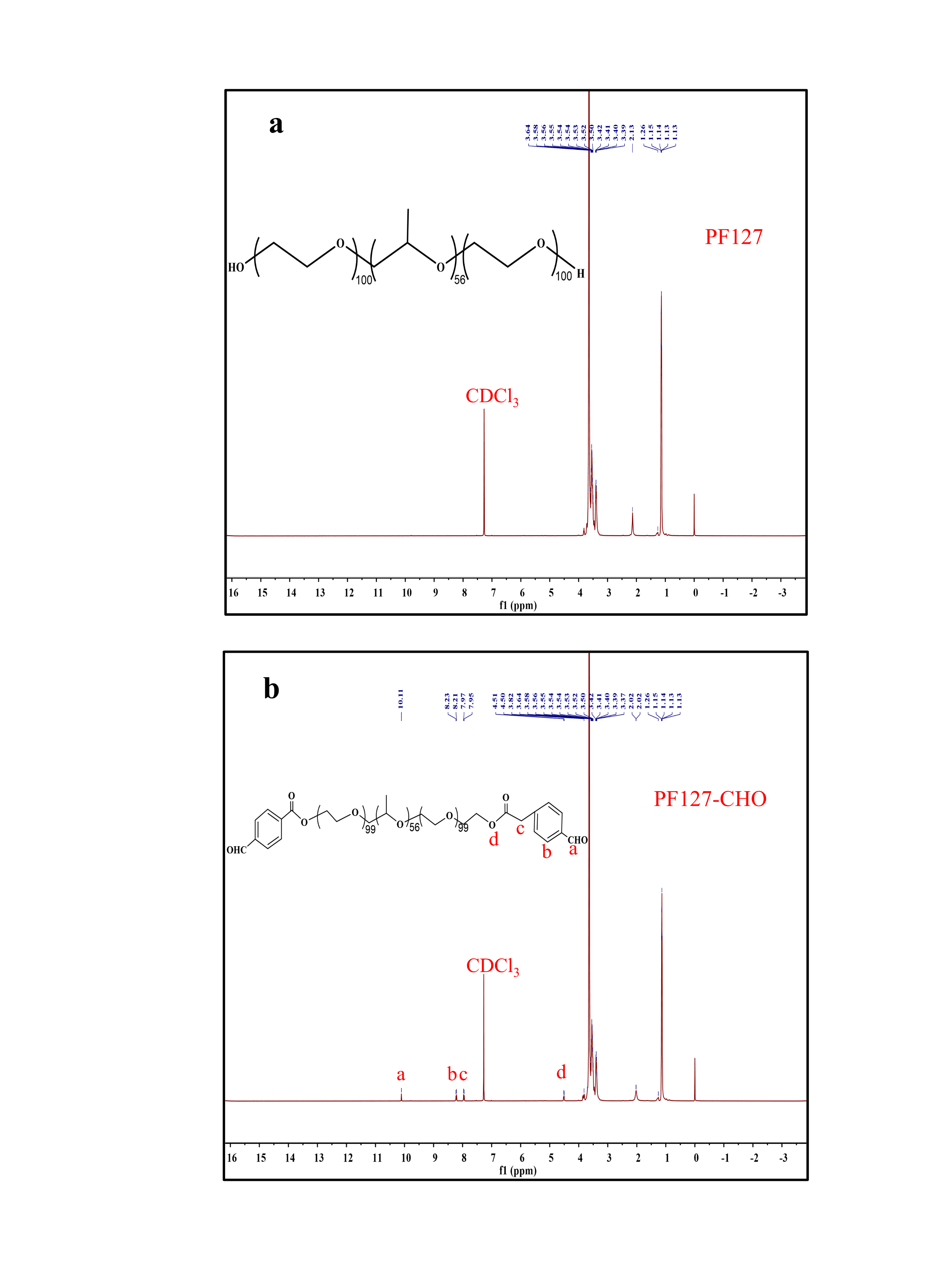


**Figure S2.** 1H NMR spectra of (a) F127 and (b) F127-CHO.

Supplement: Figure_S2_tkaf024 [file figure_s2_tkaf024.docx]

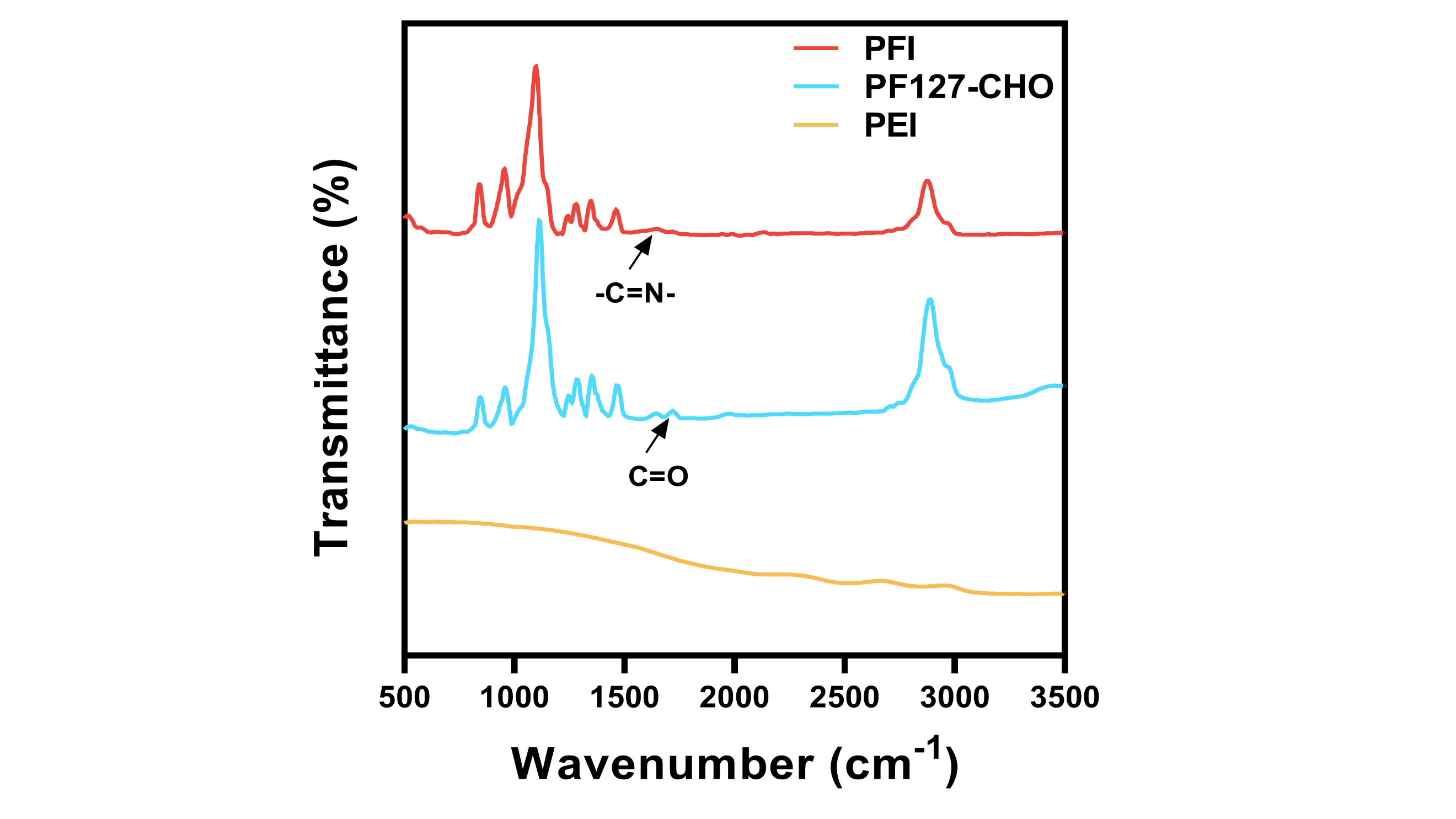


**Figure S3.** FT-IR spectra of PF127-CHO, PEI and PFI.

Supplement: Figure_S3_tkaf024 [file figure_s3_tkaf024.docx]

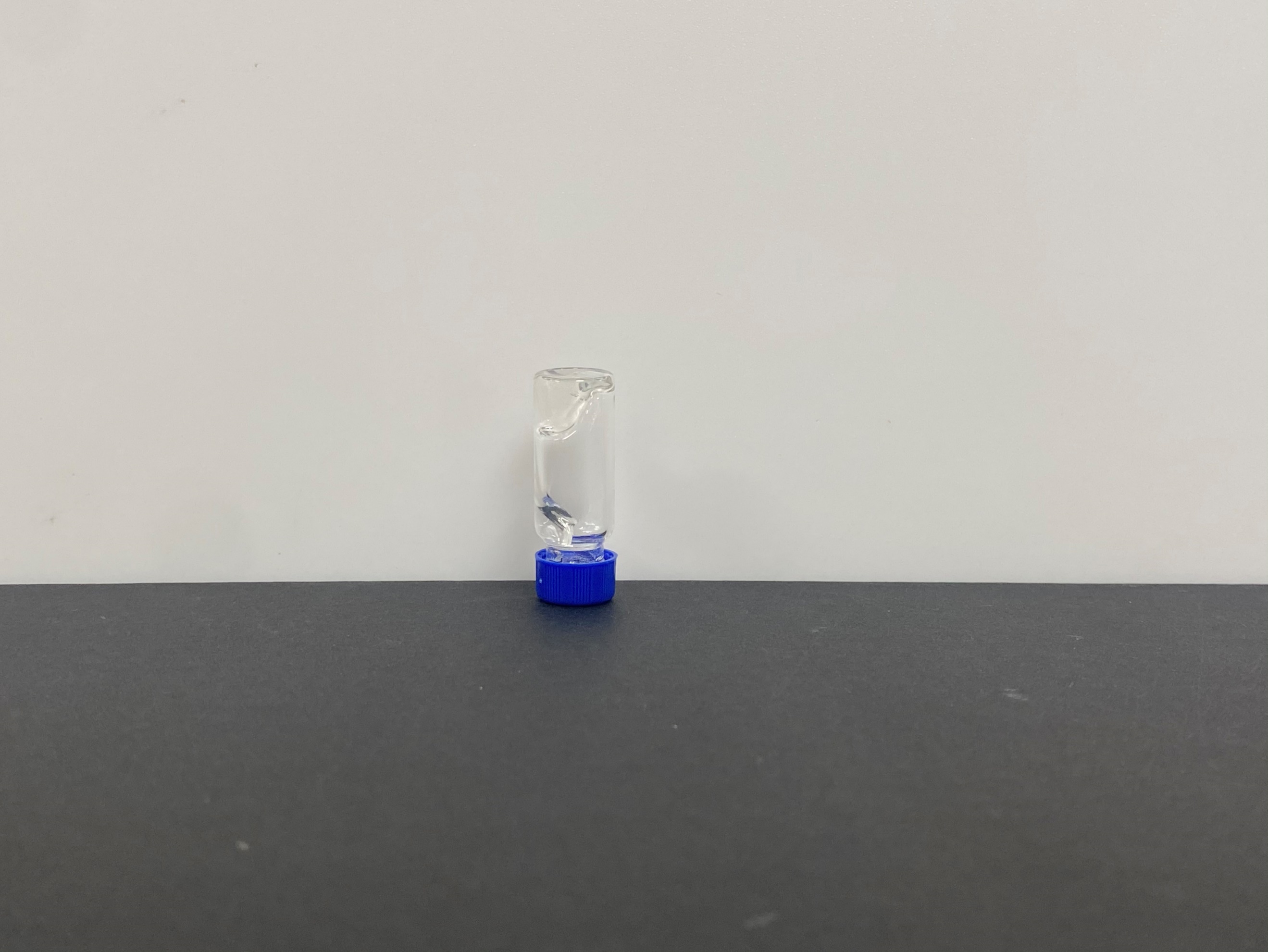

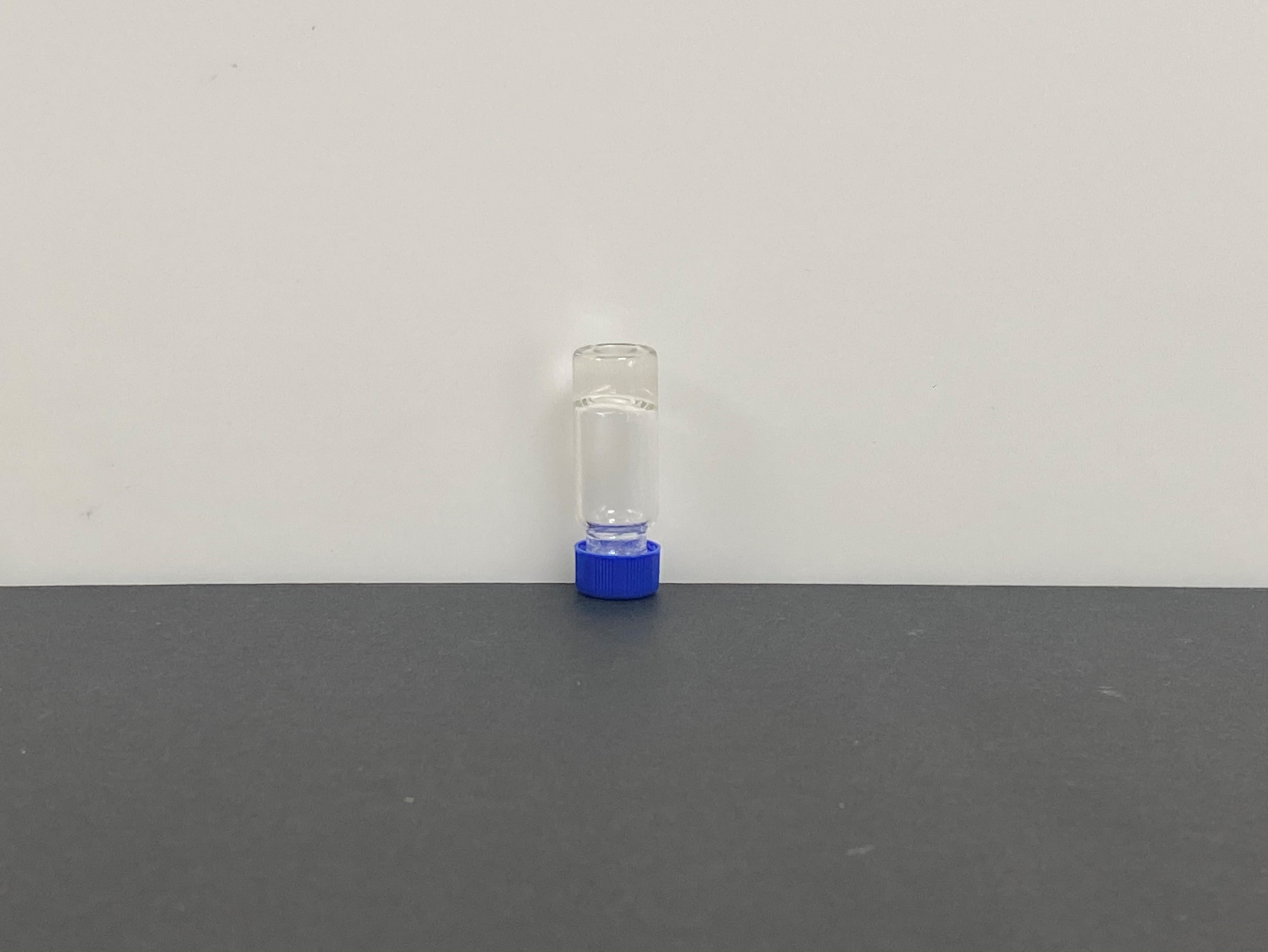

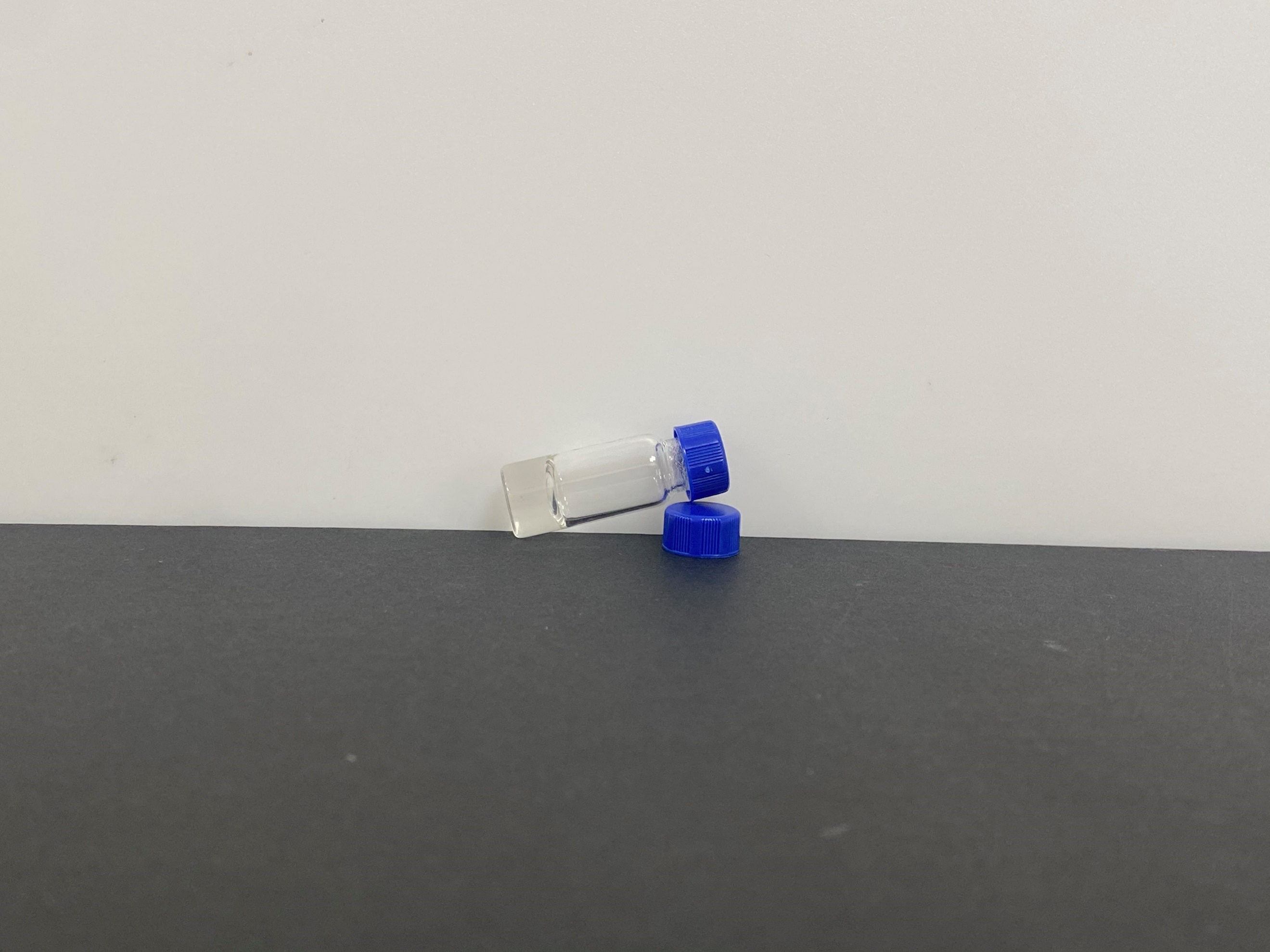

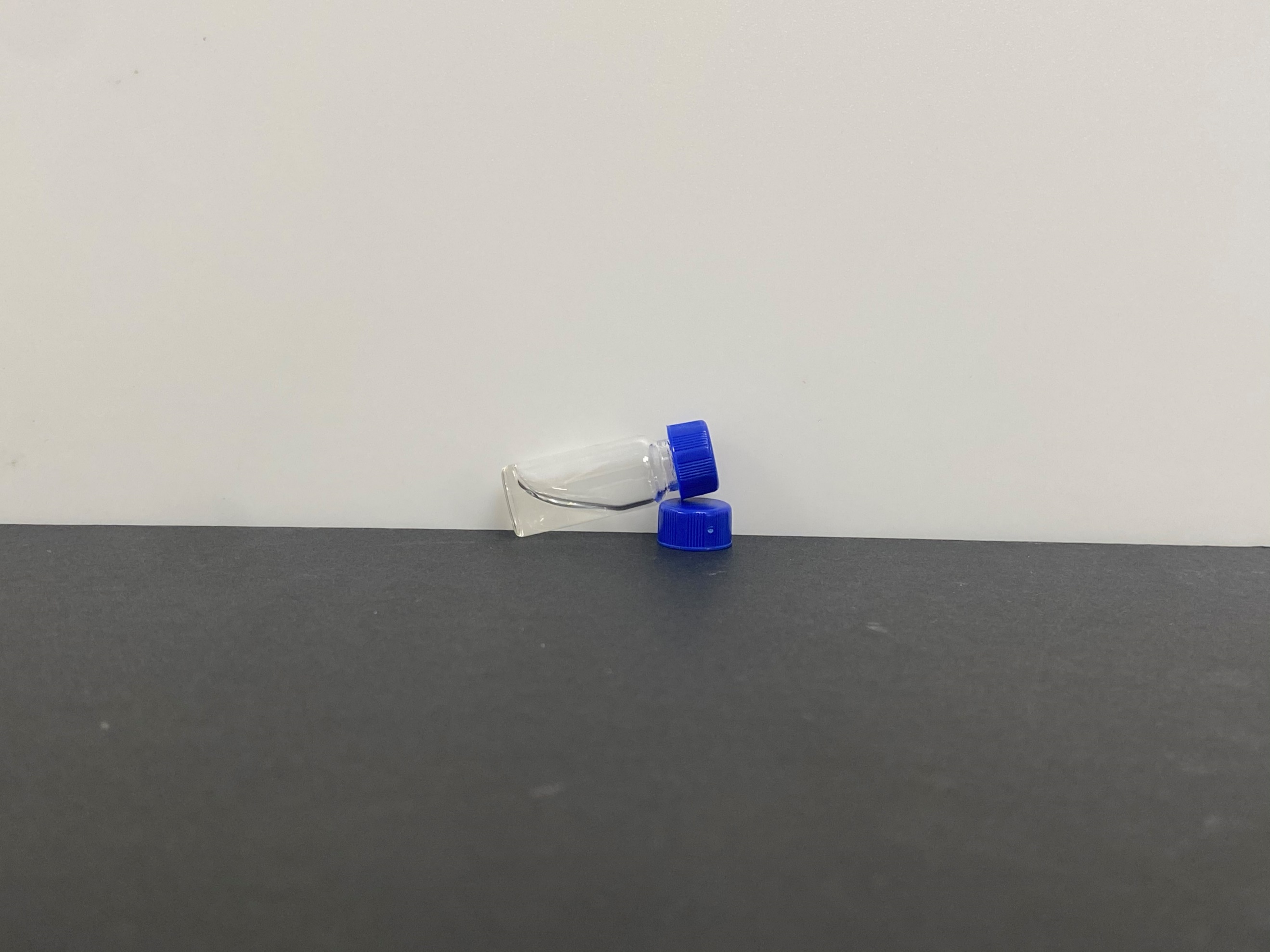

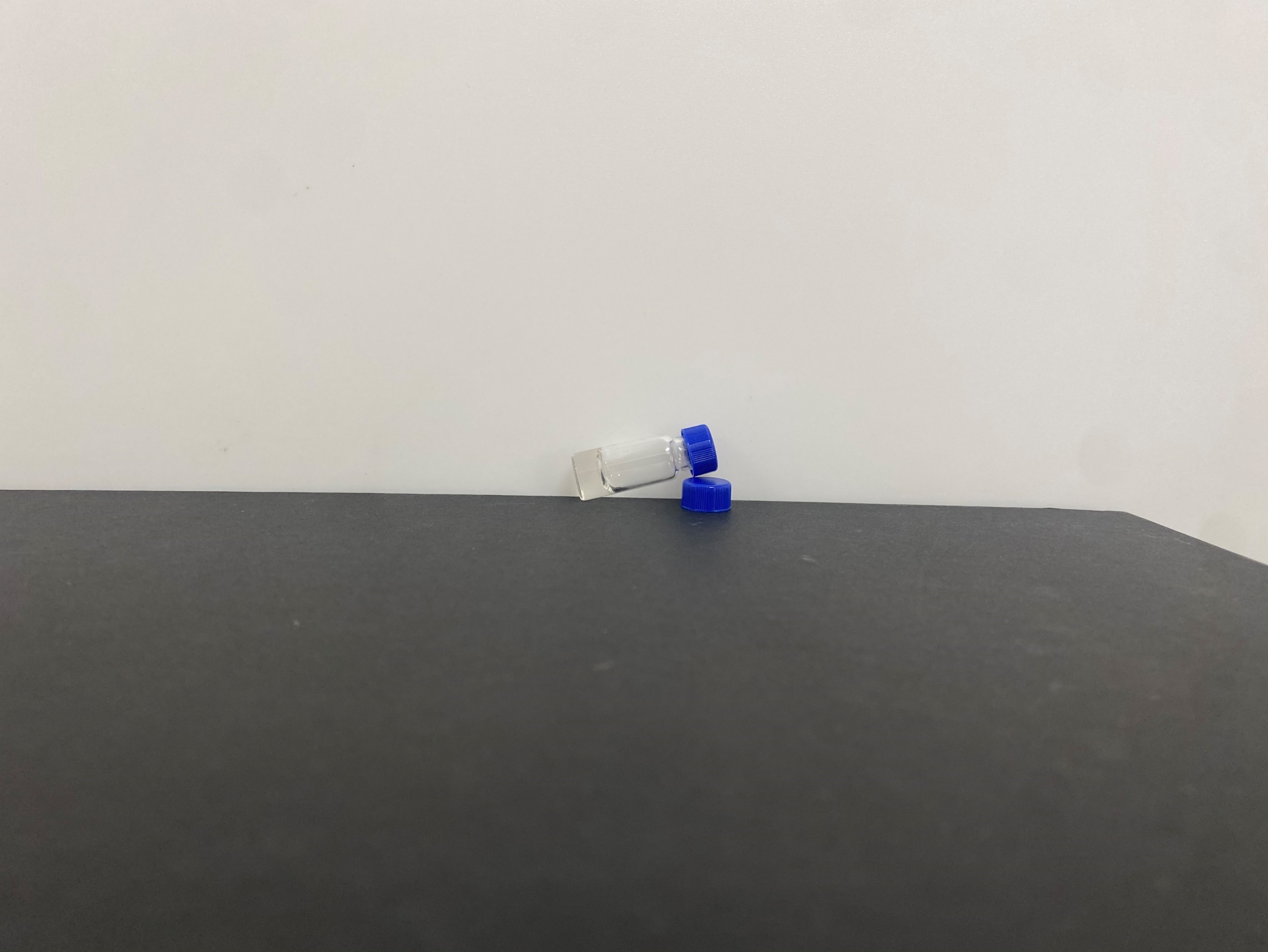

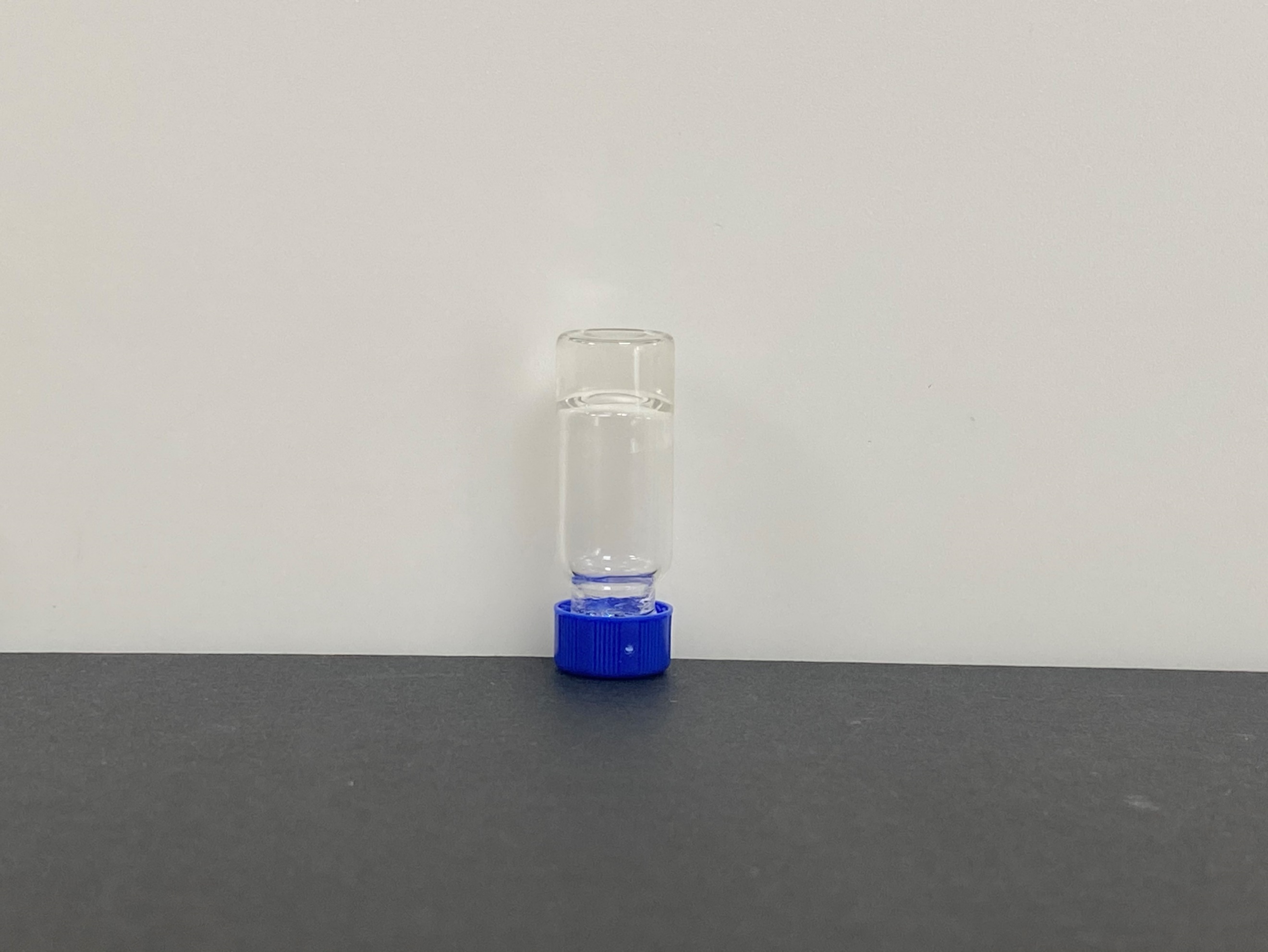

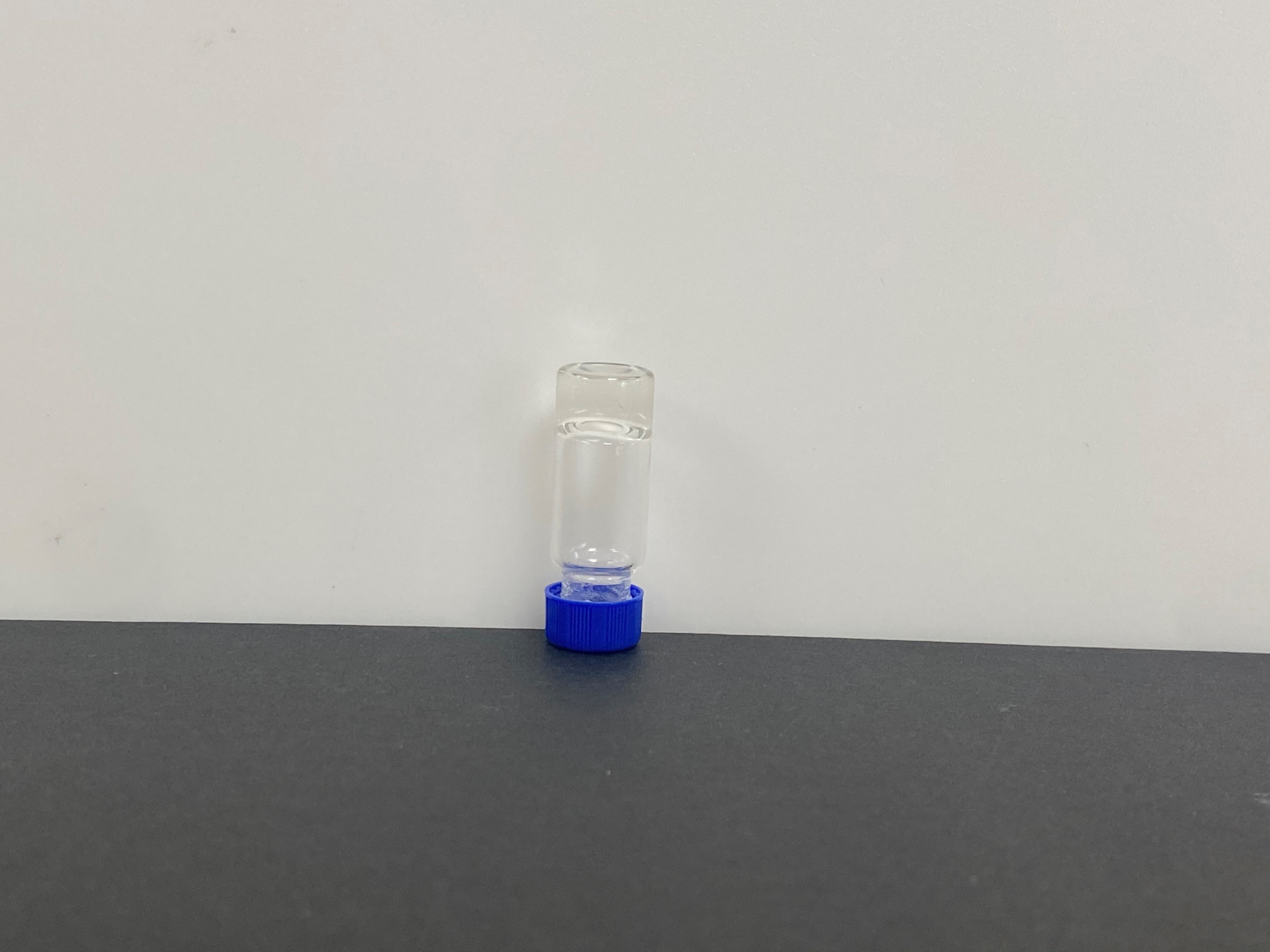

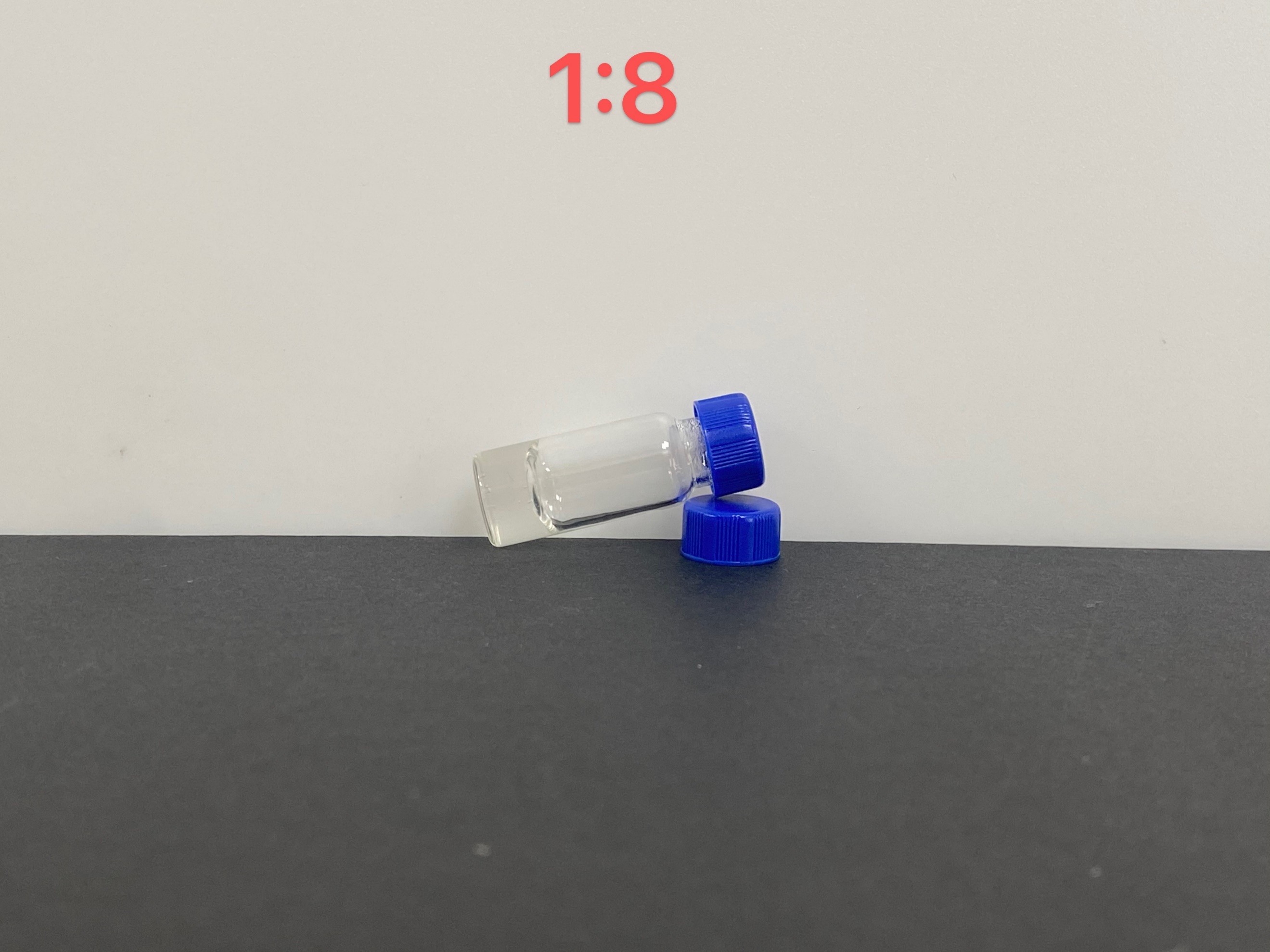


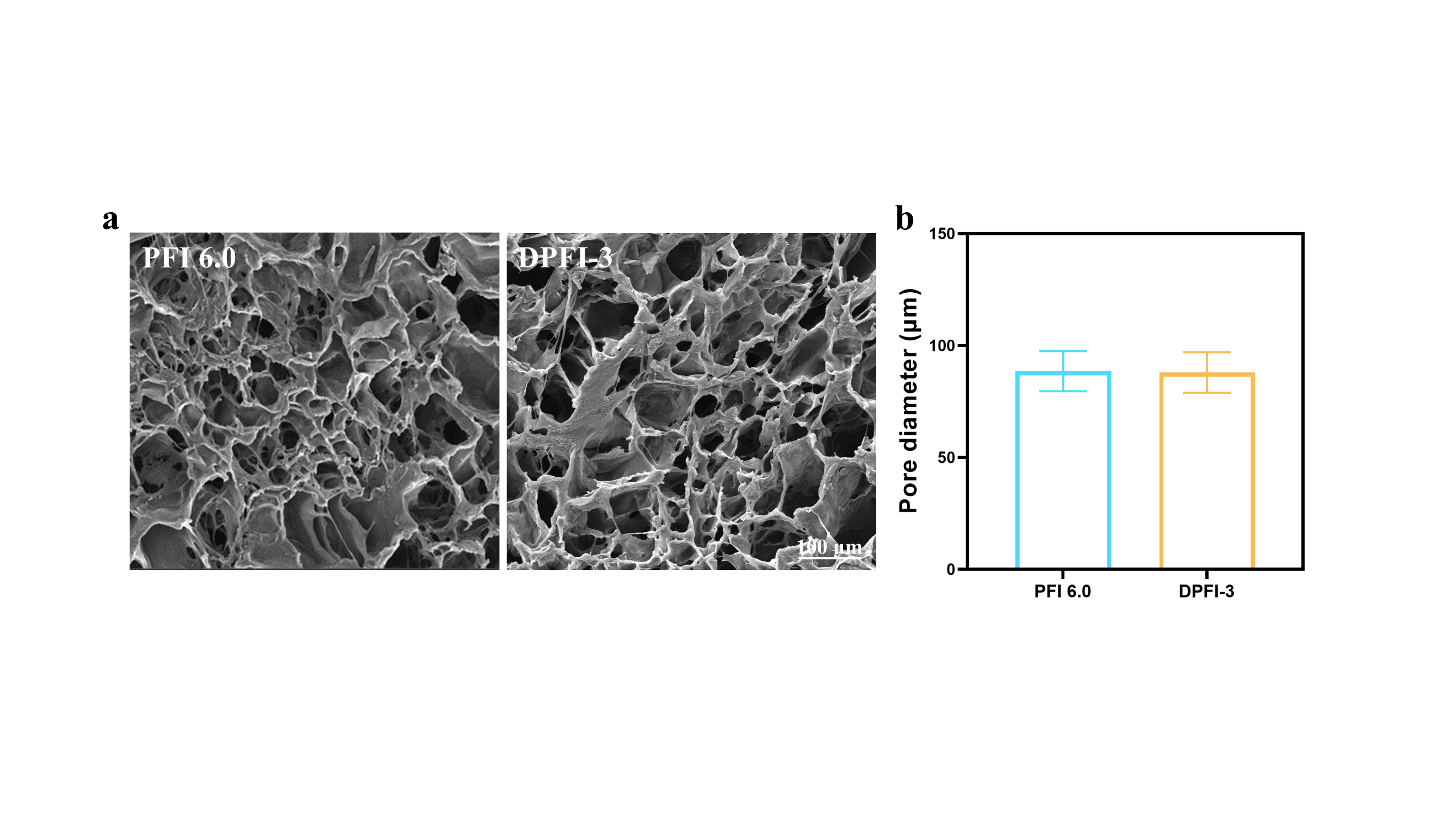


**Figure S4.** (a-b) SEM images and pore size distributions of PFI 6.0 and DPFI-3.

Supplement: Figure_S4_tkaf024 [file figure_s4_tkaf024.docx]

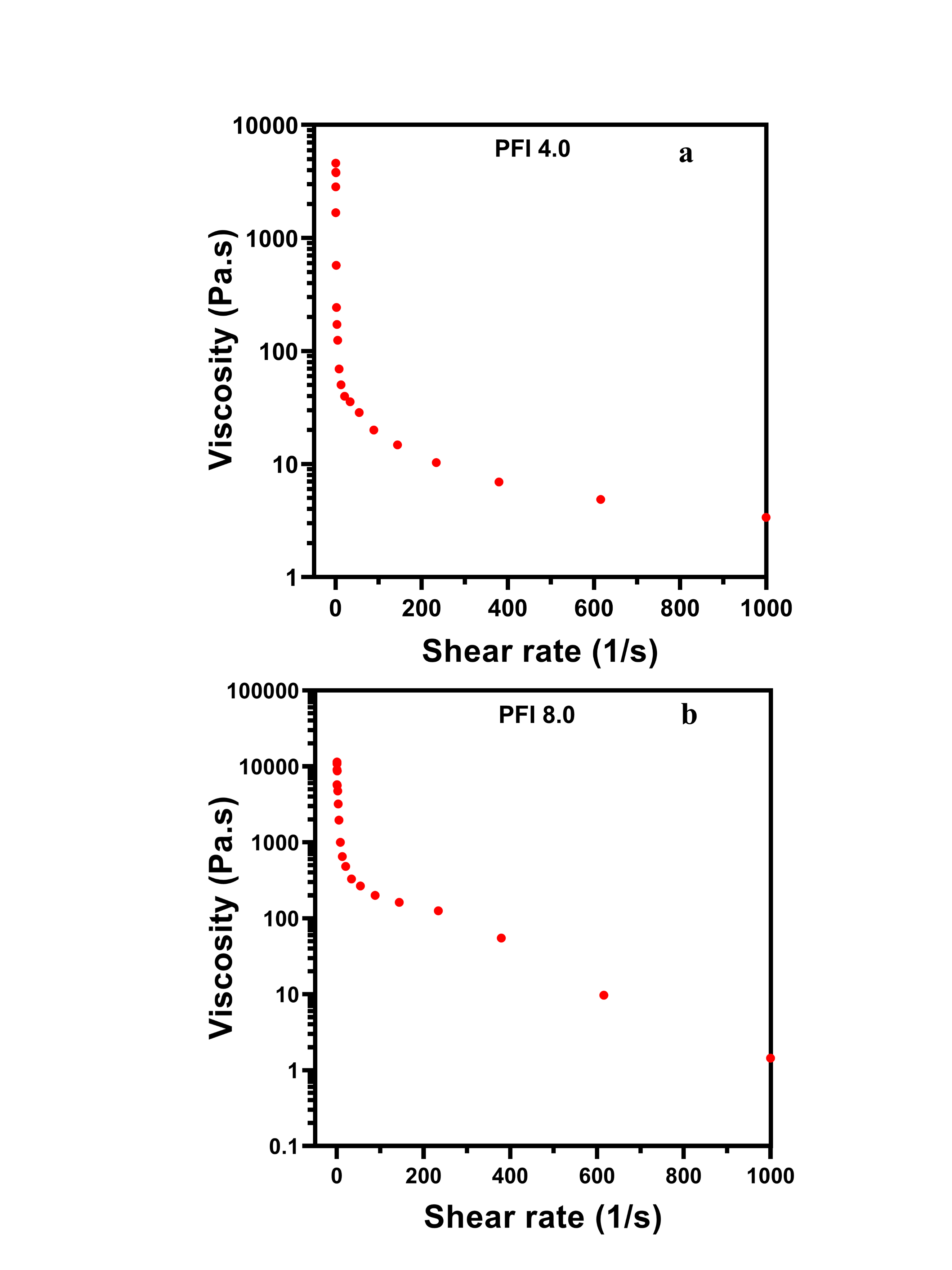


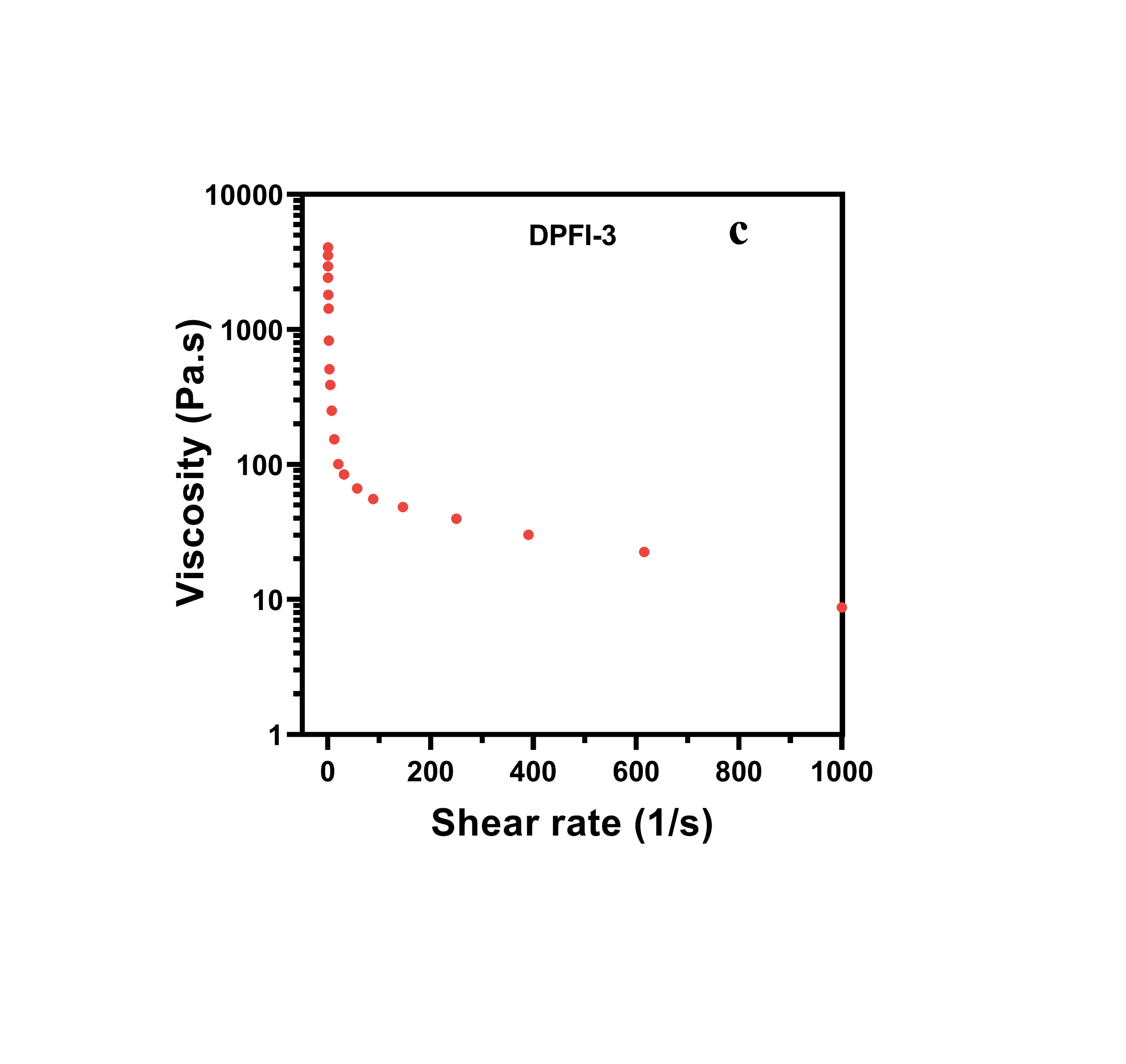


**Figure S6.** (a-c) Shear viscosity of PFI 4.0, PFI 8.0, and DPFI-3 across shear rates.

Supplement: Figure_S6_tkaf024 [file figure_s6_tkaf024.docx]

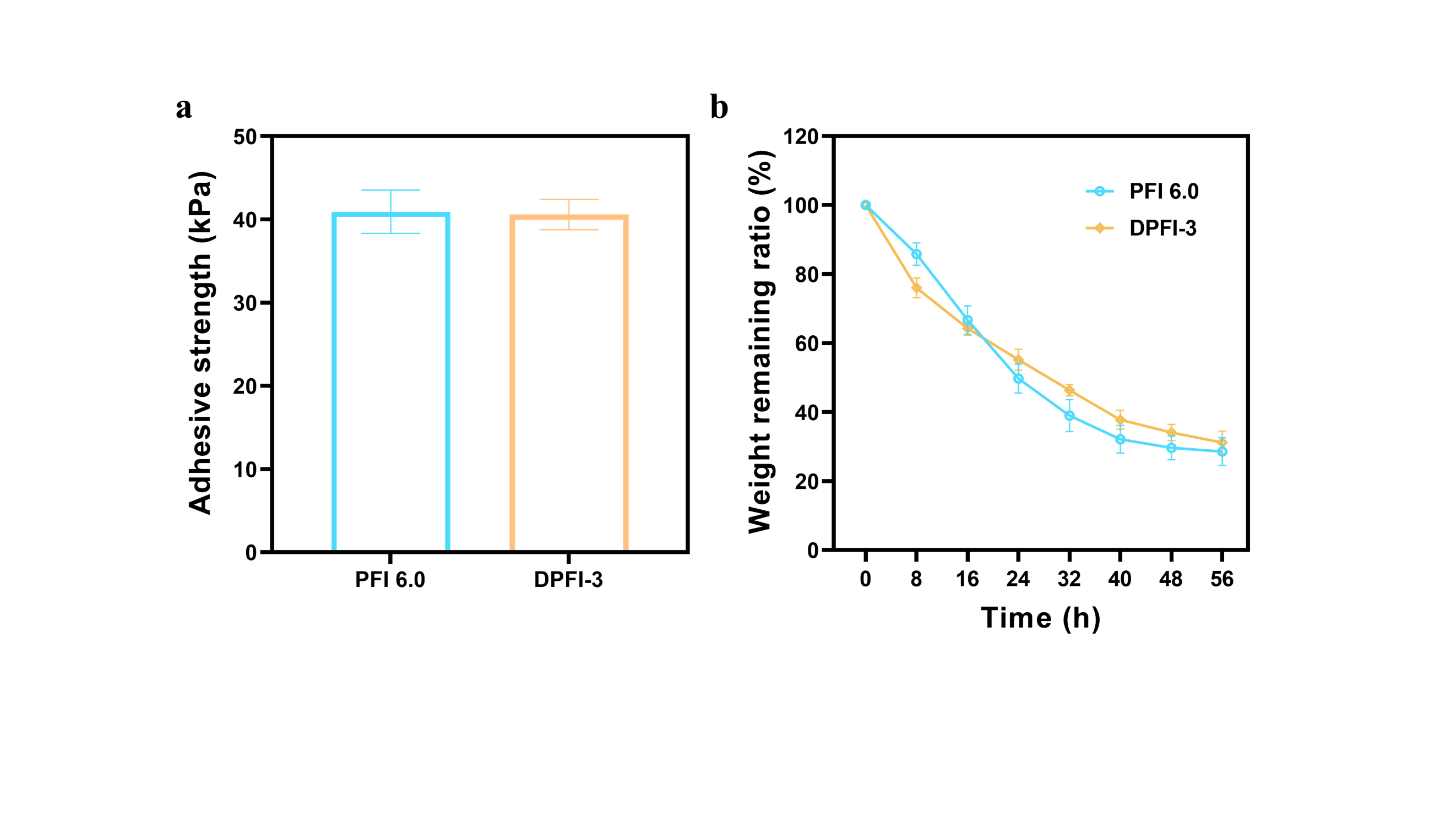


**Figure S7.** Adhesion strength of PFI 6.0 and DPFI-3 on pig skin

Supplement: Figure_S7_tkaf024 [file figure_s7_tkaf024.docx]

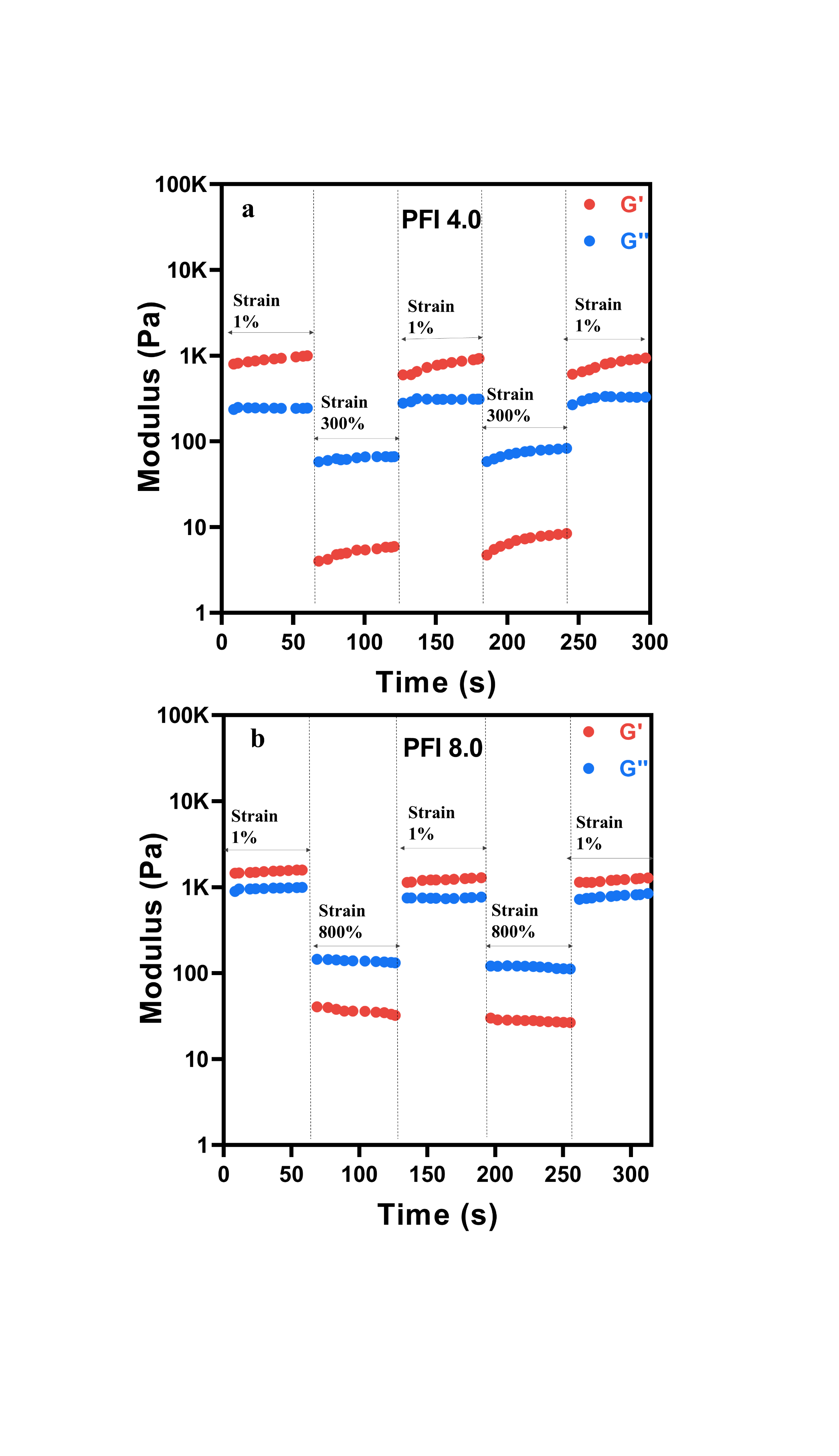


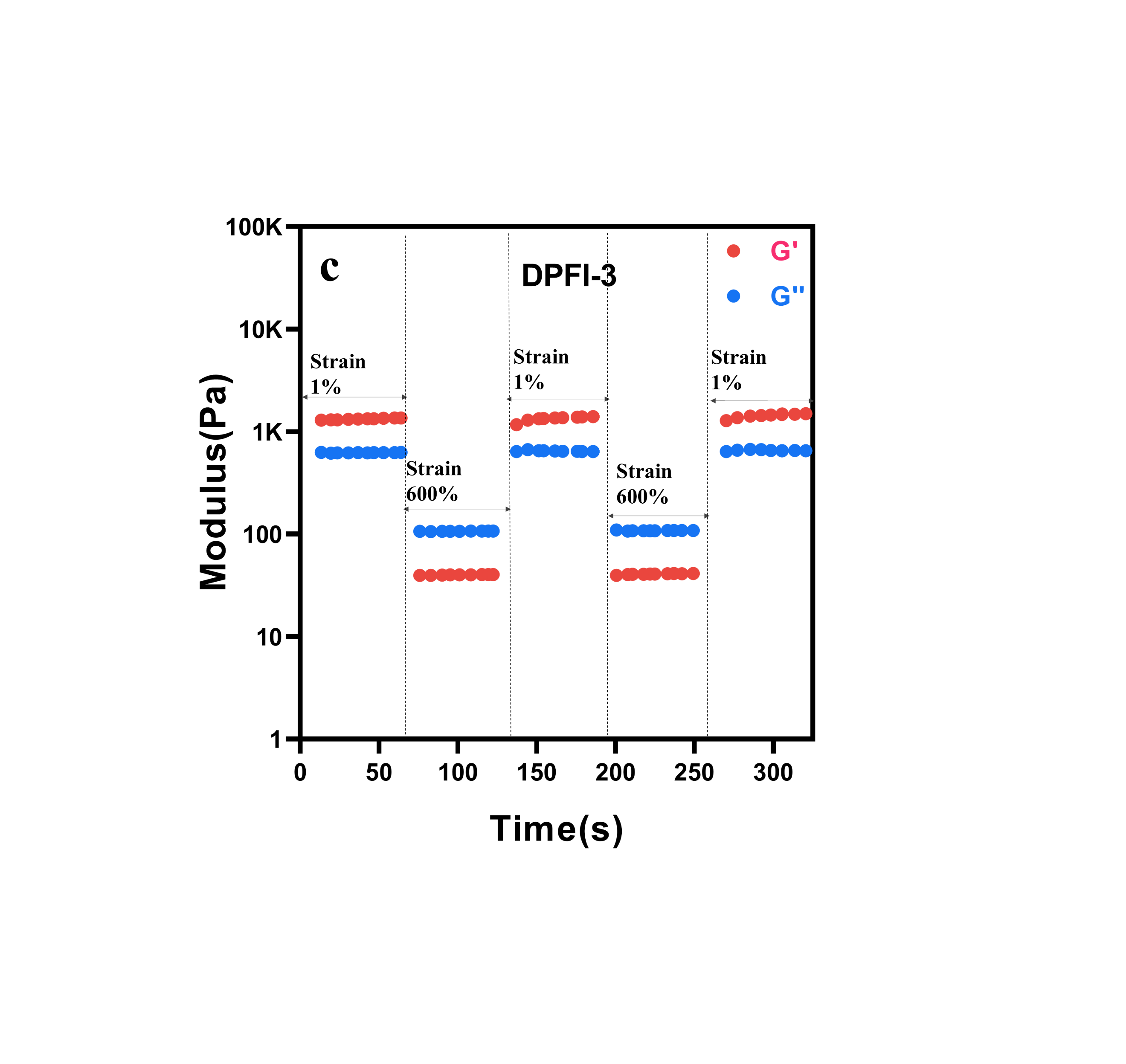


**Figure S8.** (a-c) Alternate step strain sweep tests of PFI 4.0, PFI 8.0, and DPFI-3.

Supplement: Figure_S8_tkaf024 [file figure_s8_tkaf024.docx]

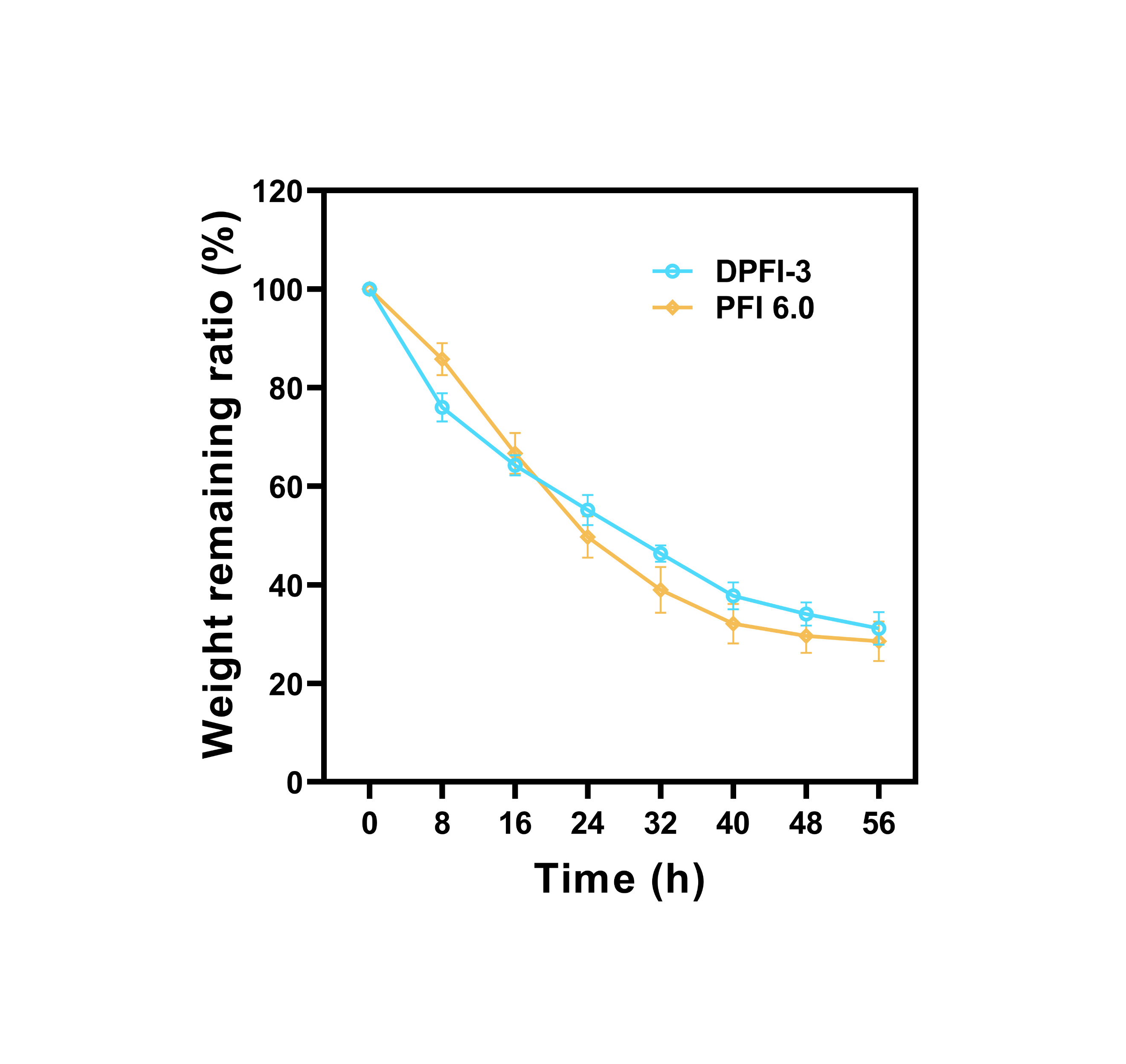


**Figure S9.** Degradation properties of PFI 6.0 and DPFI-3 in PBS at pH 7.4.

Supplement: Figure_S9_tkaf024 [file figure_s9_tkaf024.docx]

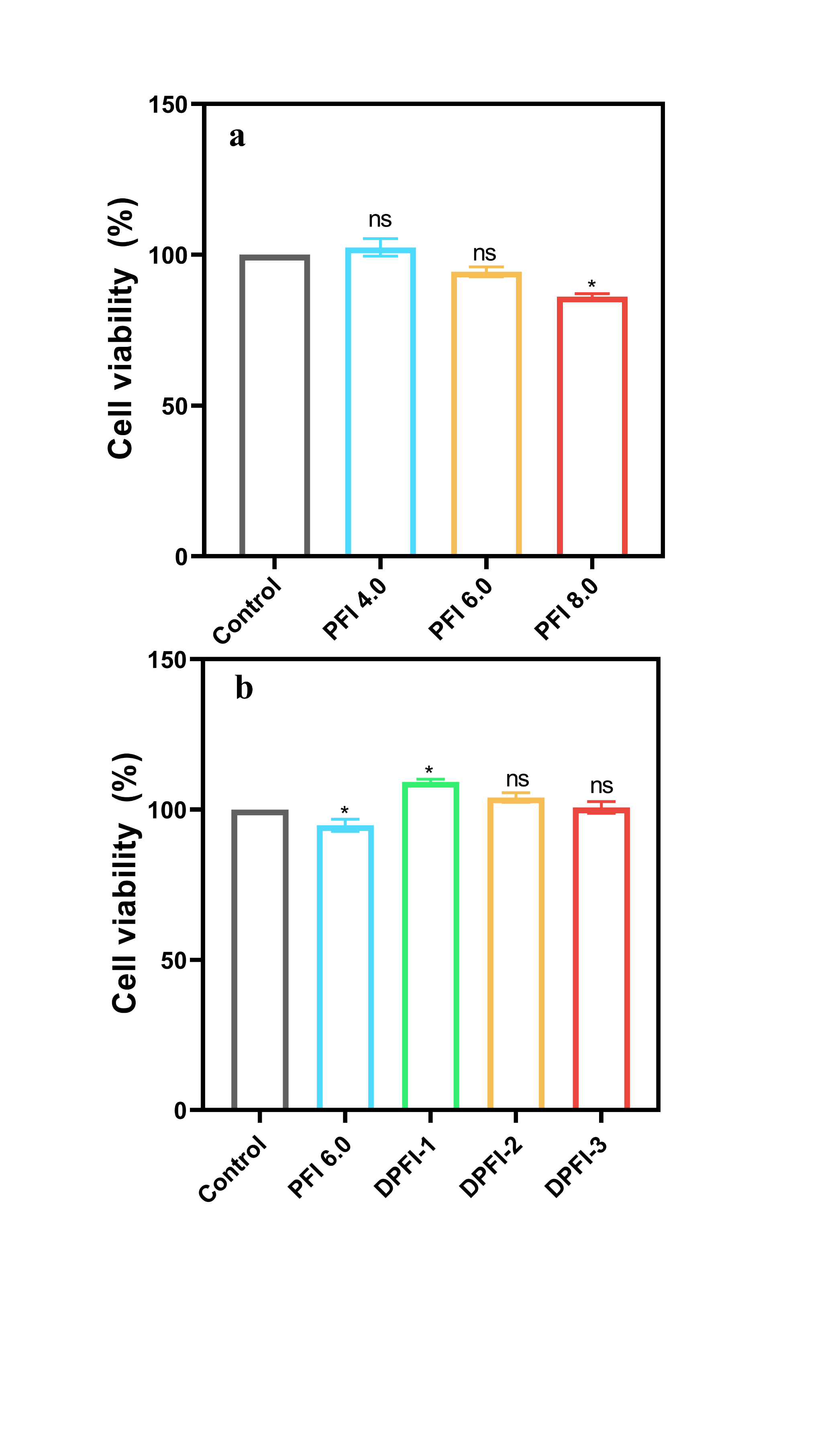


**Figure S10.** (a-b) Cytotoxicity of PFI-n and DPFI-n on L929 cells.

Supplement: Figure_S10_tkaf024 [file figure_s10_tkaf024.docx]

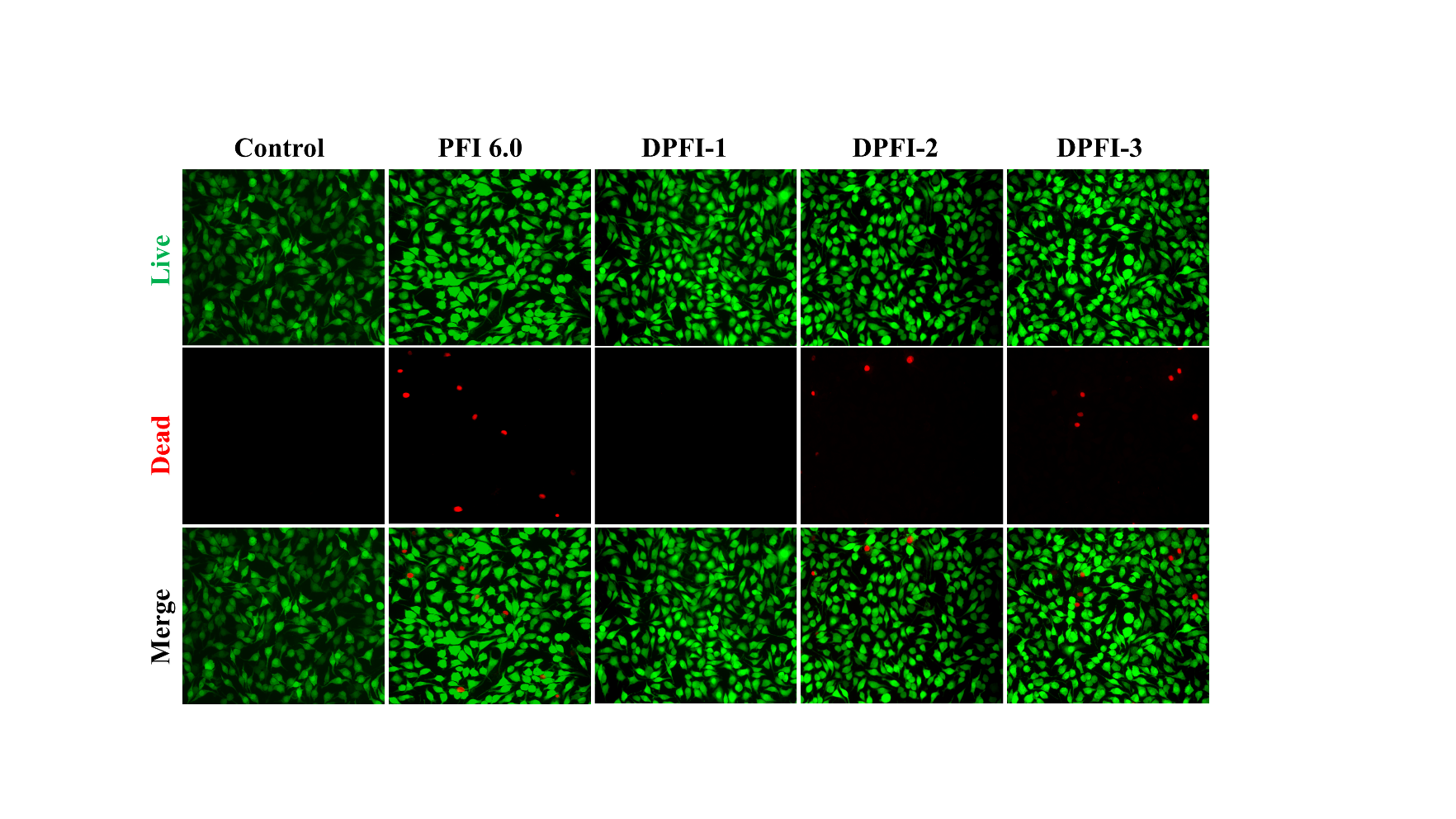


**Figure S11.** Live/dead staining of L929 cells after 24 hours of various treatments.

Supplement: Figure_S11_tkaf024 [file figure_s11_tkaf024.docx]

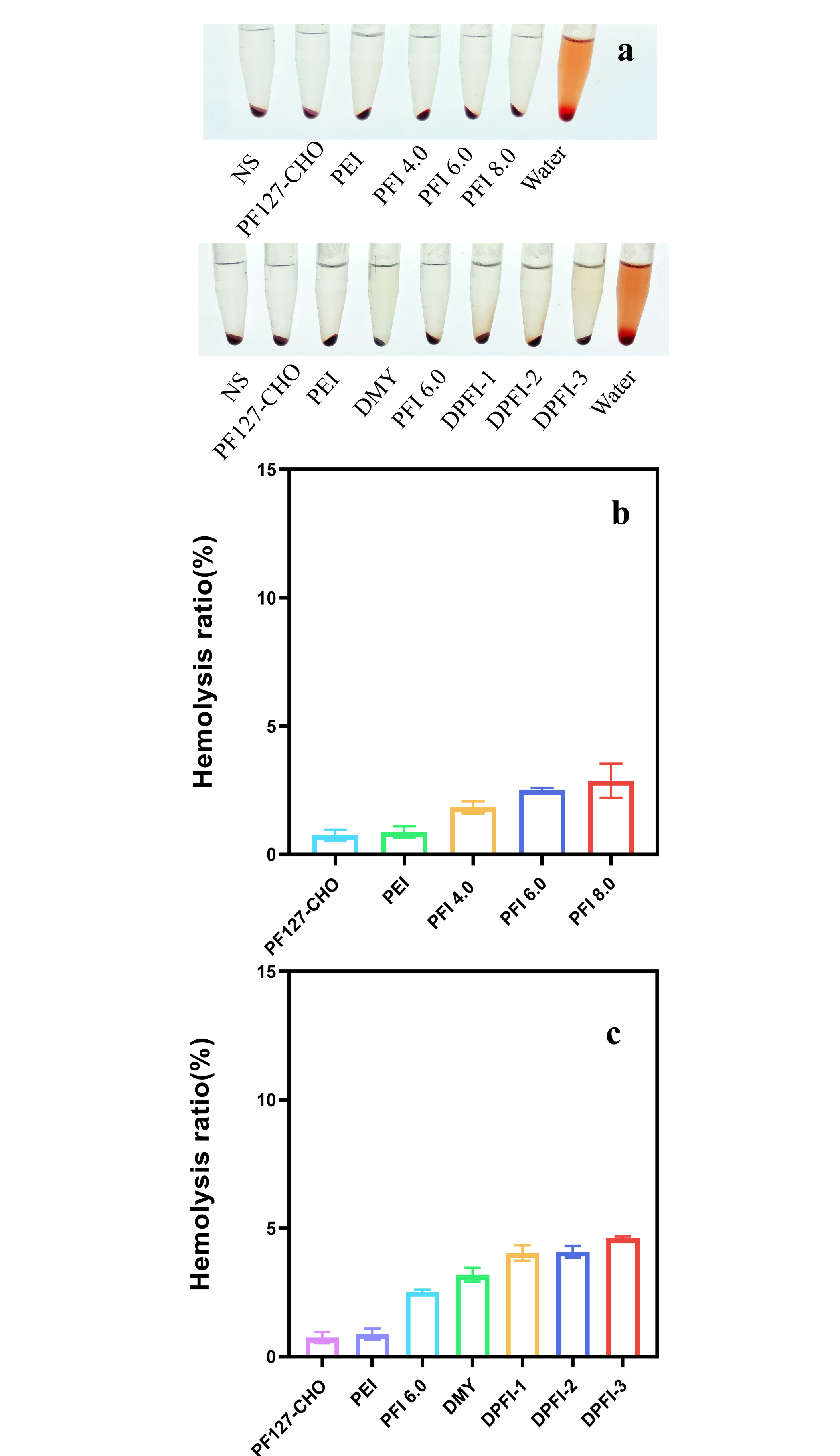


**Figure S13.** (a-c) The hemolytic ratio of PFI-n and DPFI-n.

Supplement: Figure_S13_tkaf024 [file figure_s13_tkaf024.docx]

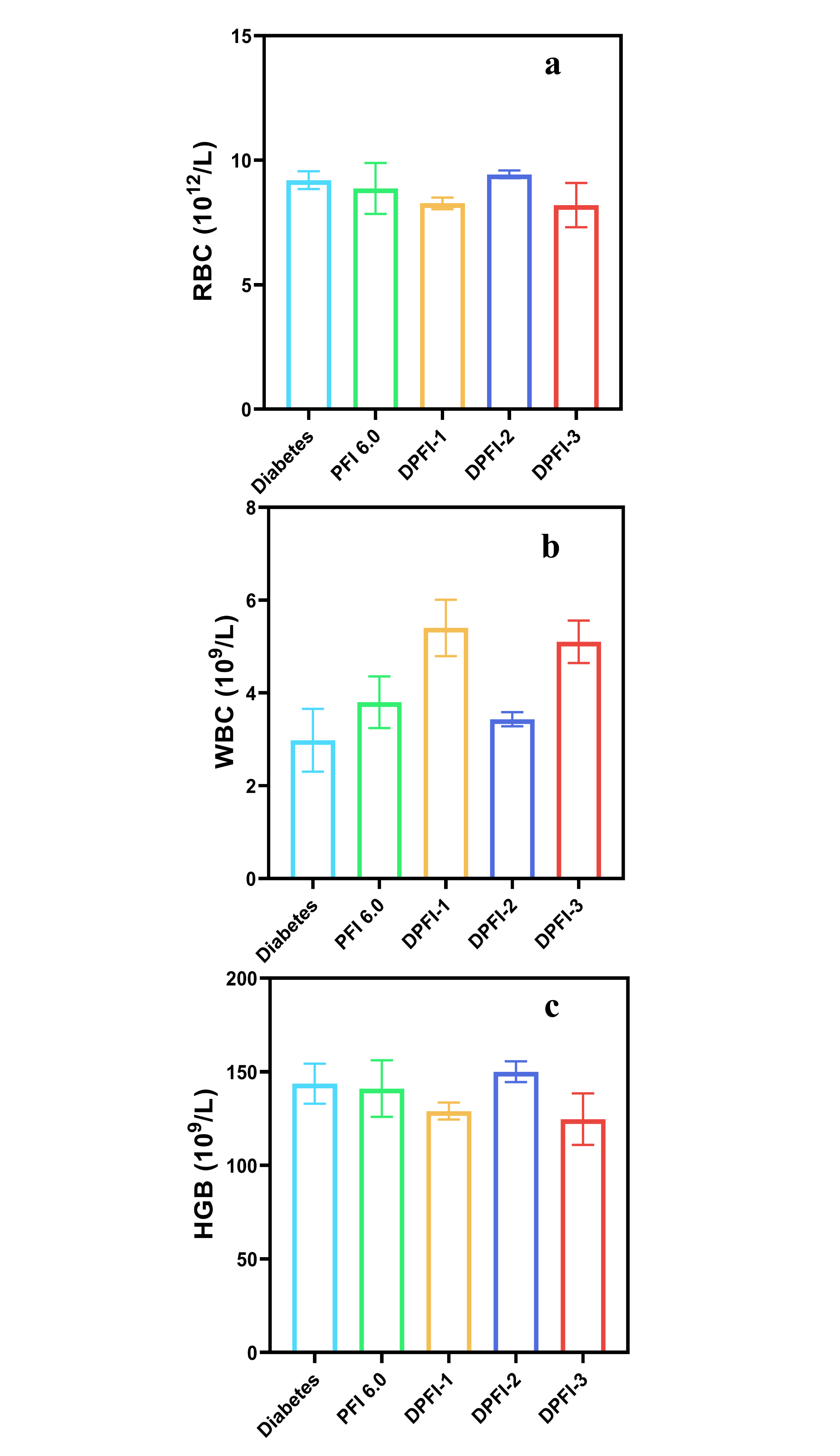


**Figure S14.** (a-c) The routine blood analysis on the 15th day.

Supplement: Figure_S14_tkaf024 [file figure_s14_tkaf024.docx]
