# Supplementary material for: A multifunctional dihydromyricetin-loaded hydrogel for the sequential modulation of diabetic wound healing and glycemic control
Source: Burns Trauma. 2025 Mar 19;13:tkaf024. doi: 10.1093/burnst/tkaf024 (PMC12315528; doi:10.1093/burnst/tkaf024)
Supplement: Figure_S5_tkaf024 [file figure_s5_tkaf024.docx]

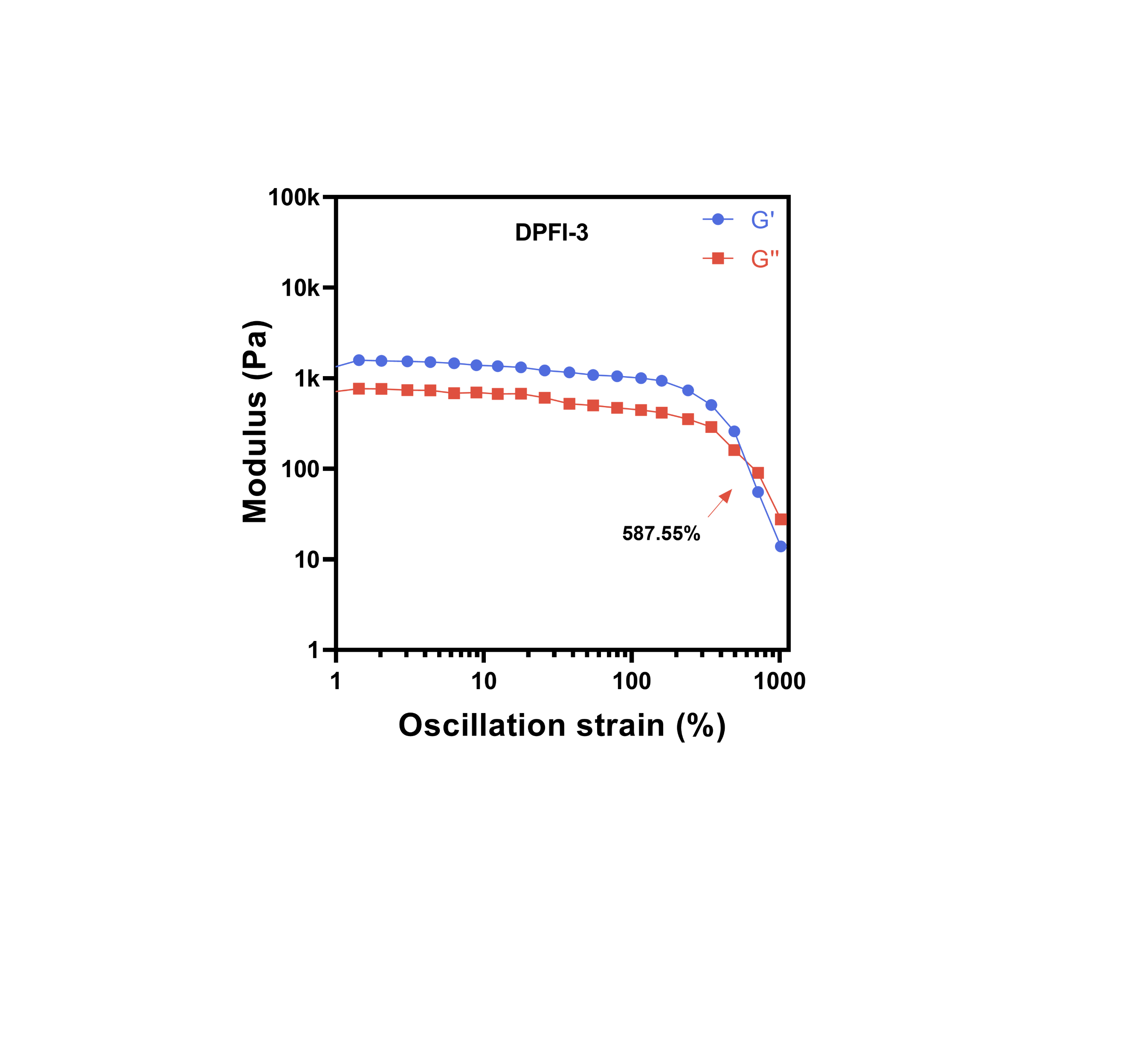


**Figure S5.** Rheological analysis depicting the storage modulus (G') and loss modulus (G'') trends of DPFI-3 as a function of strain.
