# Supplementary material for: A multifunctional dihydromyricetin-loaded hydrogel for the sequential modulation of diabetic wound healing and glycemic control
Source: Burns Trauma. 2025 Mar 19;13:tkaf024. doi: 10.1093/burnst/tkaf024 (PMC12315528; doi:10.1093/burnst/tkaf024)
Supplement: Figure_S12_tkaf024 [file figure_s12_tkaf024.docx]

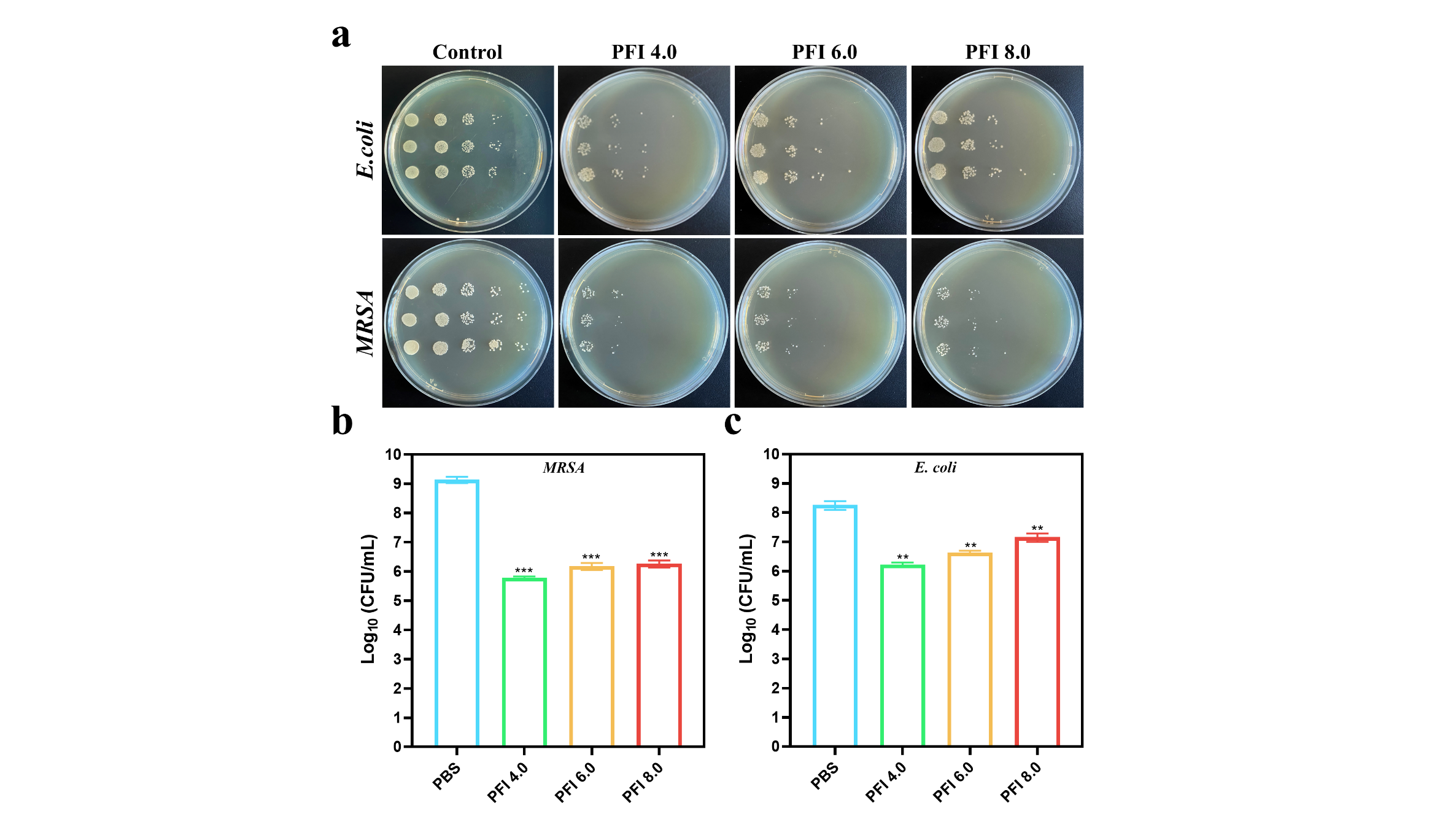


**Figure S12.** (a) Plate images and (b-c) bacterial survival numbers of *MRSA* and *E. coli* after PFI-n treatment with different ratios.
