# Supplementary material for: A multifunctional dihydromyricetin-loaded hydrogel for the sequential modulation of diabetic wound healing and glycemic control
Source: Burns Trauma. 2025 Mar 19;13:tkaf024. doi: 10.1093/burnst/tkaf024 (PMC12315528; doi:10.1093/burnst/tkaf024)
Supplement: Figure_S15_tkaf024 [file figure_s15_tkaf024.docx]

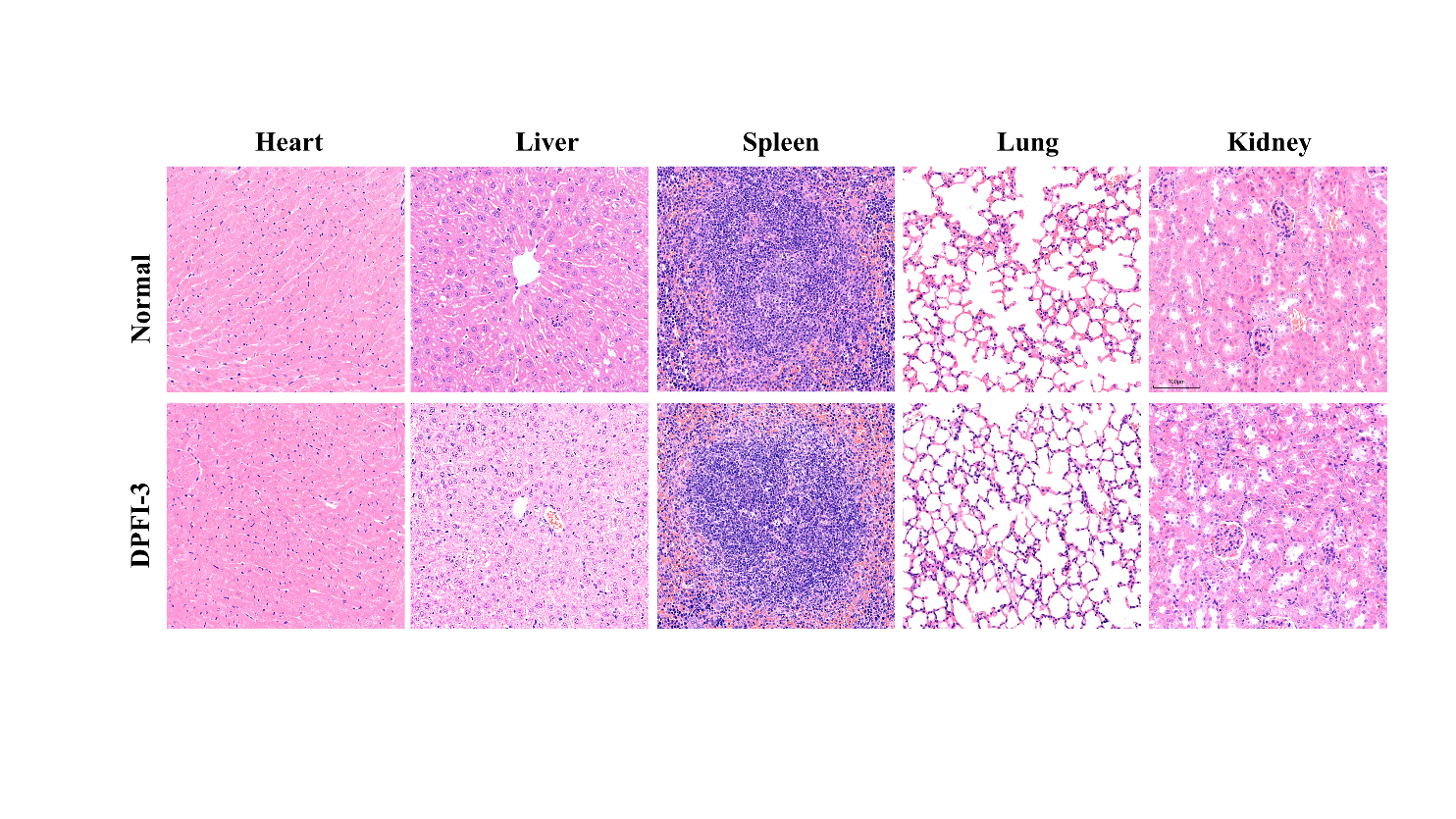


**Figure S15.** Presents a comparison between the control group and the DPFI-3 group, showcasing H&E staining of the heart, liver, spleen, lung, and kidney.
