# Supplementary material for: A multifunctional dihydromyricetin-loaded hydrogel for the sequential modulation of diabetic wound healing and glycemic control
Source: Burns Trauma. 2025 Mar 19;13:tkaf024. doi: 10.1093/burnst/tkaf024 (PMC12315528; doi:10.1093/burnst/tkaf024)
Supplement: Table_S1_tkaf024 [file table_s1_tkaf024.docx]

**Table S1.** Loading content and efficiency of DMY within F127-CHO micelles.

| **Sample** | **Loading content (%)** | **Loading efficacy (%)** |
| --- | --- | --- |
| DMY@F127-CHO_1_ | 0.62 | 62.00 |
| DMY@F127-CHO_2_ | 1.42 | 70.80 |
| DMY@F127-CHO_3_ | 2.41 | 80.40 |
