# Supplementary material for: A multifunctional dihydromyricetin-loaded hydrogel for the sequential modulation of diabetic wound healing and glycemic control
Source: Burns Trauma. 2025 Mar 19;13:tkaf024. doi: 10.1093/burnst/tkaf024 (PMC12315528; doi:10.1093/burnst/tkaf024)

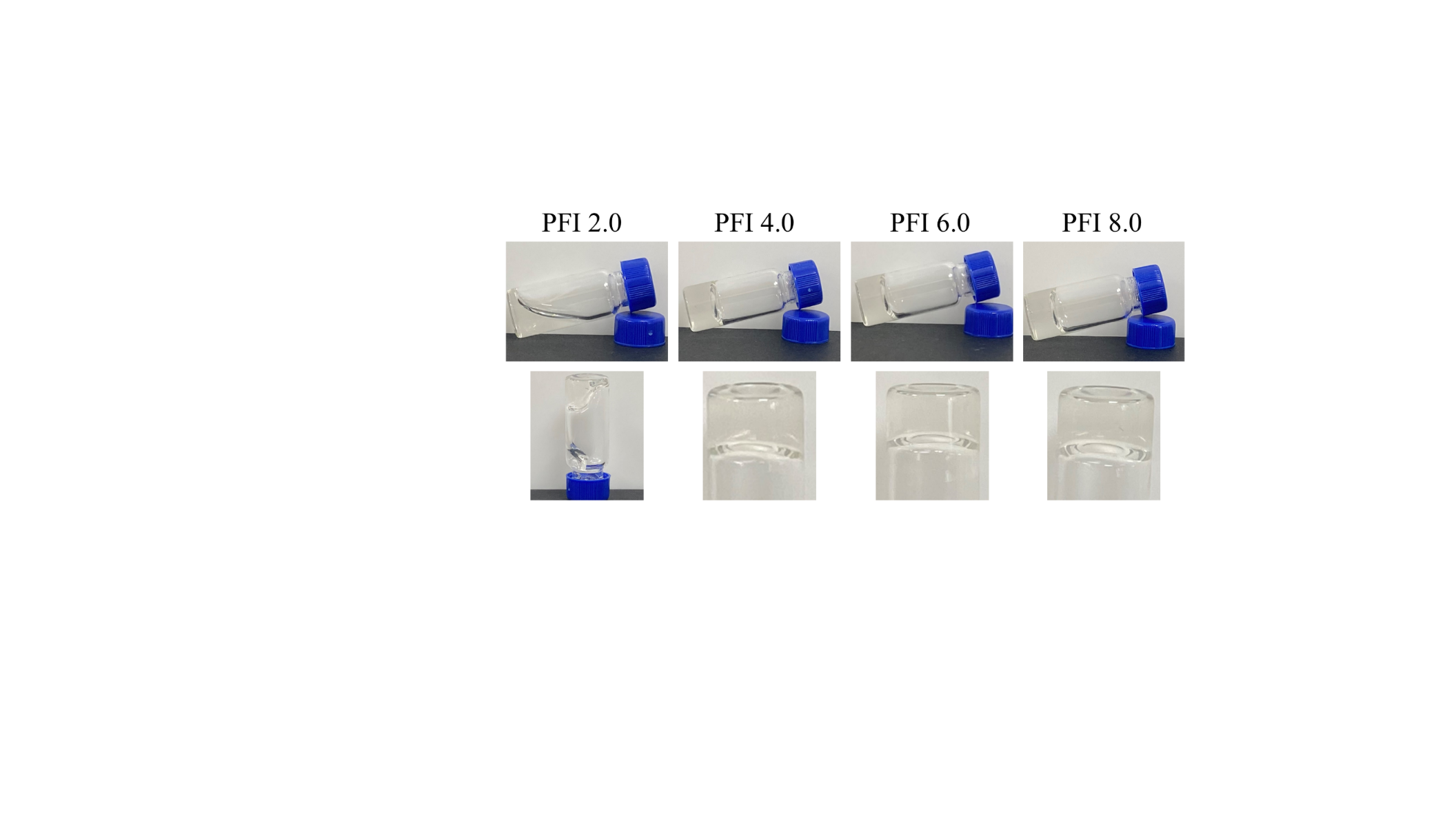


**Table S2.** Different proportion of hydrogel gel time.

| **Samples** | **PEI (wt/vol)** | **PF127-CHO**  **(wt/vol)** | **PF127-CHO/PEI** | **Gelation time (s)** |
| --- | --- | --- | --- | --- |
| PFI 2.0 | 3% | 22% | 2 | ~9 min |
| PFI 4.0 | 3% | 22% | 4 | ~60s |
| PFI 6.0 | 3% | 22% | 6 | ~35s |
| PFI 8.0 | 3% | 22% | 8 | ~22s |


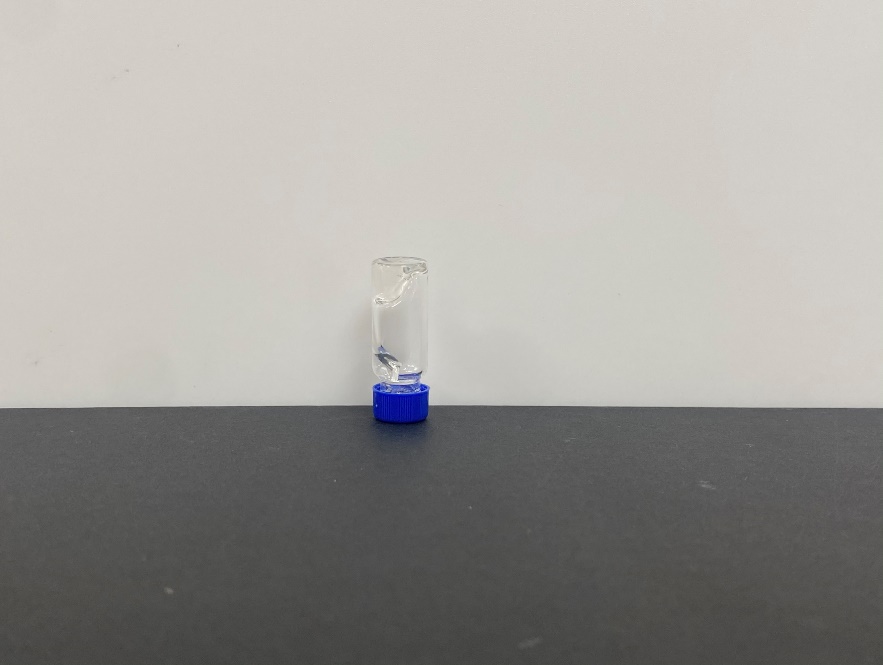

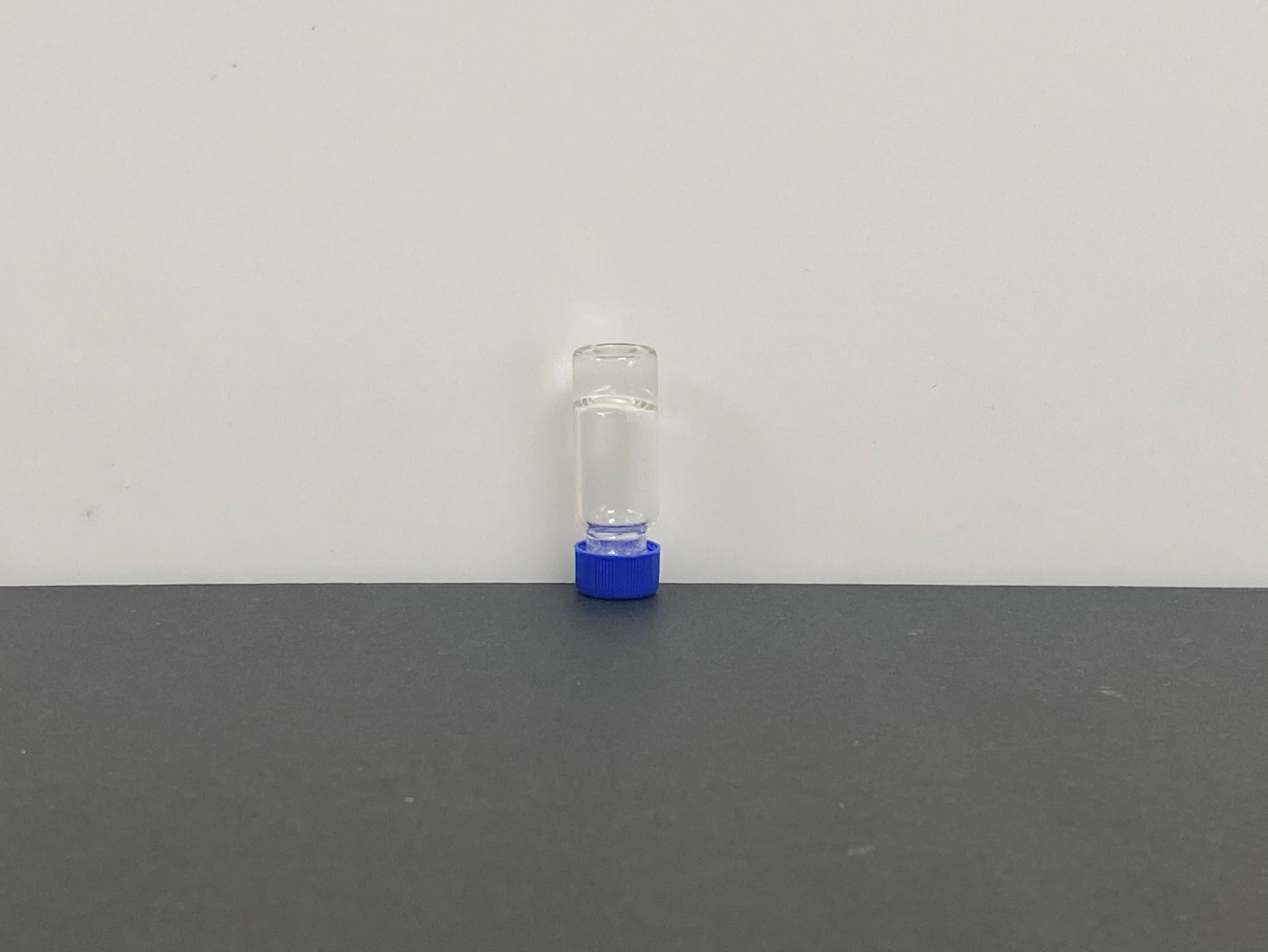

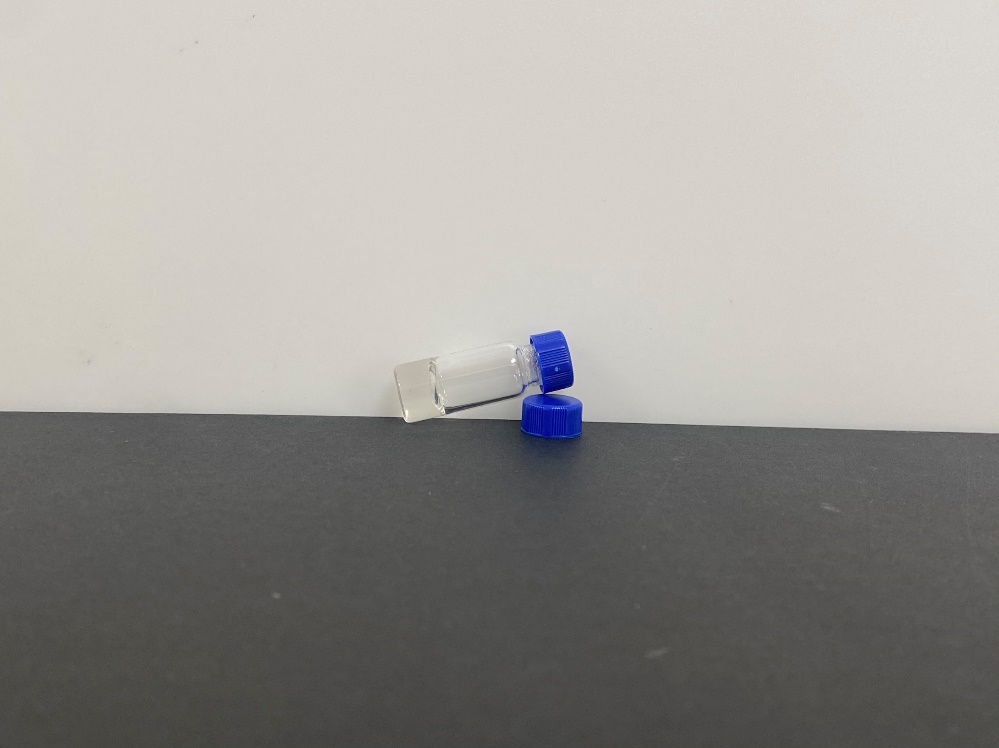

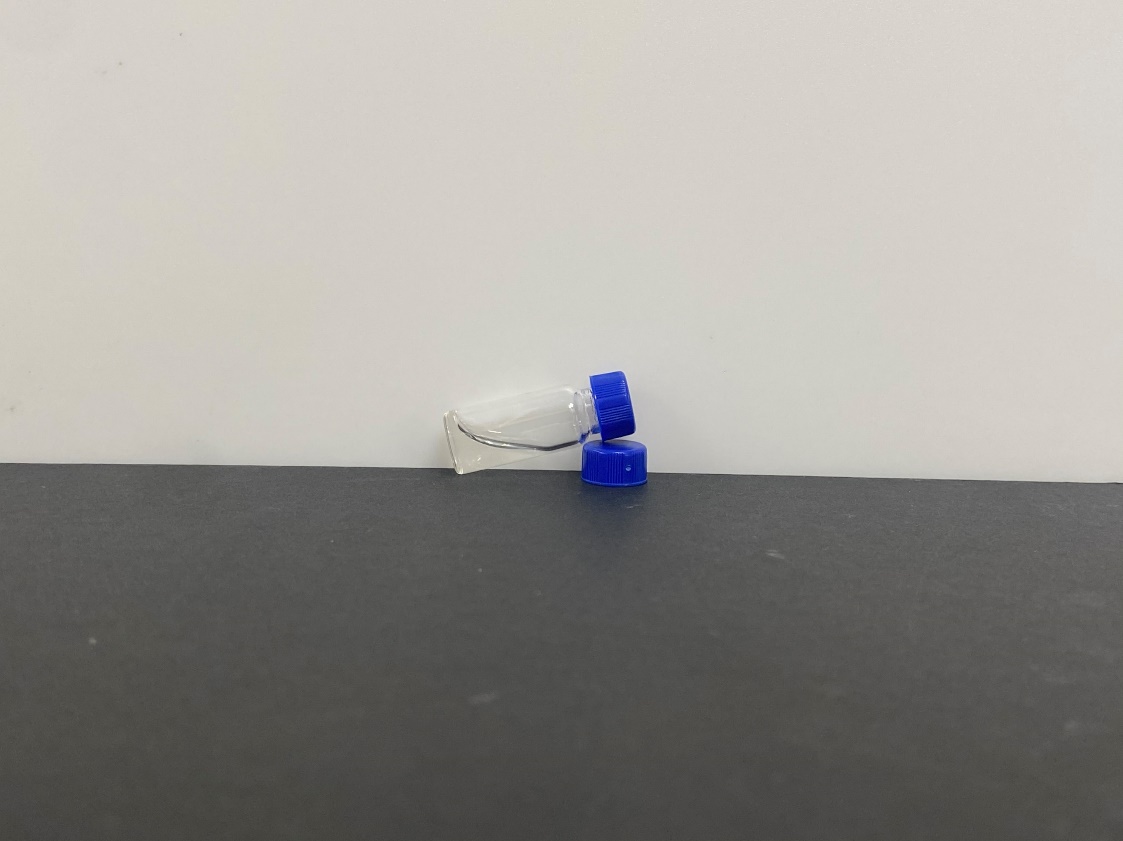

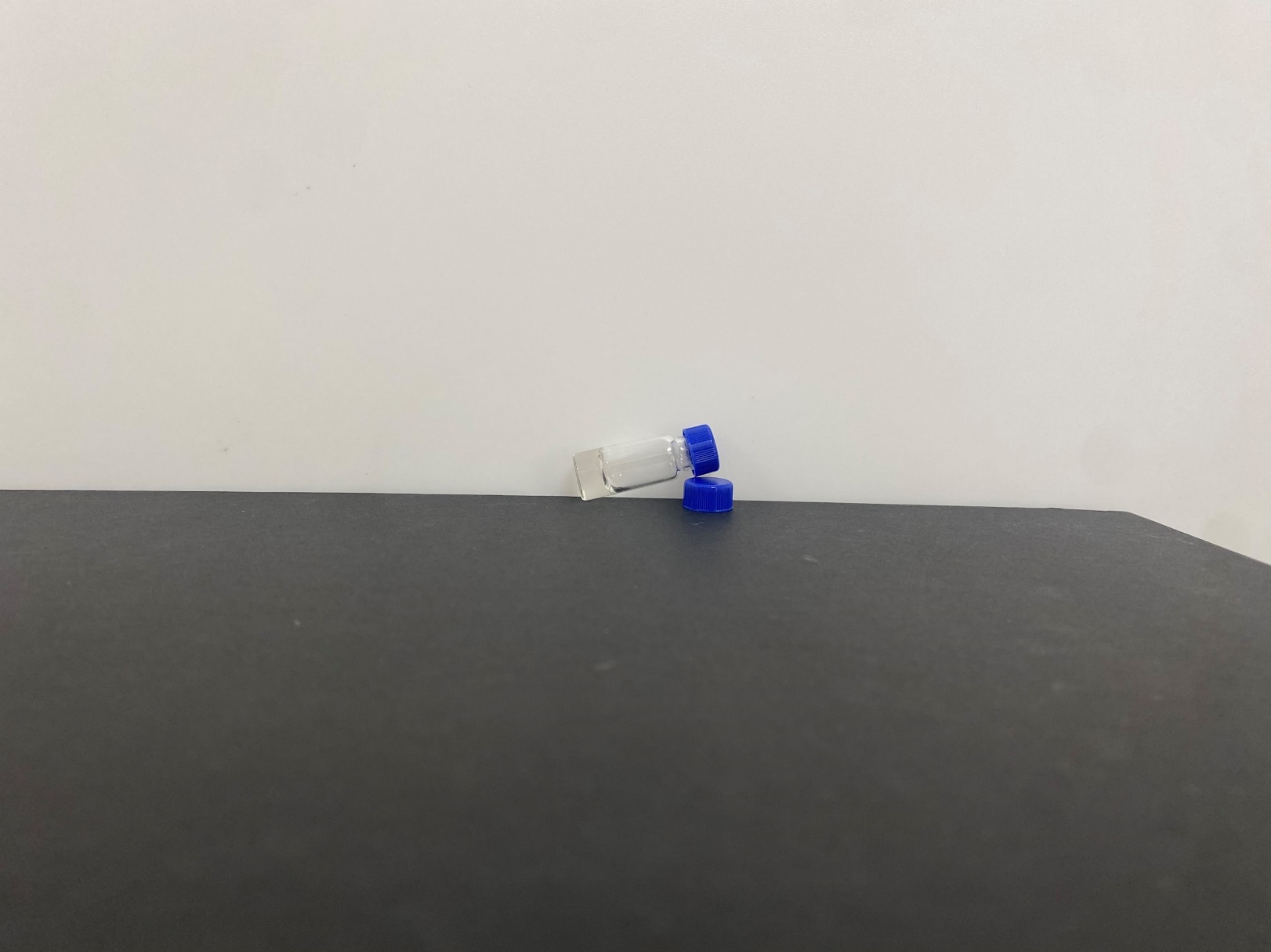

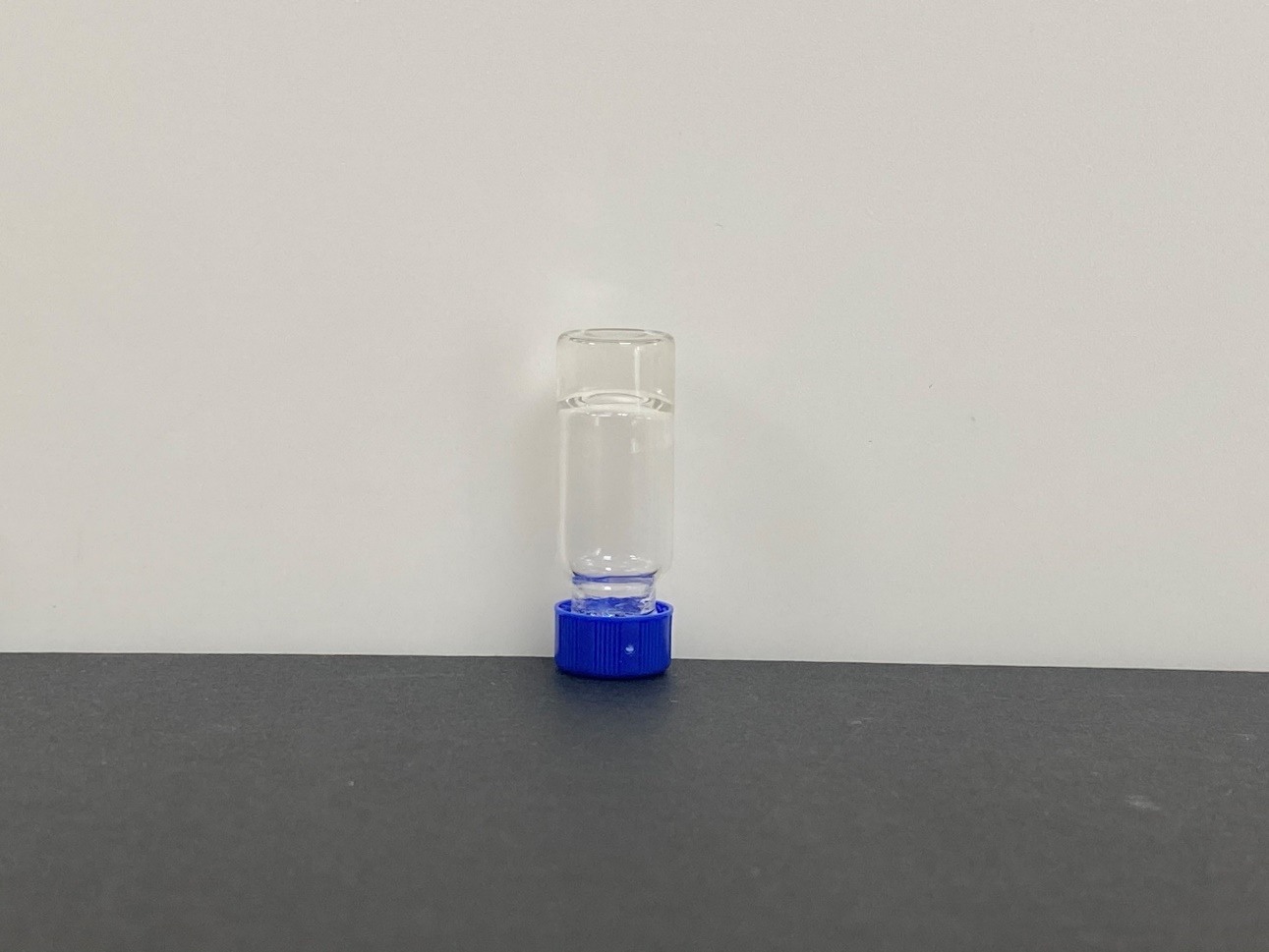

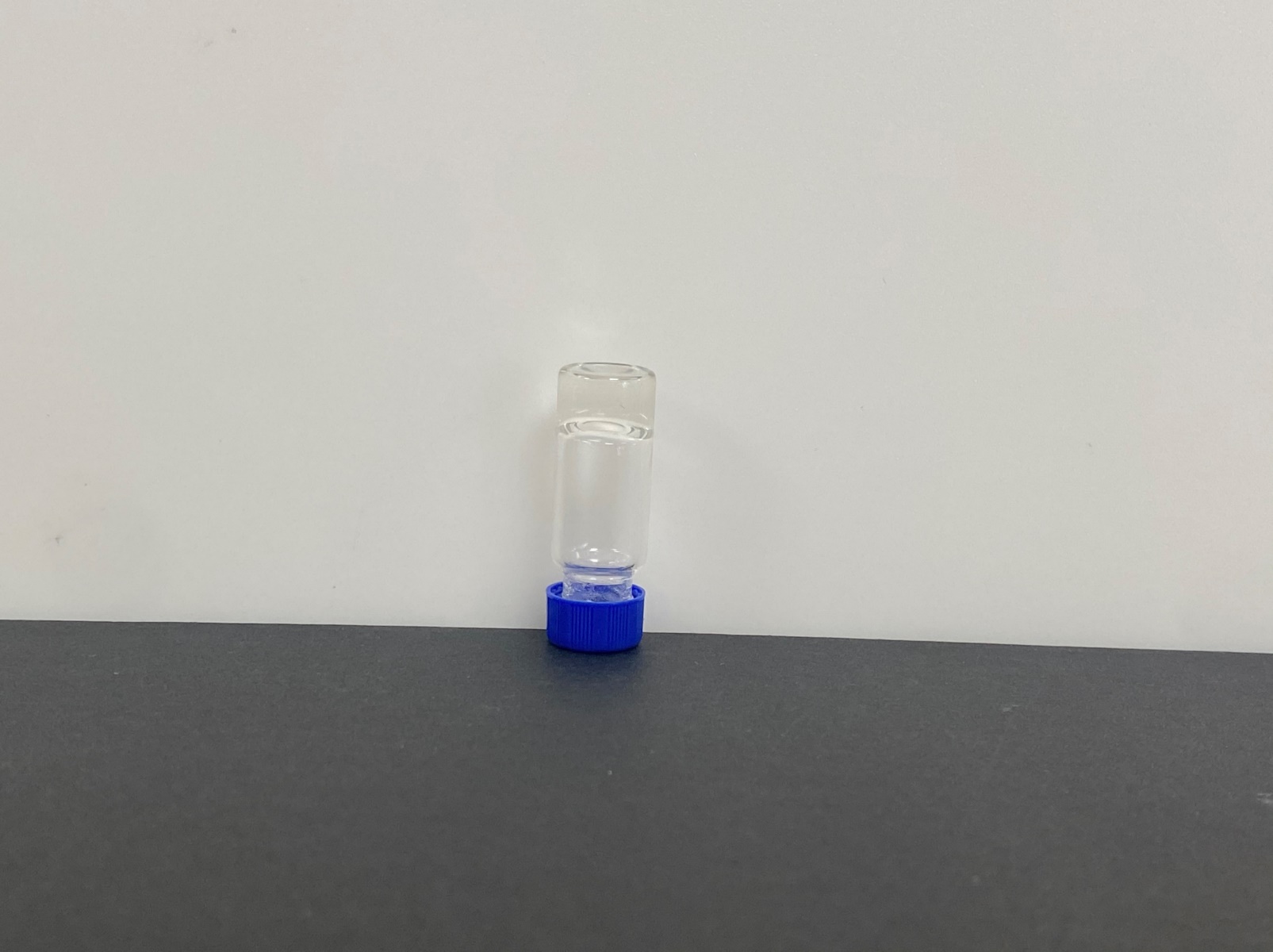

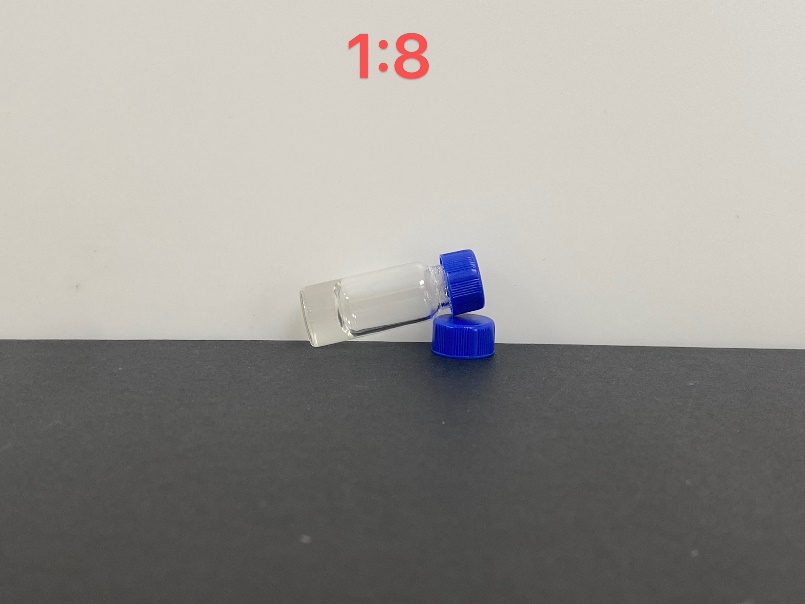

Supplement: Table_S2_tkaf024 [file table_s2_tkaf024.docx]
