# Supplementary material for: A multifunctional dihydromyricetin-loaded hydrogel for the sequential modulation of diabetic wound healing and glycemic control
Source: Burns Trauma. 2025 Mar 19;13:tkaf024. doi: 10.1093/burnst/tkaf024 (PMC12315528; doi:10.1093/burnst/tkaf024)
Supplement: Table_S3_tkaf024 [file table_s3_tkaf024.docx]

**Table S3.** Real-time polymerase chain reaction primers used in this study.


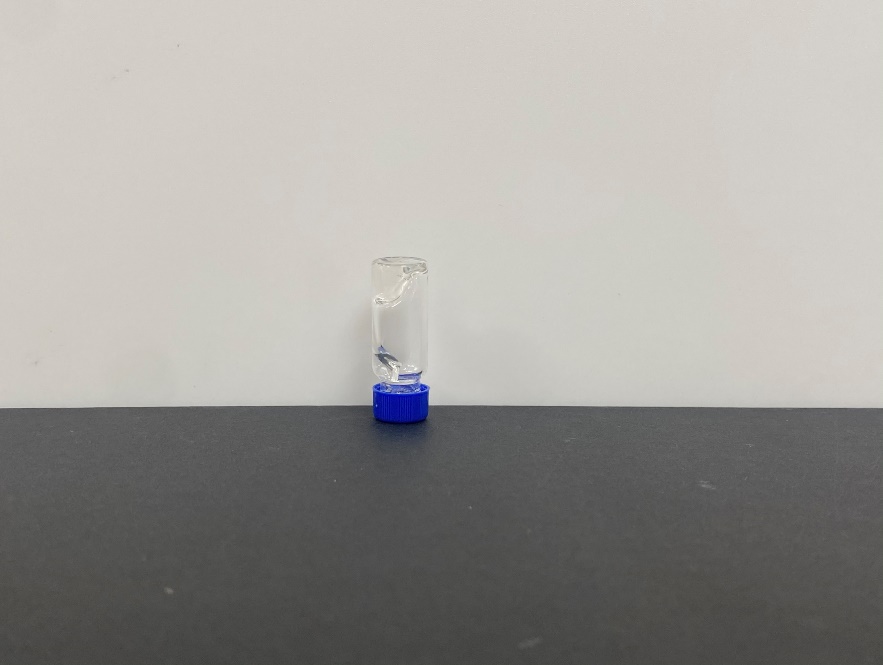

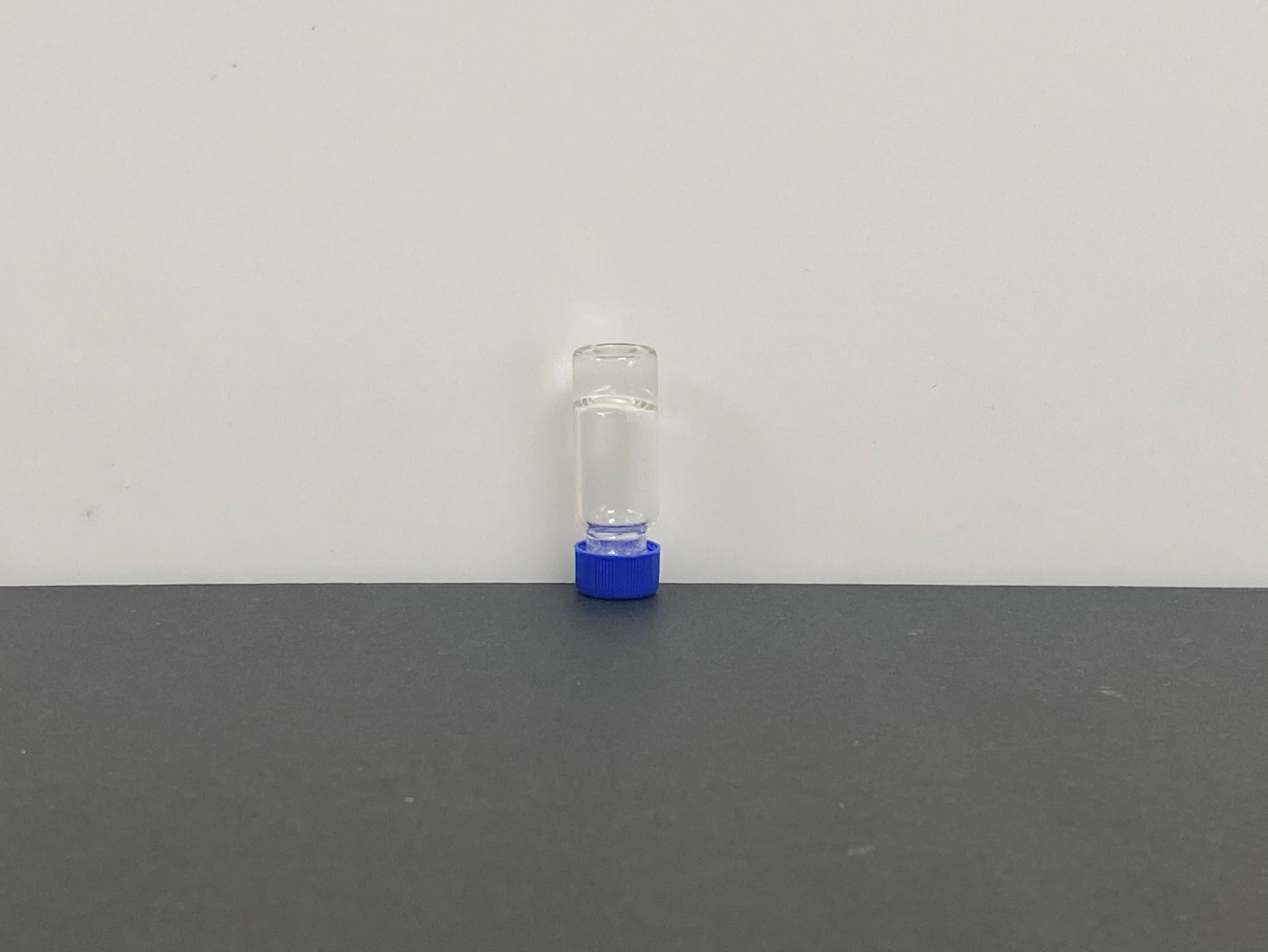

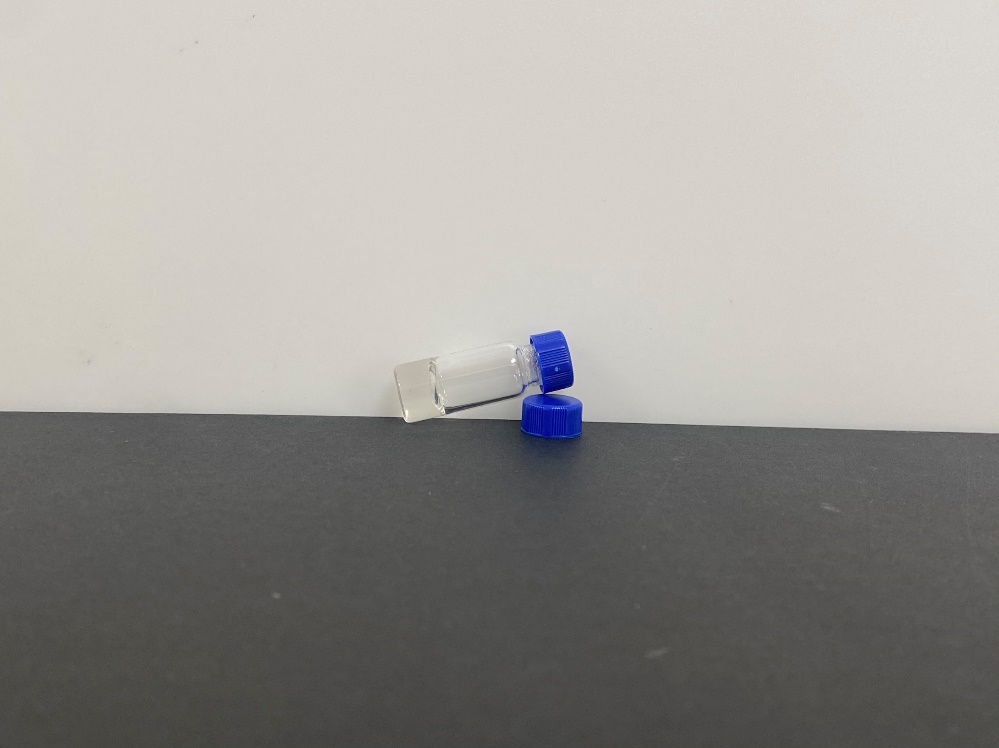

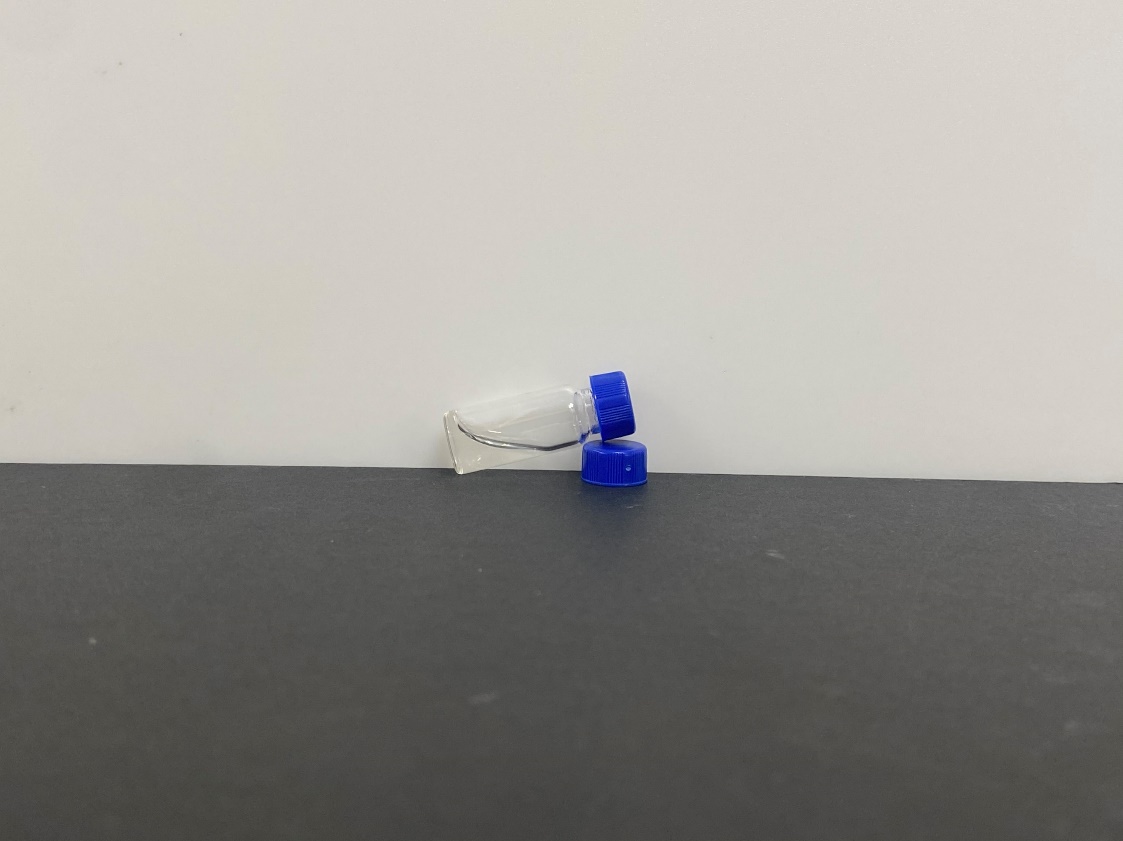

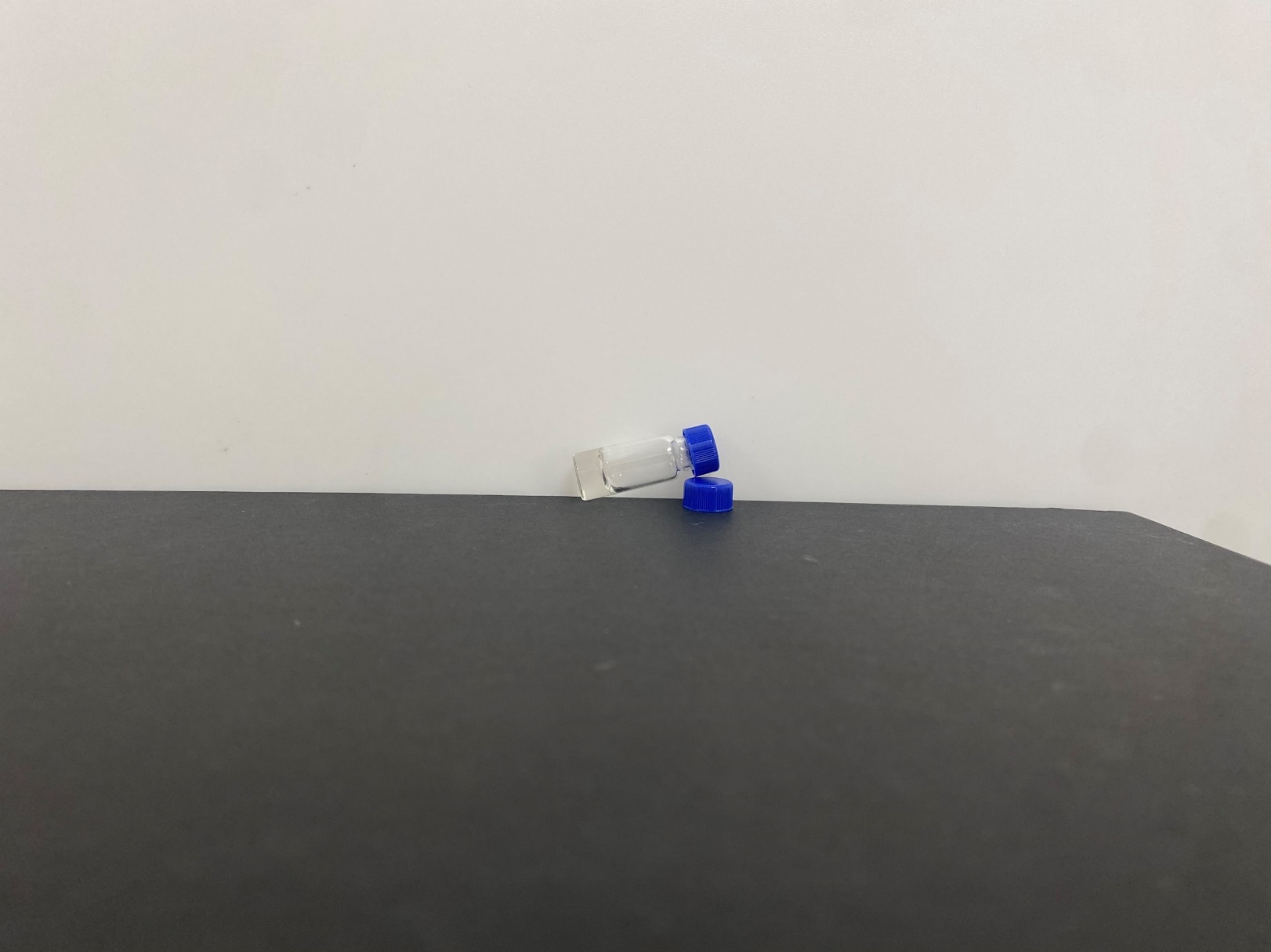

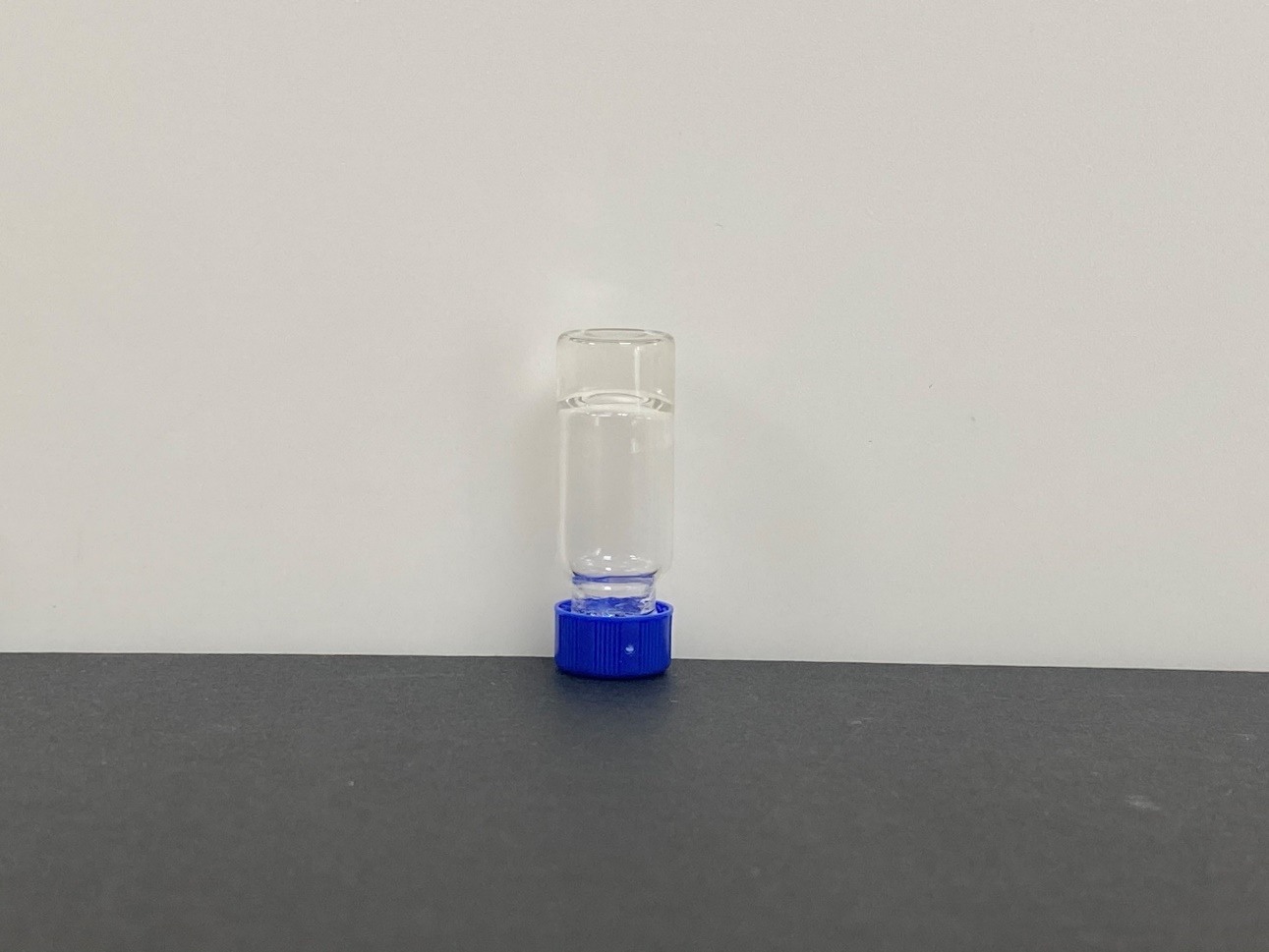

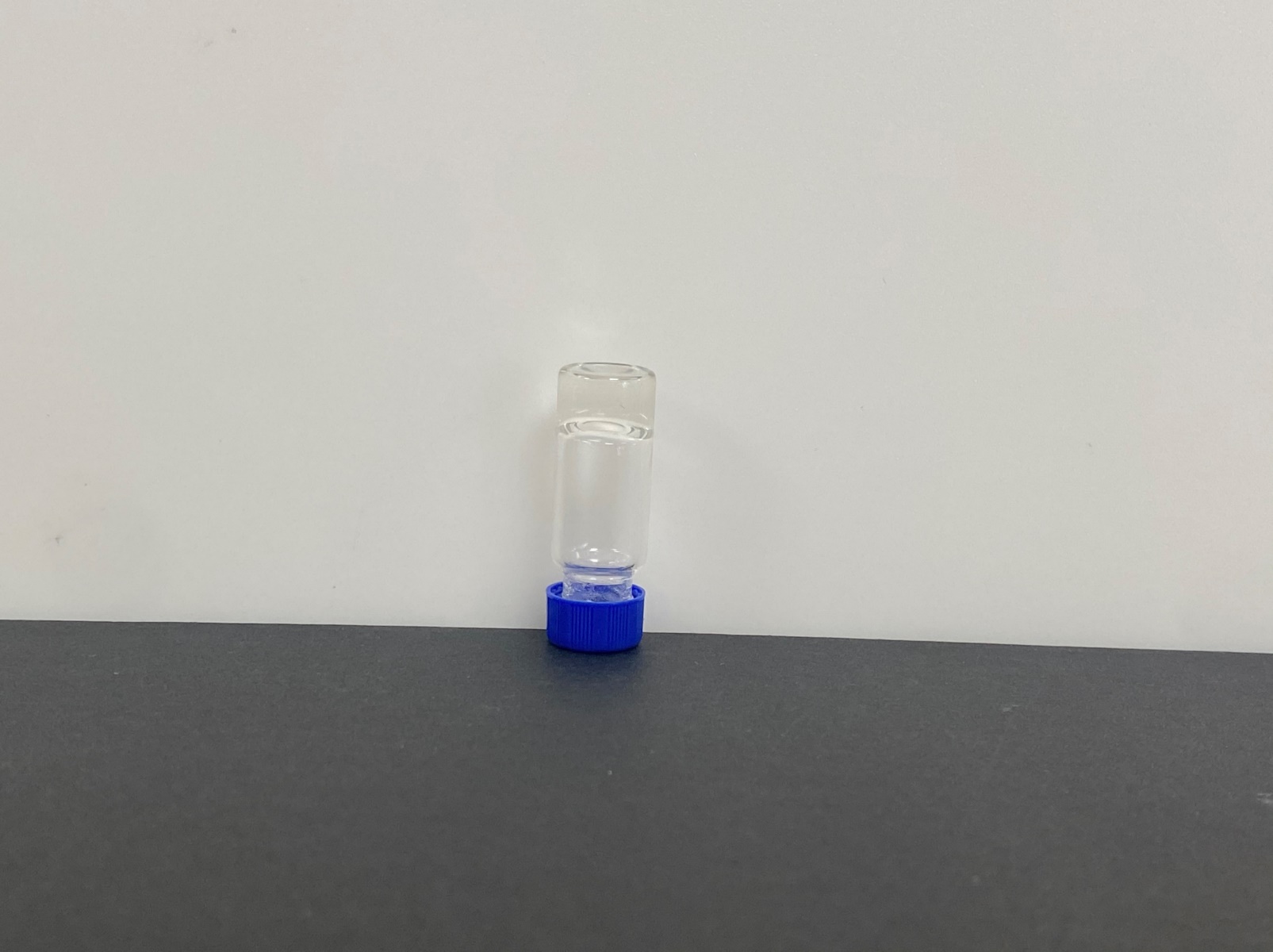

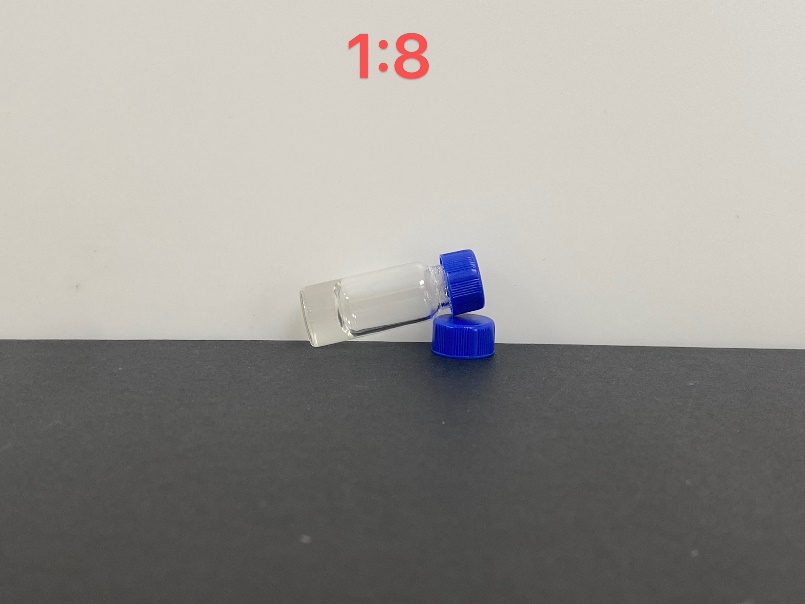


| **Target genes** | **Primer sequence (5’-3’)** |
| --- | --- |
| mouse-HO-1 | F: TCTGGATGGAGGGAGATACC  R: CAGCAGTCGTGGTCAGTCAA |
| mouse-IL-6 | F: ATAGTCCTTCCTACCCCAATTTCC  R: GATGAATTGGATGGTCTTGGTCC |
| mouse-IL-1β | F: TGGAGAGTGTGGATCCCAAG  R: GGTGCTGATGTACCAGTTGG |
| mouse-TNF-α | F: CTGAACTTCGGGGTGATCGG  R: GGCTTGTCACTCGAATTTTGAGA |
| mouse-IL-4 | F: GTCATCCTGCTCTTCTTTCTCGA  R: GTGCATGGCGTCCCTTCTC |
| mouse-IL-10 | F: GAGAAGCATGGCCCAGAAATC  R: GAGAAATCGATGACAGCGCC |
| mouse-CD163 | F: TGCTGTCACTAACGCTCCTG  R: TCATTCATGCTCCAGCCGTT |
| mouse-GAPDH | F: TGACCACAGTCCATGCCATC  R: GACGGACACATTGGGGGTAG |
